# Systematics of Old World *Odontacolus* Kieffer *s.l.* (Hymenoptera, Platygastridae *s.l.*): parasitoids of spider eggs

**DOI:** 10.3897/zookeys.314.3475

**Published:** 2013-07-03

**Authors:** Alejandro A. Valerio, Andrew D. Austin, Lubomír Masner, Norman F. Johnson

**Affiliations:** 1 Department of Evolution, Ecology & Organismal Biology, The Ohio State University, 1315 Kinnear Road, Columbus, OH 43212, U.S.A.; 2 Australian Centre for Evolutionary Biology and Biodiversity, School of Earth and Environmental Sciences, The University of Adelaide, S.A. 5005, Australia; 3 Agriculture and Agri-Food Canada, K.W. Neatby Building, Ottawa, ON K1A 0C6, Canada

**Keywords:** Baeini, *Cyphacolus*, *Idris*, *Ceratobaeus*, egg-parasitoid, phylogeny, spider host, ovipositor system

## Abstract

The genera *Odontacolus* Kieffer and *Cyphacolus* Priesner are among the most distinctive platygastroid wasps because of their laterally compressed metasomal horn; however, their generic status has remained unclear. We present a morphological phylogenetic analysis comprising all 38 Old World and four Neotropical *Odontacolus* species and 13 *Cyphacolus* species, which demonstrates that the latter is monophyletic but nested within a somewhat poorly resolved *Odontacolus*. Based on these results *Cyphacolus* **syn. n.** is placed as a junior synonym of *Odontacolus* which is here redefined. The taxonomy of Old World *Odontacolus* *s.str.* is revised; the previously known species *Odontacolus longiceps* Kieffer (Seychelles), *Odontacolus markadicus* Veenakumari (India), *Odontacolus spinosus* (Dodd) (Australia) and *Odontacolus hackeri* (Dodd) (Australia) are re-described, and 32 new species are described: *Odontacolus africanus* Valerio & Austin **sp. n.** (Congo, Guinea, Kenya, Madagascar, Mozambique, South Africa, Uganda, Zimbabwe), *Odontacolus aldrovandii* Valerio & Austin **sp. n.** (Nepal), *Odontacolus anningae* Valerio & Austin **sp. n.** (Cameroon), *Odontacolus australiensis* Valerio & Austin **sp. n.** (Australia), *Odontacolus baeri* Valerio & Austin **sp. n.** (Australia), *Odontacolus berryae* Valerio & Austin **sp. n.** (Australia, New Zealand, Norfolk Island), *Odontacolus bosei* Valerio & Austin **sp. n.** (India, Malaysia, Sri Lanka), *Odontacolus cardaleae* Valerio & Austin **sp. n.** (Australia), *Odontacolus darwini* Valerio & Austin **sp. n.** (Thailand), *Odontacolus dayi* Valerio & Austin **sp. n.** (Indonesia), *Odontacolus gallowayi* Valerio & Austin **sp. n.** (Australia), *Odontacolus gentingensis* Valerio & Austin **sp. n.** (Malaysia), *Odontacolus guineensis* Valerio & Austin **sp. n.** (Guinea), *Odontacolus harveyi* Valerio & Austin **sp. n.** (Australia), *Odontacolus heratyi* Valerio & Austin **sp. n.** (Fiji), *Odontacolus heydoni* Valerio & Austin **sp. n.** (Malaysia, Thailand), *Odontacolus irwini* Valerio & Austin **sp. n.** (Fiji), *Odontacolus jacksonae* Valerio & Austin **sp. n.** (Cameroon, Guinea, Madagascar), *Odontacolus kiau* Valerio & Austin **sp. n.** (Papua New Guinea), *Odontacolus lamarcki* Valerio & Austin **sp. n.** (Thailand), *Odontacolus madagascarensis* Valerio & Austin **sp. n.** (Madagascar), *Odontacolus mayri* Valerio & Austin **sp. n.** (Indonesia, Thailand), *Odontacolus mot* Valerio & Austin **sp. n.** (India), *Odontacolus noyesi* Valerio & Austin **sp. n.** (India, Indonesia), *Odontacolus pintoi* Valerio & Austin **sp. n.** (Australia, New Zealand, Norfolk Island), *Odontacolus schlingeri* Valerio & Austin **sp. n.** (Fiji), *Odontacolus sharkeyi* Valerio & Austin **sp. n.** (Thailand), *Odontacolus veroae* Valerio & Austin **sp. n.** (Fiji), *Odontacolus wallacei* Valerio & Austin **sp. n.** (Australia, Indonesia, Malawi, Papua New Guinea), *Odontacolus whitfieldi* Valerio & Austin **sp. n.** (China, India, Indonesia, Sulawesi, Malaysia, Thailand, Vietnam), *Odontacolus zborowskii* Valerio & Austin **sp. n.** (Australia), and *Odontacolus zimi* Valerio & Austin **sp. n.** (Madagascar). In addition, all species of *Cyphacolus* are here transferred to *Odontacolus*: *Odontacolus asheri* (Valerio, Masner & Austin) **comb. n.** (Sri Lanka), *Odontacolus axfordi* (Valerio, Masner & Austin) **comb. n.** (Australia), *Odontacolus bhowaliensis* (Mani & Mukerjee) **comb. n.** (India), *Odontacolus bouceki* (Austin & Iqbal) **comb. n.** (Australia), *Odontacolus copelandi* (Valerio, Masner & Austin) **comb. n.** (Kenya, Nigeria, Zimbabwe, Thailand), *Odontacolus diazae* (Valerio, Masner & Austin) **comb. n.** (Kenya), *Odontacolus harteni* (Valerio, Masner & Austin) **comb. n.** (Yemen, Ivory Coast, Paskistan), *Odontacolus jenningsi* (Valerio, Masner & Austin) **comb. n.** (Australia), *Odontacolus leblanci* (Valerio, Masner & Austin) **comb. n.** (Guinea), *Odontacolus lucianae* (Valerio, Masner & Austin) **comb. n.** (Ivory Coast, Madagascar, South Africa, Swaziland, Zimbabwe), *Odontacolus normani* (Valerio, Masner & Austin) **comb. n.** (India, United Arab Emirates), *Odontacolus sallyae* (Valerio, Masner & Austin) **comb. n.** (Australia), *Odontacolus tessae* (Valerio, Masner & Austin) **comb. n.** (Australia), *Odontacolus tullyae* (Valerio, Masner & Austin) **comb. n.** (Australia), *Odontacolus veniprivus* (Priesner) **comb. n.** (Egypt), and *Odontacolus watshami* (Valerio, Masner & Austin) **comb. n.** (Africa, Madagascar). Two species of *Odontacolus* are transferred to the genus *Idris* Förster: *Idris longispinosus* (Girault) **comb. n.** and *Idris amoenus* (Kononova) **comb. n.**, and *Odontacolus doddi* Austin **syn. n.** is placed as a junior synonym of *Odontacolus spinosus* (Dodd). *Odontacolus markadicus*, previously only known from India, is here recorded from Brunei, Malaysia, Sri Lanka, Thailand and Vietnam. The relationships, distribution and biology of *Odontacolus* are discussed, and a key is provided to identify all species.

## Introduction

The genera *Odontacolus* Kieffer and *Cyphacolus* Priesner are easily recognized from other platygastroid wasps by the obvious lateral compression of the T1 horn in the female. Previously considered to be relatively rare based on material available in collections, recent intensive collecting using Malaise and yellow-pan traps has revealed that some species of *Odontacolus* are moderately common. The likely phylogenetic affinities of the genera based on morphology are somewhat equivocal (Austin and Iqbal 2005; Valerio et al. 2010); however, a preliminary molecular analysis (Carey et al. 2006) has confirmed that *Odontacolus* belongs to the Baeini and is related to *Baeus* Haliday and *Idris* Förster *s.l.*, the latter representing a huge assemblage, possibly numbering in excess of a thousand species. Host data are scant, but available records confirm that, like all members of the Baeini, both genera are endoparasitoids of spider eggs (e.g. Masner 1976; Austin 1984, 1985; Galloway and Austin 1984; Stevens and Austin 2007; Valerio et al. 2010).

**Figure 1.**
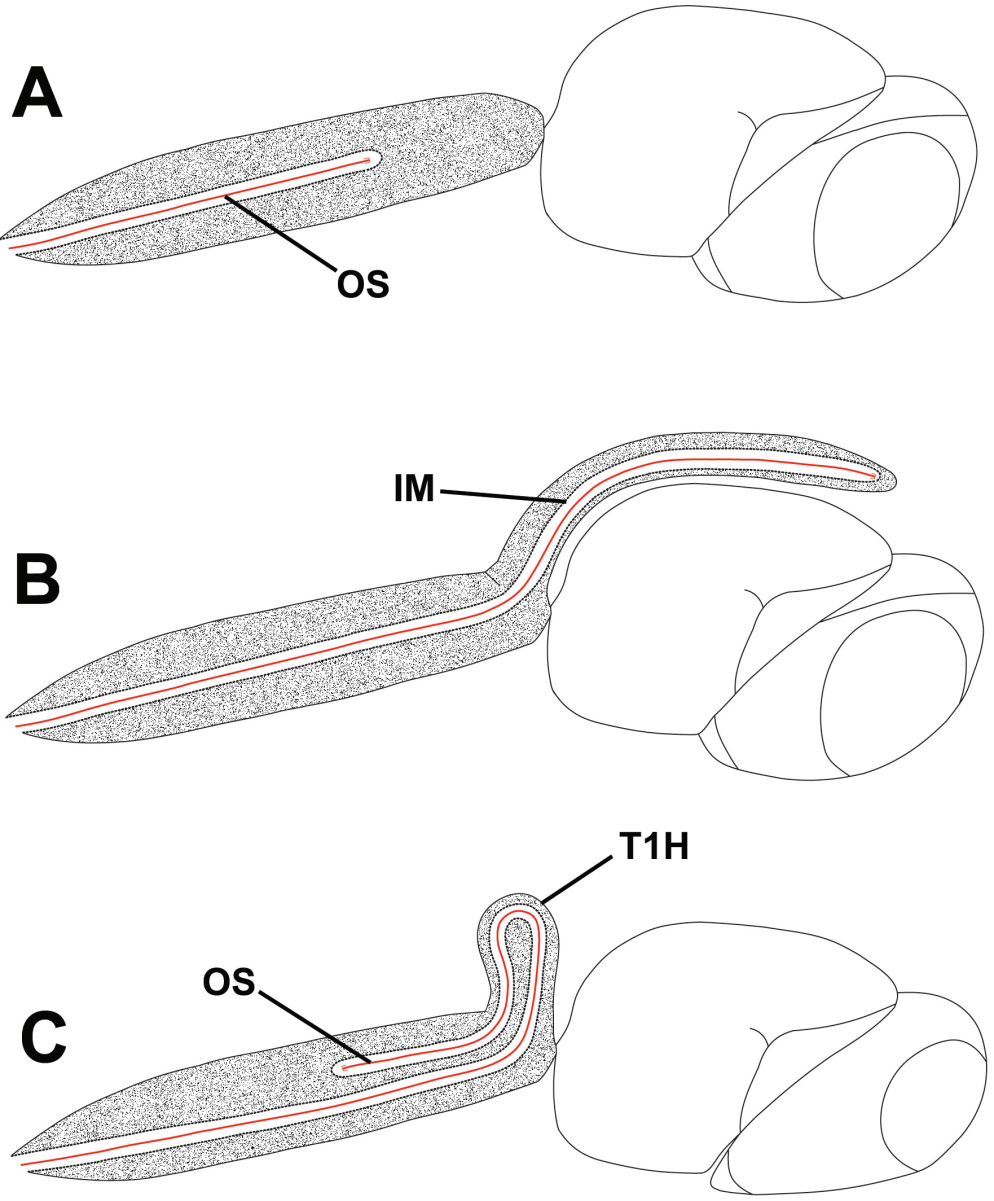
Representation of theexternal shape of T1 horn (T1H), shape of the ovipositor shaft (OS) and invaginated intersegmental membrane (IM) for *Idris* sp. (**A**) *Idris (Ceratobaeus)* sp. (**B**) and *Odontacolus* sp. (**C**).

The taxonomic status of both genera has largely been stable since their description, in part because they have received little attention. *Odontacolus* was originally described by Kieffer (1910a) for *Odontacolus longiceps* from the Seychelle Islands with only a few species described since then. *Cyphacolus* was described by Priesner (1951) for *Cyphacolus veniprivus* from Egypt, but recently Valerio et al. (2010) have completely revised the genus and recognized 16 species distributed from Africa, the Middle East, India, south-east Asia to Australia. *Cyphacolus* + *Odontacolus* clearly form a monophyletic group within the Baeini, based on the unique shape of the T1 horn, the presence of large blunt spines on the propodeum, and the subpedunculate metasoma. As discussed by Austin and Iqbal (2005), the shape of the horn is apparently linked to the functional mechanics of the ovipositor system, as the ovipositor is retracted within the metasoma and is curled around in the curved head of the horn so that it forms an elongate, U-shape (Austin 1983; Valerio et al. 2010) (Fig. 1C). However, although *Cyphacolus* is putatively monophyletic based on the fore wing being spoon-shaped and contoured to the convex surface of metasoma (subelytriform), with a dark infuscate patch at the fore wing margin, and the distal venation being absent, *Odontacolus* does not possess any obvious synapomorphies, and thus may be paraphyletic with respect to the former genus.

The current study builds on our recent taxonomic treatment of *Cyphacolus* (Valerio et al. 2010) by undertaking a phylogenetic analysis to examine the relationships between the two genera and their component species using a larger morphological dataset, and by completing a taxonomic revision of *Odontacolus* *s.l.* for the Old World, describing a large number of new species, and documenting their biology and distribution.

The contributions of the individual authors are as follows; A.A. Valerio: character definition, character development, character coding, imaging, species concept development; key development, capture of specimen data, phylogenetic analysis and manuscript preparation; A.D. Austin: species concept development, manuscript preparation, key development, and character coding; L. Masner: generic characters, character definition, character development, and specimen acquisition; N.F. Johnson: generic characters, distributional information and manuscript preparation.

## Material and methods

**Specimens examined**. The following collections provided specimens for this study or are mentioned in the text: Australian Museum, Sydney (AMSA)^1^; Australian National Insect Collection, Canberra (ANIC)^2^; The Natural History Museum, London (BMNH)^3^; Bernice P. Bishop Museum, Honolulu (BPBM); California Academy of Sciences, San Francisco (CASC)^4^; Canadian National Collection of Insects, Ottawa (CNCI)^5^; Essig Museum of Entomology, Berkeley, California (EMEC)^6^; Fiji National Insect Collection, Suva (FNIC)^7^; Hungarian Natural History Museum, Budapest (HNHM)^8^; Indian Agriculture Research Institute, National Pusa Collections, New Delhi (INPC)^9^; Lincoln University, Lincoln, New Zealand (LUNZ)^10^; National Bureau of Agriculturally Important Insects, India (NBAII)^11^; National Museum of Kenya, Nairobi (NMKE)^12^; New Zealand Arthropod Collection, Aukland (NZAC)^13^; C.A. Triplehorn Insect Collection, Ohio State University, Columbus (OSUC)^14^; Queensland Primary Industries and Fisheries Insect Collection, Brisbane (QDPC)^15^; Queensland Museum, Brisbane (QMBA)^16^; Queen Sirikit Botanic Garden, Chiang Mai, Thailand (QSBG)^17^; Nationaal Natuurhistorisch Museum, Leiden (RMNH)^18^; Royal Ontario Museum, Toronto (ROME)^19^; South Australian Museum, Adelaide (SAMA)^20^; Ukrainian Academy of Sciences, Kiev (UASK)^21^; University of California, Riverside (UCRC)^22^; National Museum of Natural History, Washington, DC (USNM)^23^; Western Australian Museum, Perth (WAMP)^24^; Waite Insect and Nematode Collection, University of Adelaide, Adelaide (WINC)^25^.

**Project information.** This work is a product of the Platygastroidea Planetary Biodiversity Inventory, a project funded by the U.S. National Science Foundation (N.F. Johnson, The Ohio State University, and A.D. Austin, The University of Adelaide, Principal Investigators). One of the primary objectives of this project is to use biodiversity informatics tools to accelerate the taxonomic process and to make real-time collaboration possible within the community of researchers with appropriate expertise. Details on the data associated with these specimens may be accessed at the following link: purl.oclc.org/NET/hymenoptera/hol, and entering the identifier (e.g., OSUC 231234). All the life sciences identifiers (LSIDs) may be resolved at http://lsid.tdwg.org (i.e. urn:lsid:zoobank.org:act:85B4E914-30E6-481C-8678-766A449E5B62). Species descriptions were developed and generated using the cybertool vSysLab^26^ (ver. 1.5) (Johnson 2010). The format is “Character: Character state”. Polymorphic characters have the states separated by semicolons.

The specimen localities, except for the holotypes, are not included in full in the article’s text to avoid repetition of the data and to save publication space. The full collecting data for specimens are available at Hymenoptera On-Line (http://hol.osu.edu), the Global Biodiversity Information Facility (http://www.gbif.org) and in the Darwin Core Archive^27^ file available as a supplement to this paper.

**Terms**. Morphological terminology follows Masner (1980) and Mikó et al. (2007) except for the term “lagrimal” (abbreviated as ‘lag’ in figures and derived from the Spanish word “lagrimal”) that is presented here and defined as a subtriangular area between the malar sulcus and the compound eye. Normally the malar sulcus expands in a ‘V’ shape towards the compound eye in this area. The structure was first observed in *Odontacolus gentingensis* sp. n. (Fig. 36). Abbreviations used in text: antennomeres (A1, A2, etc); anterior sterna carina (ASC); furrow (Frw); metasomal tergites (T1, T2, etc); metasomal sternites (S1, S2, etc); occipital carina (occ).

Appendix I list terms associated with identifiers in the Hymenoptera Anatomy Ontology (http://glossary.hymao.org). Identifiers in the format HAO_XXXXXXX represent concepts in the HAO in August 2012 and are provided to enable readers to confirm their understanding of the concepts being referenced. The identifier can also be used as a URI (universal resource identifier) by appending the identifier to ‘http://purl.obolibrary.org/obo/’ (e.g. http://purl.obolibrary.org/obo/HAO_0000124), which resolves to the HAO’s community-based resource that includes additional images, notes, and other metadata. The external hyperlinks are explicitly cited in the endnotes so that users of the printed version of the paper have access to the same resources. As possible the external information conforms to standards developed and maintained through the organization Biodiversity Information Standards (Taxonomic Database Working Group). All new species have been registered with Zoobank (http://www.zoobank.org), and other taxonomic names, where appropriate, have been retrospectively registered.

**Illustrations and data citation.** Images were taken with a JVC 3 CCD camera (model KY-575U) attached to a Leica Z16 APO with a Planapo 1.0× objective alone or in combination with a 2× magnifying lens. Specimens were illuminated with a 4 channel LED dome light from Advanced Illumination. Figures were produced using Auto-Montage Pro versions 5.10 and post-processed with Adobe Photoshop CS5. All images are archived at Morphbank^28^ and in Specimage^29^ (the image database at The Ohio State University).

**Phylogenetic analysis.** For the phylogenetic analysis *Idris* *s. str.* (*Idris*_sp. 1, OSUC 233340, and *Idris*_sp. 2, OSUC 228297), *Idris (Ceratobaeus)* (*Idris*_sp. 3, OSUC 190640), and *Idris floris* (Kononova & Fursov) were used as outgroups; *Idris floris* was used to root the tree. The ingroup comprised 1) all 36 species of Old World *Odontacolus* *s.str*. treated herein, 2) 13 of the 16 species of *Cyphacolus* treated by Valerio et al. (2011), and 3) six Neotropical species: *Odontacolus flavissimus* Megyaszai and *Odontacolus szaboi* Megyaszai and four undescribed species: Od_sp. 4 (OSUC 246525), Od_sp. 5 (OSUC 246524), Od_sp. 6 (OSUC 253001) and Od_sp. 7 (OSUC 233343).

A total of 41 morphological characters were scored for the 55 ingroup and four outgroup taxa (see Appendices II and III). Only character 9 (shape of the medial area of the vertex) was uninformative under the parsimony criterion. Characters 37 (ovipositor shape) and 41 (number of antennal segments) where coded directly from specimens for some species, and from the literature for others (e.g. Masner 1976; Galloway and Austin 1984; Valerio et al. 2010). The analysis was performed using the software TNT - Tree analysis using New Technology ver. 1.1 (Goloboff et al. 2008) under the command ‘xmult’and all characters were treated as unordered. In addition to the Maximum Parsimony analysis an implied weighting analysis was performed (under options K=3, 5 and 7) to explore possible structure of the data matrix with the resulting tree(s). The trees obtained under TNT were edited using Adobe Illustrator CS5.

### Phylogeny

**Figure 2.**
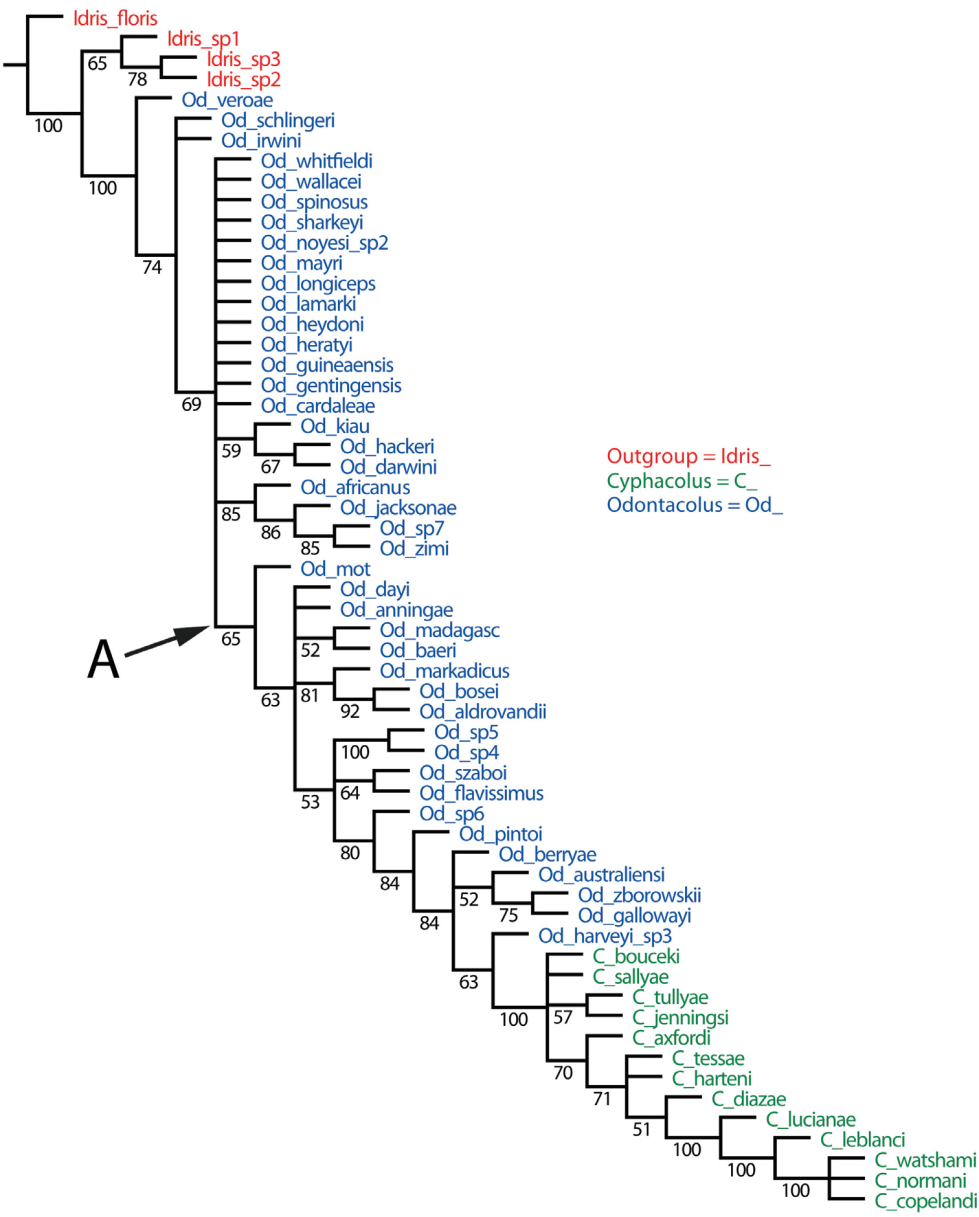
50% majority rule consensus tree (with frequencies above each node) for species of *Cyphacolus* and *Odontacolus* based on morphological characters (10,000 trees, 872 steps, CI=0.12, RI=0.27). Clade **A** is discussed in the text.

**Figure 3.**
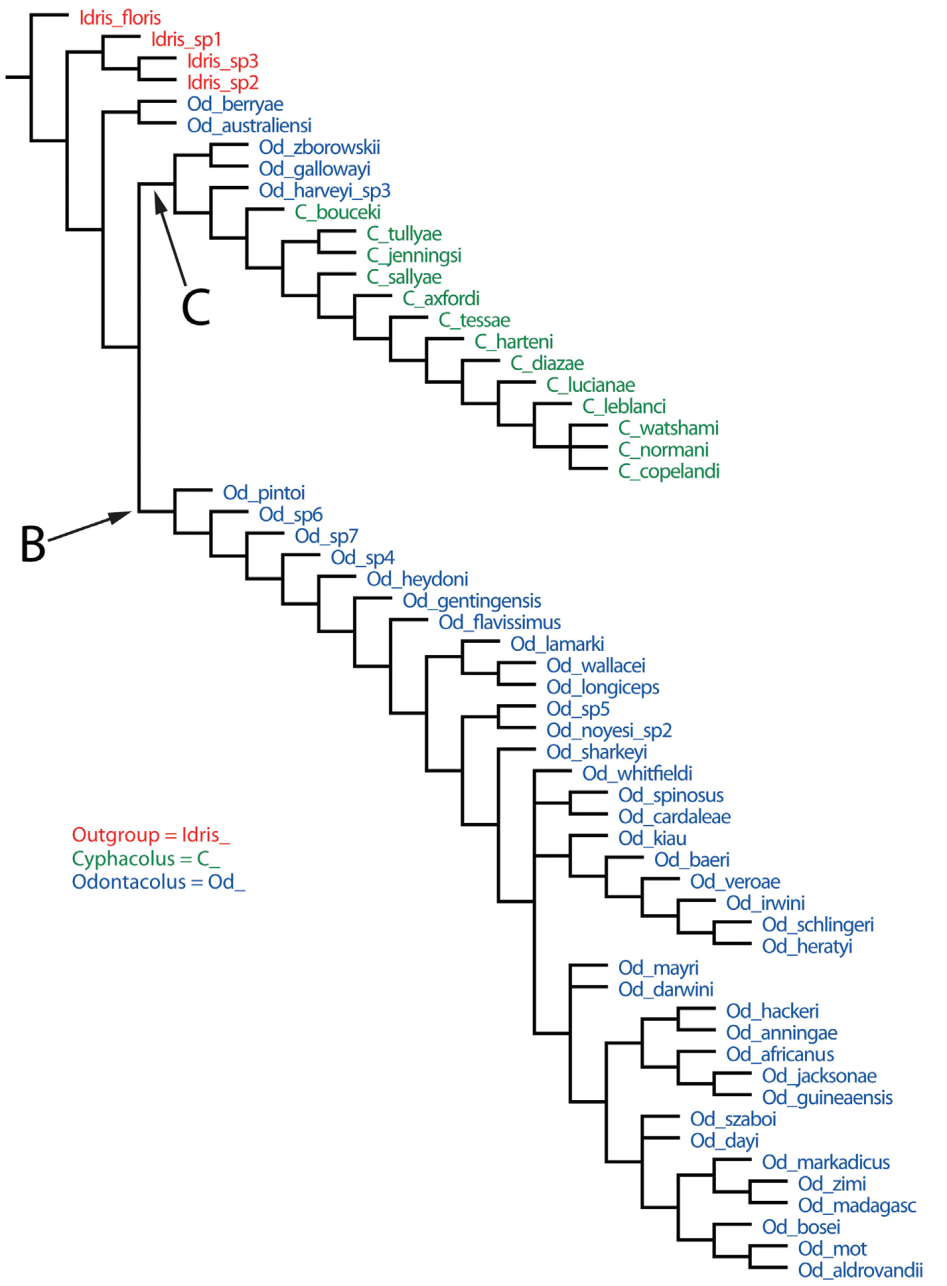
Phylogenetic consensus tree under Implied Weighting scheme for species of *Cyphacolus* and *Odontacolus* based on morphological characters (2376 trees, score value = 20.54, K=3). Clades B and C are discussed in the phylogeny section of the text.

Maximum parsimony analysis resulted in 10,000 trees of 348 steps (CI=0.18, RI=0.58). The strict consensus tree is largely unresolved except for a monophyletic group containing all 13 *Cyphacolus* species (tree not shown). The dataset clearly is very homoplasious, and so to explore possible relationships further, we compared the 50% majority rule consensus (MR) tree (Fig. 2) with that obtained from the implied weighting (IW, with K=3) analysis (4 trees, score=20.52) (Fig. 3). In both trees *Odontacolus* + *Cyphacolus* are monophyletic with respect to the outgroup taxa. In the MR tree three *Odontacolus* species from Fiji (*Odontacolus veroae*, *Odontacolus schlingeri*, *Odontacolus irwini*) form a grade at the base of the tree; there is a single large clade (A) with relative low support (65%) which contains *Cyphacolus* and 19 *Odontacolus* species, while the remaining taxa form a polytomy. In the IW tree the taxa in clade B largely correspond to clade A in the MR tree but with some exceptions, and Clade C encloses *Cyphacolus* plus *Odontacolus bouceki*, *Odontacolus gallowayi*, *Odontacolus harveyi* and *Odontacolus zborowskii*. The six Neotropical species occur in different places between the two trees but always with some taxa occurring among the Old World *Odontacolus* (i.e. *Odontacolus* sp. 7). In the MR tree four species (*Odontacolus flavissimus*, *Odontacolus szaboi*, *Odontacolus* sp. 4, *Odontacolus* sp. 5) are found in clade A but as a paraphyletic grade, while in the IW tree these same species are found througthout clade B, and *Odontacolus* sp. 6 and *Odontacolus* sp. 7 are found basally within Clade B next to *Odontacolus* sp. 4. Further, in the IW tree the Fijian species, which are among very few taxa that lack any traces of notauli, form a monophyletic group within clade B with *Odontacolus kiau* and *Odontacolus baeri*. This placement of these species would seem to make more sense biogeographically, given they are endemic to an isolated oceanic island, rather than three of them being at the base of the *Odontacolus* + *Cyphacolus* clade (as in the MR tree).

In both trees the same group of Australian species is sister to *Cyphacolus*, though they differ in their specific relationships. In the MR *Cyphacolus* seems to be the sister clade to the species *Odontacolus australiensis*, *Odontacolus berryae*, *Odontacolus harveyi*, *Odontacolus zborowskii* and *Odontacolus gallowayi*, while in the IW tree just *Odontacolus harveyi*, *Odontacolus zborowskii* and *Odontacolus gallowayi* form a paraphyletic clade being sister to *Cyphacolus* and *Odontacolus australiensis*, *Odontacolus berryae* are place at the base of the *Cyphacolus* + *Odontacolus* clade. Interestingly, these five species have a short stigmal vein (r-rs), the head is moderately short and strongly narrowed towards mouth, and they have somewhat setose eyes. These characters are also shared between other *Odontacolus* and *Cyphacolus* species. One of the main characters previously used to differentiate *Cyphacolus* from *Odontacolus* was its convex distal fore wing surface and conspicuous constriction at the base of the wing. However, it is now evident that this wing shape is also found in some *Odontacolus* species (e.g. *Odontacolus bosei*, *Odontacolus veroae*) although not as pronounced.

Additionally, the topology of the IW trees for K=5 and K=7 were similar to that for K=3, with *Cyphacolus* always being nested within *Odontacolus*, however the relationships among the most basal taxa for the clade *Odontacolus* + *Cyphacolus* clade varied among the trees. Also, the number of *Odontacolus* species placed as sister species to the *Cyphacolus* clade changes.

Although the relationships inferred from the data are not particularly robust, it is difficult to entertain a scenario in which all *Odontacolus* species form a monophyletic group with respect to *Cyphacolus*. Rather it seems highly likely that the latter genus is nested within *Odontacolus* and renders it paraphyletic. For this reason we place *Cyphacolus* as a junior synonym of *Odontacolus*, **syn. n.**, and refer to it hereafter as the *Odontacolus veniprivus* species group. All species of *Cyphacolus* are here transferred to *Odontacolus*: *Odontacolus asheri* (Valerio, Masner & Austin) **comb. n.** (Sri Lanka), *Odontacolus axfordi* (Valerio, Masner & Austin) **comb. n.** (Australia), *Odontacolus bhowaliensis* (Mani & Mukerjee) **comb. n.** (India), *Odontacolus bouceki* (Austin & Iqbal) **comb. n.** (Australia), *Odontacolus copelandi* (Valerio, Masner & Austin) **comb. n.** (Kenya, Nigeria, Zimbabwe, Thailand), *Odontacolus diazae* (Valerio, Masner & Austin) **comb. n.** (Kenya), *Odontacolus harteni* (Valerio, Masner & Austin) **comb. n.** (Yemen, Ivory Coast, Paskistan), *Odontacolus jenningsi* (Valerio, Masner & Austin) **comb. n.** (Australia), *Odontacolus leblanci* (Valerio, Masner & Austin) **comb. n.** (Guinea), *Odontacolus lucianae* (Valerio, Masner & Austin) **comb. n.** (Ivory Coast, Madagascar, South Africa, Swaziland, Zimbabwe), *Odontacolus normani* (Valerio, Masner & Austin) **comb. n.** (India, United Arab Emirates), *Odontacolus sallyae* (Valerio, Masner & Austin) **comb. n.** (Australia), *Odontacolus tessae* (Valerio, Masner & Austin) **comb. n.** (Australia), *Odontacolus tullyae* (Valerio, Masner & Austin) **comb. n.** (Australia), *Odontacolus veniprivus* (Priesner) **comb. n.** (Egypt), and *Odontacolus watshami* (Valerio, Masner & Austin) **comb. n.** (Africa, Madagascar).

## Taxonomy

### Odontacolus

Kieffer

urn:lsid:zoobank.org:act:846E9F7C-5402-4A83-8D9F-226A12BAEDD5

urn:lsid:biosci.ohio-state.edu:osuc_concepts:525

http://species-id.net/wiki/Odontacolus

Odontacolus Kieffer 1910a: 294. Original description. Type species: *Odontacolus longiceps* Kieffer, by monotypy and original designation. Kieffer 1910b: 100, 104 [description, list of species, keyed]; Kieffer 1912a: 54 [redescribed as new]; Kieffer 1912b: 88 [description]; Dodd 1914a: 59 [keyed]; Kieffer 1926: 132, 145 [description, keyed, key to species]; Muesebeck and Walkley 1956: 376 [citation of type species]; Masner 1976: 66 [description, key to *Odontacolus* Kieffer and *Ceratobaeus*Ashmead]; De Santis 1980: 313 [catalog of species of Brazil]; Austin 1981: 88 [diagnosis, synonymy, discussion of taxonomic status]; Galloway and Austin 1984: 6, 92 [description, list of species described from Australia, keyed]; Austin 1988: 176 [keyed]; Johnson 1992: 443 [catalog of world species]; Kononova 1992: 132 [description]; Masner and Denis 1996: 90 [keyed]; Kononova and Kozlov 2001: 340, 401 [description, keyed]; Loiácono and Margaría 2002: 557 [catalog of Brazilian species]; Austin and Iqbal 2005: 19 [discussion of relationships]; Valerio et al. 2010: 6 [phylogeny and discussion of relationships between *Cyphacolus* and *Odontacolus*]; Veenakumari and Mohanraj 2011 [key to species for females].
Ceratobaeoides Dodd 1913: 337. Original description. Type species: *Ceratobaeoides hackeri* Dodd, original designation. Dodd 1914a: 59 [keyed]; Dodd 1914b: 125 [description]; Kieffer 1926: 264, 273 [description, keyed, key to species]; Muesebeck and Walkley 1956: 341 [citation of type species]; Masner 1976: 65 [taxonomic status]; Austin 1981: 89 [junior synonym of *Odontacolus* Kieffer]; Carpenter 1992: 471 [fossil references].
Odontacolus urn:lsid:zoobank.org:act:BED2BB8F-3D3B-4D20-975E-4441CA26154F
Odontacolus urn:lsid:biosci.ohio-state.edu:osuc_concepts:9359
Cyphacolus Priesner 1951: 123. Original description. Type species: *Cyphacolus veniprivus* Priesner, by monotypy and original designation. [see Valerio et al. 2010: 4 for bibliography] **syn. n.**
Odontacolus urn:lsid:zoobank.org:act:85B4E914-30E6-481C-8678-766A449E5B62
Odontacolus urn:lsid:biosci.ohio-state.edu:osuc_concepts:468

#### Description.

*Female*. Tiny to small wasps, 1.01–2.38 mm in length; body slightly elongate, generally moderately slight in form.

*Head*. In anterior view varying in shape from suboval or subtriangular, to elongate and broad in buccal region. Frons smooth or sculptured, median area often defined by difference in sculpture compared to surrounding face, central keel present and often reaching almost to medial ocellus, sometimes virtually absent. Compound eyes large though appearing smaller in species with elongate buccal region, usually setose though sometimes setae tiny and eyes appearing glabrous. Mandibles with three teeth, normally subequal in size, sometimes with middle tooth slightly longer or lower tooth slightly shorter. Head in lateral view rather narrow, tapering to mouth, particularly in species with elongate malar region. Head in dorsal view curved around anterior mesosoma. Occiput well exposed. Ocelli usually large, touching or close to orbital margin. Occipital carina complete dorsally, sometimes faint because of surrounding sculpture. Antenna 7-segmented, clava large and appearing unsegmented or with distinct suture lines so that clava comprises 4 segments.

*Mesosoma*. Mesoscutum wider than long, usually flat or virtually so, sometimes dorsally convex. Notauli usually present as distinct grooves reaching no more than about half way to anterior margin of mesoscutum, sometimes virtually absent or hidden by coarse longitudinal sculpture. Mesoscutellum either flat or transverse, with posterior margin usually straight medially, or dorsally convex and semicircular or oval in shape. Propodeum with pair of broad, elongate spines which are blunt or truncate apically and flank the T1 horn.

*Wings*. Macropterous, never brachypterous. Fore wing narrow basally, broad in apical half, sometimes fore margin sinuate and surface of apical half convex (spoon-shaped) and molded to convex dorsal surface of metasoma (i.e. subelytriform). Fore wing venation with tubular submarginal (Sc+R) and stigmal veins (r-rs), marginal vien (C+R) very short, postmarginal vein (R1) very short or absent; in *Odontacolus veniprivus* species group venation lacking except for incomplete submarginal vein, with pronounced infuscate patch at position of marginal (C+R) and stigmal veins (r-rs). Fore wing color varying from hyaline to having dark infuscate bands.

*Metasoma* in dorsal view subpedunculate. T1 square or longitudinally slightly longer than wide (rarely more transverse), with parallel or slightly curved lateral margins. Metasoma widest in posterior half; in lateral view dorsal surface slightly to strongly convex. T1 with large, laterally compressed hornlike process (i.e. ellipsoidal in cross-section) which reaches to level of posterior mesocutellum or higher. T3 the largest tergite, slightly longer than T2, sometimes subequal in length with T2. Ovipositor at least 1.5× length of metasoma, with shaft curled back on itself within rounded head of the T1 horn. Gonoplacs elongate, approximately 0.75× length of metasoma.

*Male*. Antenna short, 11-segmented, often appearing to be 9- or 10-segmented as distal antenomeres are closely joined, distal antenna becoming progressively broader so as to be subclavate. Metasomal horn absent, but anterior part of T1 inflected upwards.

#### Diagnosis.

*Odontacolus* *s.l.* can be distinguished from all other genera of Platygastroidea by three unique characters: T1 of the female has a laterally compressed T1 horn (elliptical in cross-section); the ovipositor is retracted within the metasoma and curled around within the curved head of the T1 horn so that it forms an elongate, U-shape (compared to *Idris* species where it is straight or slightly curved, see Figs 1A–B); the propodeum in both sexes has a pair of spinelike flanges that (in the female) flank the T1 horn. In addition, all species are macropterous, never brachypterous, and the metasoma is subpedunculate in shape.

#### Distribution and regional diversity.

**Figure 4.**
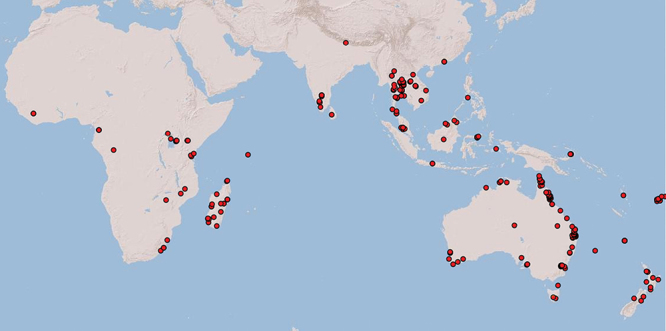
Distributional map of Old World *Odontacolus* species (not including *Cyphacolus* species as treated by Valerio et al. 2010).

*Odontacolus* is clearly more widespread than previously thought and, in the Old World, is found throughout Africa south of the Sahara, the Middle East (*Odontacolus veniprivus* group only; see Valerio et al. 2010), India, Sri Lanka, Nepal, south-east Asia (with one species extending north into China), and Australasia extending east in the Pacific to Fiji (Fig. 4, link to distribution map^30^). Australasia is the most species rich region (19 spp., four of which are endemic to Fiji), followed by India/south-east Asia (14 spp.) and Africa/Madagascar (11 spp.), the remainder of species have a broader distributions across regions.

#### Biology.

Like other members of the Baeini, *Odontacolus* species are endoparasitoids of spider eggs. However, of the 52 species here recorded from the Old World only five species have been reared. Veenakumari and Mohanraj (2011) record *Odontacolus markadicus* from eggs of an unknown salticid spider in southern India; *Odontacolus pintoi* has been reared from *Clubiona cycladata* Simon from under tree bark in South Australia (recorded as *Odontacolus* sp. in Austin (1984, 1985) (specimens in WINC); *Odontacolus berryae* has been reared from eggs of the salticid *Trite planiceps* Simon (specimens in LUNZ); *Odontacolus whitfieldi* has been reared from an unknown spider from China (specimens in UCRC), while *Odontacolus harteni* has also been reared from an unknown spider from Pakistan (specimens in BMNH) (Valerio et al. 2010). Although hosts are only from two spider families, Clubionidae and Salticidae, it is likely that *Odontacolus* species attack a much greater range of host spiders given the number of species and diversity of habitats from which they have been collected (e.g. rainforest, tropic dryforest, eucalypt forest, coastal dunes, mangroves, grasslands, etc.; see Appendix IV).

Although nothing is known about the ovipositional behavior of *Odontacolus* species, we predict that females employ their long ovipositor to parasitize host eggs from outside the egg sac, by pushing the ovipositor through the silk wall, in a similar way as has been described for *Idris (Ceratobaeus)* species (Austin 1984, 1985) that also have a T1 horn, albeit of a different shape (Figs 1B, C). Although documented for only a few species, the stategy of *Baeus* species (Stevens and Austin 2007), *Idris* *s.str.* and other baeines that lack a T1 horn, is to burrow through the silk wall of the egg sac and oviposit into eggs while in direct contact with them.

#### Species excluded from *Odontacolus*.

The previously described species *Odontacolus longispinosus* Girault (Fig. 1) and *Odontacolus amoenus* Kononova are here transferred to the genus *Idris* Försterand should therefore be treated as **new combinations**. We were unable to borrow the holotype of *Odontacolus amoenus* but one of us (NFJ) during a recent visit to the UASK was able to examine a female paratype and confirm that the species does indeed belong to *Idris*.

#### Key to the females of Old World species of *Odontacolus* Kieffer

**Table:** 

1	Fore wing with tubular stigmal vein present, without dark infuscate patch or bands at this position (Figs 133, 238, 293–296)	2
–	Fore wing with stigmal vein always absent, with dark infuscate patch or band at this position (*Odontacolus veniprivus* species group - previously known as *Cyphacolus*; see Valerio et al. 2010 for key to species)	
2	Notauli completely absent (Fig. 11)	3
–	Notauli always present (Fig. 12), although sometimes very reduced in length to as little as 1/6 of mesoscutum length	6
3	Mesoscutum and mesoscutellum conspicuously flattened in lateral view (Fig. 13), setation extremely short and dense; posterior margin of mesoscutellum with well-defined, large crenulae; orbital carina almost touching occipital carina; metapleuron mainly smooth in lower half (Fiji)	*Odontacolus schlingeri* Valerio & Austin, sp. n.
–	Mesoscutum and mesoscutellum convex in lateral view (Fig. 14), setation long, sparse; posterior margin of mesoscutellum with small, dense crenulae; distance between orbital carina and occipital carina variable; metapleuron variable in sculpture from completely smooth to complete coriaceous	4
4	Mesoscutum and mesoscutellum rugulose-granulate (Fig. 11); distance between posterior ocellus and occipital carina approximately 0.5× ocellar diameter (Fiji)	*Odontacolus heratyi* Valerio & Austin, sp. n.
–	Mesoscutum and mesoscutellum granulate, never rugulose-granulate (Figs 15, 16); distance between posterior ocellus and occipital carina approximately 1× ocellar diameter	5
5	Posterior sublateral areas of mesoscutum with cicatrose sculpture, remainder of mesoscutum with dense, weak granulate sculpture (Fig. 15); body length 1.47–1.71 mm; mesoscutellum flat in lateral view (Fiji)	*Odontacolus veroae* Valerio & Austin, sp. n.
–	Mesoscutum covered with fine, weak granulate sculpture (Fig. 16); body size small (1.04–1.08 mm); mesoscutellum convex in lateral view (Fiji)	*Odontacolus irwini* Valerio & Austin, sp. n.
6	Netrion appearing absent, lateral margins of pronotum with extensive, conspicuous, longitudinal costae (Figs 17, 19)	7
–	Netrion well-defined, lateral margins of pronotum at most with fine costae around margin of netrion (Figs 18, 20)	12
7	Gena mostly smooth, shining, sometimes coriaceous in upper 1/3 (Fig. 22); head below eyes (in anterior view) elongate and broad; antennal scrobe smooth except for central keel (Fig. 243), without fan-like carinae (Australia, New Zealand, Norfolk Island)	*Odontacolus pintoi* Valerio & Austin, sp. n.
–	Gena weakly rugulose (Fig. 21), surface dull; head below eyes (in anterior view) elongate and narrowed towards mandibles; lower half of antennal scrobe almost completely sculptured, fan-like carinae present (Figs 93, 167, 285)	8
8	Notauli present as smooth furrows (Fig. 166); occipital carina simple, slightly angled near lateral ocelli to be nearly straight medially (Fig. 29); body mostly yellow (Australia)	*Odontacolus harveyi* Valerio & Austin, sp. n.
–	Notauli crenulate throughout length (Figs 94, 142, 286); occipital carina heavily ornamented, gently arcuate dorsally (Fig. 24); body color variable	9
9	Frons and mesoscutum rugulose, rugulae broad (Figs 285, 286); notaulus width greater than half the width of tegula, with clearly defined, large crenulae throughout; body yellow (Australia)	*Odontacolus zborowskii* Valerio & Austin, sp. n.
–	Frons smooth, mesoscutum granulose, never rugulose; notauli variable in width, crenulae present or not; body color variable	10
10	Body black or dark brown; mesoscutellum smooth medially, setation conspicuously long and somewhat sparse (Fig. 23); mesoscutum broadly smooth in posterior half; crenulae of notauli well-defined, few and broad (Fig. 24); (Australia, New Zealand, Norfolk Island)	*Odontacolus berryae* Valerio & Austin, sp. n.
–	Body yellow or brown; mesoscutellum without smooth areas, setation short and dense; mesoscutum completely sculptured; crenulae of notauli weak or broad (Figs 92, 140)	11
11	Body brown (Fig. 89); notaulus deeply impressed, with few, sparse crenulae (Fig. 25); (Australia)	*Odontacolus australiensis* Valerio & Austin, sp. n.
–	Body completely yellow (Fig. 137); notaulus shallow, with abundant crenulae (Fig. 140) (Australia)	*Odontacolus gallowayi* Valerio & Austin, sp. n.
12	Gena smooth ventrally (Fig. 26); lateral propodeal areas very densely, weakly rugulose (Fig. 30) (Madagascar)	*Odontacolus magadascarensis* Valerio & Austin, sp. n.
–	Gena completely sculptured ventrally; lateral propodeal areas variable in sculpture	13
13	Compound eyes unusually small, dorsoventral length less than 1/3 head height (Fig. 27); head appearing very elongate in lateral view (India, Indonesia)	*Odontacolus noyesi* Valerio & Austin, sp. n.
–	Compound eyes of normal size, dorsoventral length nearly 1/2 head height (Fig. 28); head shape in lateral view variable	14
14	Occipital and orbital carinae contiguous or separated at most by distance subequal to width of occipital carina; head in lateral view elongate; occipital carina well-defined laterally (Fig. 31)	15
–	Occipital carina and orbital carinae separated by distance greater than width of occipital carina (Fig. 32), if occipital carina close to ocular carina, then head broad and short, or occipital carina not well-defined laterally (Fig. 92)	18
15	Mesoscutellum medially depressed posteriorly (Fig. 33); mesoscutum rugulose-granulate (Figs 34, 152); S2 anterior carina present (Guinea)	*Odontacolus guineensis* Valerio & Austin, sp. n.
–	Mesoscutellum convex; mesoscutum weakly granulate (Figs 146, 188); S2 anterior carina present or absent	16
16	S2 anterior carina absent; lagrimal absent; metasoma yellow or pale brown (except T1 horn) contrasting with dark brown color of remainder of body (Madagascar)	*Odontacolus zimi* Valerio & Austin, sp. n.
–	S2 anterior carina present; lagrimal present (Figs 35, 36); body entirely yellow or dark brown	17
17	Ventral portion of antennal scrobe with fan-like carinae arising from malar space (Fig. 147); lagrimal large, conspicuous (Fig. 36) (Malaysia)	*Odontacolus gentingensis* Valerio & Austin, sp. n.
–	Ventral portion of antennal scrobe without fan-like carinae (Fig. 189); lagrimal very reduced, inconspicuous (Fig. 35) (Cameroon, Guinea, Madagascar)	*Odontacolus jacksonae* Valerio & Austin, sp. n.
18	Central keel of frons absent (Fig. 38)	19
–	Central keel of frons present (Fig. 41)	20
19	S2 anterior carina interrupted medially (similar to Figs 65, 67); lateral ocelli minute, distance between lateral ocellus and occipital carina greater than 1.5× diameter of lateral ocellus (Fig. 37) (Australia, Indonesia, Malawi, Papua New Guinea)	*Odontacolus wallacei* Valerio & Austin, sp. n.
–	S2 anterior carina uninterrupted (Fig. 64); lateral ocelli normal in size, distance between lateral ocellus and occipital carina less than 0.8× diameter of lateral ocellus(Fig. 45) (Thailand)	*Odontacolus lamarcki* Valerio & Austin, sp. n.
20	Notauli crenulate (Figs 42, 118)	21
–	Notauli smooth (Fig. 44)	22
21	Ocelli large (Fig. 40); distance between lateral ocellus and occipital carina approximately 0.5× diameter of lateral ocellus (Fig. 40); T2 and T3 subequal in length (Fig. 176) (Malaysia, Thailand)	*Odontacolus heydoni* Valerio & Austin, sp. n.
–	Ocelli small (Fig. 39); distance between lateral ocellus and occipital carina approximately 1.5× diameter of lateral ocellus (Fig. 39); T2 clearly shorter than T3 (Fig. 116) (Australia)	*Odontacolus cardaleae* Valerio & Austin, sp. n.
22	Vertex slightly depressed mesally (Fig. 43); antennal scrobe weakly granulose laterally, nearly smooth medially; lateral ocelli large (Indonesia)	*Odontacolus dayi* Valerio & Austin, sp. n.
–	Vertex flat or slightly rounded, without mesal depression; antennal scrobe varying from completely smooth to completely rugulose; lateral ocelli variable in size	23
23	Antennal scrobe with transverse costae, covering at least 1/2 of frons (Figs 195, 225, 255, 261, 279)	24
–	Antennal scrobe without such extensive transverse elements, mostly smooth or only with coriaceous or granulose sculpture (Figs 111, 123, 231)	29
24	Fan-like costae present on frons, with a well-developed furrow at lower lateral areas of scrobe (Fig. 255); body completely dark brown (Fig. 251) (Thailand)	*Odontacolus sharkeyi* Valerio & Austin, sp. n.
–	Fan-like sculpture absent on frons, if weakly indicated, then without a furrow at lower lateral areas of scrobe (Figs 195, 261); body color variable (Figs 258, 192)	25
25	Vertex in anterior view flat (Fig. 159); lateral surface of T1 horn with U-shaped carinae, posterior surface covered with numerous broad, transverse costae; T1 horn short, broad (Fig. 155) (Australia)	*Odontacolus hackeri* (Dodd)
–	Vertex in anterior view convex; lateral surface of T1 horn transversely carinate, carinae becoming more curved dorsally, posterior surface with longitudinal carinae dorsally and remainder of T1 horn smooth or with very few narrow transverse costae on upper 1/3; T1 horn thin, elongate	26
26	Lateral ocelli small (Fig. 47), distance between lateral ocellus and occipital carina approximately 1.2× diameter of lateral ocellus (Australia)	*Odontacolus spinosus* (Dodd)
–	Lateral ocelli large, distance between occipital carina and lateral ocellus less than 0.8× diameter of lateral ocellus	27
27	S2 anterior carina absent (Fig. 59); mesal area of T2 with weak, longitudinal costae (Fig. 196) (Papua New Guinea)	*Odontacolus kiau* Valerio & Austin, sp. n.
–	S2 anterior carina present (similar to Figs 63, 64); mesal area of T2 smooth, without longitudinal costae, (Fig. 280)	28
28	Body yellow (Fig. 221); mesoscutum weakly granulose (Fig. 226) (Indonesia, Thailand)	*Odontacolus mayri* Valerio & Austin, sp. n.
–	Body dark brown (Fig. 276); mesoscutum with rugulae amid granulate sculpture (Fig. 280) (China, India, Indonesia, Malaysia, Thailand, Vietnam)	*Odontacolus whitfieldi* Valerio & Austin, sp. n.
29	S2 anterior carina interrupted medially (Figs 65, 66) or completely absent (Fig. 70)	30
–	S2 anterior carina present, continuous (Fig. 63)	32
30	S2 anterior carina absent (Fig. 70); distance between lateral ocellus and occipital carina approximately 1× diameter of lateral ocellus (Fig. 48); body yellow (Fig. 100) (Australia)	*Odontacolus baeri* Valerio & Austin, sp. n.
–	S2 anterior carina present, interrupted medially (Figs 65, 66); distance between lateral ocellus and occipital carina approximately 0.6× diameter of lateral ocellus; body color variable	31
31	Body dark brown; T1 laterally with abundant, fine, longitudinal costae, costae slightly curved, effaced (Congo, Guinea, Kenya, Madagascar, South Africa, Uganda, Zimbabwe)	*Odontacolus africanus* Valerio & Austin, sp. n.
–	Body completely yellow; T1 laterally with sparse, coarser, longitudinal costae, costae very straight, well-defined (Cameroon)	*Odontacolus anningae* Valerio & Austin, sp. n.
32	Mesosoma orange to light brown, contrasting with dark brown head and metasoma (Fig. 120); distance between lateral ocellus and occipital carina approximately 0.6× diameter of lateral ocellus (Thailand)	*Odontacolus darwini* Valerio & Austin, sp. n.
–	Body uniformly colored from yellow to dark brown (Figs 80, 109, 205, 217, 229); distance between lateral ocellus and occipital carina variable	33
33	Mesoscutum between notaulus and tegula almost completely smooth (Fig. 206); mesoscutellum with posterior 1/3 nearly smooth mesally (Seychelles islands)	*Odontacolus longiceps* Kieffer
–	Mesoscutum and mesoscutellum covered by well-defined sculpture, without smooth areas	34
34	Lagrimal present, conspicuous (Figs 53, 55); body robust, 1.75 mm or greater in length (Figs 79, 229)	35
–	Lagrimal absent or reduced (Figs 54, 56); body elongate, gracile, less than 1.75 mm in length (Fig. 215)	36
35	Head yellow; T1 light orange with horn contrastingly dark brown (Fig. 232); body size 1.92 mm (India)	*Odontacolus mot* Valerio & Austin, sp. n.
–	Head dark brown; T1 entirely yellow (Fig. 82), body size 1.75 mm (Fig. 77) (Nepal)	*Odontacolus aldrovandii* Valerio & Austin, sp. n.
36	Body completely black (Fig. 107); lateral portion of occipital carina with well-defined costulae throughout most of its length (Fig. 57) (India)	*Odontacolus bosei* Valerio & Austin, sp. n.
–	Body color a combination of yellow and dark brown, or completely yellow (Fig. 215); lateral portion of occipital carina mainly smooth (Fig. 58) (Brunei, India, Malaysia, Sri Lanka, Thailand, Vietnam)	*Odontacolus markadicus* Veenakumari

### Species descriptions

#### Odontacolus africanus

Valerio & Austin sp. n.

urn:lsid:zoobank.org:act:8B4944AC-A130-4024-846C-9FABEF79D61A

urn:lsid:biosci.ohio-state.edu:osuc_concepts:254265

http://species-id.net/wiki/Odontacolus_africanus

Figures 49 , 66 , 71–76 , 295 , 303 , 307 , 313 , 319–320 ; Morphbank ^31^

##### Description.

*Female*. Body length: 1.51–1.85 mm (n=4). Antenna color: completely yellow. Body color: completely dark brown. Coxae color: yellow. Leg color (excluding coxae): yellow. Fore wing color: completely hyaline.

*Head*. Size of compound eye: approximately 1/2× height of head. Head shape in lateral view: lower head elongate and broad at mouth, head appearing elongate and somewhat thin. Sculpture of antennal scrobe: largely smooth ventrally, sparsely granulate ventrally. Surface of torular triangle: slightly bulging. Development of central keel on frons: present, elongate (equal to or greater than 1/3× height of frons), but not reaching anterior ocellus. Sculpture on upper frons below anterior ocellus: granulose throughout. Sculpture of malar space: granulose throughout; with fan-like striae, striae not extending into antennal scrobe. Furrow at lateral portion of antennal scrobe: absent. Mesal surface of vertex: flat to weakly convex. Size of lateral ocelli: large. Distance between lateral ocellus and occipital carina: 0.5–1.2× maximum ocellar diameter. Lagrimal: absent or minute. Length of OOL: less than or equal to 1/3× width of ocellus. Sculpture of vertex: granulate. Sculpture of occipital carina: largely simple, at most with sparse weak crenulae medially. Distance from occipital carina to orbital carina: at least 2× width of occipital carina; slightly greater than width of occipital carina. Shape of occipital carina: simply arcuate medially. Sculpture of occiput: with weak, small granulae. Sculpture of gena: granulose.

*Mesosoma*. Dorsal mesosoma in lateral view: convex. Sculpture of pronotal cervical area: with small (at most as large as crenulae on anterior edge of mesoscutum), well-defined foveae. Sculpture of pronotal lateral area: largely smooth, dorsal margin with dense, weak punctulae. Netrion: present, smooth, linear. Notaulus: present, simple. Length of notaulus: approximately less than or equal to 1/3 of length of mesoscutum. Width of notaulus: narrow (notaulus width less than or equal to half the width of tegula). Sculpture of mesoscutum: anterior half coriaceous, otherwise densely granulose. Sculpture of mesoscutellum: with weak, fine, granulate sculpture. Mesoscutellar profile: mainly flat, anterior and posterior edge at same height or nearly so. Mesoscutellar shape: flat, not depressed. Lateral propodeal area: densely, finely rugulose. Shape of propodeal anterior spines: elongate, narrow, apex rounded. Sculpture of propodeum between anterior spines: smooth or largely smooth. Sculpture of ventral half of mesepisternum: weakly coriaceous. Sculpture of upper 1/4 of mesopleuron: with dense, well-defined longitudinally costate sculpture reaching just half of its width. Metapleuron sculpture: largely smooth except lower half with longitudinal carinae.

*Wings*. Stigmal vein: present, elongate, narrow. Campaniform sensilla at distal area of stigmal vein: present.

*Metasoma*. Shape of T1 horn: broad, short. Sculpture of upper portion of T1 horn: longitudinally carinate. Sculpture of posterior portion of T1 horn: longitudinally carinate. Lateral carinae on T2: present, poorly defined. Sculpture of T2: largely weakly coriaceous mixed with longitudinal costae, meson coriaceous. Sculpture of T3: anterior two-thirds longitudinally costate, otherwise coriaceous. Sculpture of S3–S6: mainly smooth, with sparse, setigerous punctulae. S2 anterior carina: present, cristate, interrupted medially.

*Male*. Body length: 1.36 – 1.60 mm (n=3). Body color: head, mesoscutum, edges of T1–T2, T4–T6 dark brown, reminder of terga light honey yellow as remainder of mesosoma. Sculpture of antennal scrobe: upper half with transverse carinae mixed with granulae, remainder of scrobe smooth. Shape and size of anterior ocellus: large, oblong in shape. Vertex posterior area sculpture: densely granulose. Occipital carina dorsal area: cristate, conspicuously present. Netrion: well-defined, suboval. Sculpture of mesepisternum: absent (smooth). Sculpture of pronotal lateral areas: with thin, longitudinal carinae. Length of fore wing stigmal vein: conspicuously elongate. Angle of stigmal vein in relation to anterior margin of fore wing: at an angle of approximately 45° with respect to anterior margin of wing. Sculpture of T2: mesal area with weal longitudinal carinae, remainder of tergum with longitudinal carinae mixed with coriaceous sculpture.

##### Diagnosis.

*Odontacolus africanus* is very similar to *Odontacolus anningae*, but the latter species can be identified by the body being completely yellow and the mesal area of T2 with a conspicuous smooth area, and the remainder of the tergum with thin, somewhat sparse, well-defined, longitudinal costae throughout their length, and without coriaceous sculpture in the background.

##### Etymology.

This species is named in reference to its broad distribution across southern and east Africa.

##### Link to distribution map.

32

##### Material examined.

Holotype female: **SOUTH AFRICA**: KwaZulu-Natal Prov., Eshowe, VI-1926, R. E. Turner, OSUC 238419 (deposited in BMNH). *Paratypes*: (88 females, 3 males) **CAMEROON**: 1 female, OSUC 238442 (BMNH). **CONGO**: 1 female, OSUC 381663 (OSUC). **GUINEA**: 1 female, OSUC 238724 (CNCI). **KENYA**: 64 females, 2 males, OSUC 321891 (BMNH); OSUC 238444, 238447, 238519, 238550, 238572, 238576–238577, 238723, 238725, 238729, 238732–238733, 238735–238737, 238739, 238741–238742, 238747–238750, 238752–238755, 238758–238764, 321886 (CNCI); OSUC 238522, 238555, 238565, 238567–238568, 238573, 238582, 238722, 238726, 238730, 238738, 238757 (NMKE); OSUC 238520, 238740, 238745, 238751 (OSUC); OSUC 238438, 238514, 238727, 238731, 238734, 238743, 238746, 238765–238766, 267295–267297, 381662 (USNM); OSUC 238744, 412089 (WINC). **MADAGASCAR**: 12 females, 1 male, OSUC 238436–238437, 321888 (BMNH); CASENT 2042644–2042645, 2134801, 2134824, 2135994, 2137871, 2137902 (CASC); CASENT 2042643, 2079135, 2135721 (OSUC). **MOZAMBIQUE**: 1 female, OSUC 339606 (CNCI). **SOUTH AFRICA**: 4 females, OSUC 321889–321890 (BMNH); OSUC 238720–238721 (CNCI). **UGANDA**: 2 females, OSUC 238427, 238446 (CNCI). **ZIMBABWE**: 2 females, OSUC 238425, 238439 (CNCI).

##### Comments.

The holotype has the posterior right leg detached from the body and glued to the triangle; the right side wings are detached and in a gelatin capsule; the left antenna is missing. Most of the paratypes are in perfect condition.

In some female specimens the color pattern differs from that described above, the metasoma is a slightly lighter tone of dark brown compared to the head or the mesosoma (i.e. OSUC 238763). Additionally, the mesal area of T2 normally is covered by fine, dense, longitudinal costae (as in the holotype) but some specimens exhibit a smooth area with weak coriaceous sculpture in the background (in the absence of longitudinal costae), or a narrow smooth area with the lateral areas of the tergite with weak, longitudinal costae mixed with weak coriaceous sculpture in the background.

#### Odontacolus aldrovandii

Valerio & Austin sp. n.

urn:lsid:zoobank.org:act:155D7A01-D442-4E06-B1FB-F36E3B5F50CC

urn:lsid:biosci.ohio-state.edu:osuc_concepts:254276

http://species-id.net/wiki/Odontacolus_aldrovandii

Figures 53 , 77–82 ; Morphbank ^33^

##### Description.

*Female*. Body length: 1.75 mm (n=1). Antenna color: A1, A5–A7 light yellow, remainder of antenna light honey yellow. Body color: completely dark brown. Coxae color: yellow. Leg color (excluding coxae): yellow. Fore wing color: completely hyaline.

*Head*. Size of compound eye: approximately 1/2× height of head. Head shape in lateral view: lower head elongate and broad at mouth, head appearing elongate and somewhat thin. Sculpture of antennal scrobe: weakly coriaceous throughout. Surface of torular triangle: slightly bulging. Development of central keel on frons: present, elongate, reaching anterior ocellus. Sculpture on upper frons below anterior ocellus: with sparse, transverse costae mixed with weak, dense granulae. Sculpture of malar space: with weak rugulose sculpture mixed with granulate sculpture. Furrow at lateral portion of antennal scrobe: absent. Shape of medial area of vertexmedial area of vertex: flat to weakly convex. Size of lateral ocelli: large. Distance between lateral ocellus and occipital carina: 0.5–1.2× maximum ocellar diameter. Lagrimal: present, well-defined but small (length less than 0.3 of malar sulcus length). Length of OOL: less than or equal to 1/3× width of ocellus. Sculpture of vertex: granulate. Sculpture of occipital carina: weakly crenulate throughout. Distance from occipital carina to orbital carina: at least 2× width of occipital carina. Shape of occipital carina: simply arcuate medially. Sculpture of occiput: with weak, small granulae. Sculpture of gena: granulose.

*Mesosoma*. Dorsal mesosoma in lateral view: convex. Sculpture of pronotal cervical area: with small (at most as large as crenulae on anterior edge of mesoscutum), well-defined foveae. Sculpture of pronotal lateral area: largely granulose, ventral 1/4 with broad costae. Netrion: present, smooth, well developed, sub-obovate. Notaulus: present, simple. Length of notaulus: approximately less than or equal to 1/3 of length of mesoscutum. Width of notaulus: narrow (notaulus width less than or equal to half the width of tegula). Sculpture of mesoscutum: weakly rugulose mixed with weak granulae. Sculpture of mesoscutellum: granulose. Mesoscutellar profile: elevated, anterior margin higher than posterior. Mesoscutellar shape: flat, not depressed. Lateral propodeal area: sparsely transversely carinate. Shape of propodeal anterior spine: elongate, narrow, apex rounded. Sculpture of propodeum between anterior spines: smooth or largely smooth. Sculpture of ventral half of mesepisternum: weakly coriaceous. Sculpture of upper 1/4 of mesopleuron: densely longitudinally costate across entire width. Metapleural sculpture: largely smooth except lower half with longitudinal carinae.

*Wings*. Stigmal vein: present, elongate, narrow. Campaniform sensilla at distal area of stigmal vein: present.

*Metasoma*. Shape of T1 horn: narrow, short. Sculpture of upper portion of T1 horn: longitudinally carinate. Sculpture of posterior portion of T1 horn: largely smooth, with sparse longitudinal carinae. Lateral carinae on T2: present, well-defined. Sculpture of T2: finely longitudinally costate, granulate laterally. Sculpture of T3: anterior third weakly costate sublaterally, weakly coriaceous mesally, otherwise granulose. Sculpture of S3–S6: S3 weakly coriaceous, S4–S6 mainly smooth with few, sparse, setigerous punctulae sculpture. S2 anterior carina: present, cristate, uninterrupted.

*Male*. Unknown.

##### Diagnosis.

This is the only species of *Odontacolus* known from Nepal so far. It has a small but evident lagrimal as in *Odontacolus mot*; however, the lagrimal is not as large as in *Odontacolus gentingensis*. *Odontacolus aldrovandii* can be distinguished from *Odontacolus mot* by its smaller body size and the honey yellow coloration of the metasoma. In *Odontacolus mot* the metasoma in mainly dark brown except for the light orange T1.

##### Etymology.

This species is named to honor the Renaissance Italian naturalist Ulisse Aldrovandi. The epithet is used as noun in the genitive case.

##### Link to distribution map.

34

##### Material examined.

Holotype female: **NEPAL** nr. Birganj, Lothar, 450ft. 13–19.ix.1967, Malaise trap No.84, Canadian Nepal Expedition, OSUC 239210 (deposited in CNCI).

##### Comments.

The holotype is in good condition except that the right hind wing and right antennal clava are missing.

#### Odontacolus anningae

Valerio & Austin sp. n.

urn:lsid:zoobank.org:act:B77B5DD6-5506-40AE-AC8E-BFA080D09E00

urn:lsid:biosci.ohio-state.edu:osuc_concepts:281688

http://species-id.net/wiki/Odontacolus_anningae

Figures 49, 51 , 69 , 83–88 ; Morphbank ^35^

##### Description.

*Female*. Body length: 1.39 mm (n=1). Antenna color: A1 yellow, otherwise dark brown. Body color: completely yellow. Coxae color: yellow. Leg color (excluding coxae): yellow. Fore wing color: slightly infuscate throughout.

*Head*. Size of compound eye: approximately 1/2× height of head. Head shape in lateral view: lower head elongate and broad at mouth, head appearing elongate and somewhat thin. Sculpture of antennal scrobe: largely smooth ventrally, sparsely granulate ventrally. Surface of torular triangle: slightly bulging. Development of central keel on frons: present, elongate (equal to or greater than 1/3× height of frons), but not reaching anterior ocellus. Sculpture on upper frons below anterior ocellus: with sparse, transverse costae mixed with weak, dense granulae. Sculpture of malar space: coriaceous throughout, without fan-like striae. Furrow at lateral portion of antennal scrobe: absent. Shape of medial area of vertexmedial area of vertex: flat to weakly convex. Size of lateral ocelli: small. Distance between lateral ocellus and occipital carina: 0.5–1.2× maximum ocellar diameter. Lagrimal: absent or minute. Length of OOL: less than or equal to 1/3× width of ocellus. Sculpture of vertex: granulate. Sculpture of occipital carina: largely simple, at most with sparse weak crenulae medially. Distance from occipital carina to orbital carina: slightly greater than width of occipital carina. Shape of occipital carina: simply arcuate medially. Sculpture of occiput: with weak, small granulae. Sculpture of gena: granulose.

*Mesosoma*. Dorsal mesosoma in lateral view: convex. Sculpture of pronotal cervical area: with small (at most as large as crenulae on anterior edge of mesoscutum), well-defined foveae. Sculpture of pronotal lateral area: upper 1/3 granulose, lower 1/3 with transverse foveae, otherwise smooth. Netrion: present, smooth, linear. Notaulus: present, simple. Length of notaulus: approximately less than or equal to 1/3 of length of mesoscutum. Width of notaulus: narrow (notaulus width less than or equal to half the width of tegula). Sculpture of mesoscutum: weakly rugulose mixed with weak granulae. Sculpture of mesoscutellum: weakly rugulose mixed with granulate sculpture. Mesoscutellar profile: elevated, anterior margin higher than posterior. Mesoscutellar shape: flat, not depressed. Lateral propodeal area: densely, finely rugulose. Shape of propodeal anterior spine: elongate, narrow, apex rounded. Sculpture of propodeum between anterior spines: smooth or largely smooth. Sculpture of ventral half of mesepisternum: longitudinally costate on weakly coriaceous background. Sculpture of upper 1/4 of mesopleuron: densely longitudinally costate across half width. Metapleural sculpture: mainly smooth, lower third sparsely longitudinally carinate.

*Wings*. Stigmal vein: present, elongate, narrow. Campaniform sensilla at distal area of stigmal vein: present.

*Metasoma*. Shape of T1 horn: broad, short. Sculpture of upper portion of T1 horn: longitudinally carinate. Sculpture of posterior portion of T1 horn: largely smooth, with sparse longitudinal carinae. Lateral carinae on T2: present, well-defined. Sculpture of T2: largely coriaceous, smooth mesally. Sculpture of T3: smooth mesally, otherwise weakly coriaceous. Sculpture of S3–S6: S3 weakly coriaceous, S4–S6 mainly smooth with few, sparse, setigerous punctulae sculpture. S2 anterior carina: present, cristate, interrupted medially.

*Male*. Unknown.

##### Diagnosis.

The species *Odontacolus baeri* is very similar to *Odontacolus africanus* and *Odontacolus anningae* but can be distinguished from them by the absence of the S2 anterior carina. Both *Odontacolus africanus* and *Odontacolus anningae* have a clearly divided S2 anterior carina, but *Odontacolus anningae* is mainly yellow in body color, contrasting with *Odontacolus africanus* which is completely dark brown.

##### Etymology.

This species is named after the great British fossil hunter Mary Anning. The epithet is a noun in the genitive case.

##### Link to distribution map.

36

##### Material examined.

Holotype female: **CAMEROON**: Nkoemvom, 18.IX.1988, Malaise trap, D. Jackson, OSUC 238445 (deposited in BMNH).

##### Comments.

The female holotype is in perfect condition.

#### Odontacolus australiensis

Valerio & Austin sp. n.

urn:lsid:zoobank.org:act:5E7C36F8-C43C-41E8-AE10-AEDA1994DA2F

urn:lsid:biosci.ohio-state.edu:osuc_concepts:254263

http://species-id.net/wiki/Odontacolus_australiensis

Figures 25 , 61 , 89–94 , 294, 298 , 310 , 317 , 321–322 ; Morphbank ^37^

##### Description.

*Female*. Body length: 1.46 – 1.62 mm (n=5). Antenna color: completely yellow except distal half of clava slightly darker than remainder of antenna. Body color: mostly dark brown except posterior edge of metasomal terga yellowish. Coxae color: yellow. Leg color (excluding coxae): yellow. Fore wing color: completely hyaline.

*Head*. Size of compound eye: approximately 1/2× height of head. Head shape in lateral view: lower head moderately short and strongly narrowed towards mouth, head appearing short and broad. Sculpture of antennal scrobe: smooth throughout. Surface of torular triangle: flat. Development of central keel on frons: present, elongate, reaching anterior ocellus. Sculpture on upper frons below anterior ocellus: with weak rugulose-aciculate sculpture. Sculpture of malar space: with fan-like striae, striae extending into antennal scrobe. Furrow at lateral portion of antennal scrobe: absent. Shape of medial area of vertex: flat to weakly convex. Size of lateral ocelli: minute. Distance between lateral ocellus and occipital carina: greater than 1.5× maximum ocellar diameter. Lagrimal: absent or minute. Length of OOL: less than or equal to 1/3× width of ocellus. Sculpture of vertex: with rugulose sculpture mixed with weak granulae. Sculpture of occipital carina: strongly crenulate throughout. Distance from occipital carina to orbital carina: at least 2× width of occipital carina; slightly greater than width of occipital carina. Shape of occipital carina: simply arcuate medially. Sculpture of occiput: with weak, small granulae. Sculpture of gena: with weak rugulose sculpture and granulate background sculpture.

*Mesosoma*. Dorsal mesosoma in lateral view: convex. Sculpture of pronotal cervical area: with small (at most as large as crenulae on anterior edge of mesoscutum), well-defined foveae. Sculpture of pronotal lateral area: transversely costate. Netrion: absent, obscured by longitudinal sculpture of lateral pronotum. Notaulus: present, with crenulae that extend completely through depth of furrow. Length of notaulus: approximately 2/5× length of mesoscutum. Width of notaulus: narrow (notaulus width less than or equal to half the width of tegula). Sculpture of mesoscutum: finely granulose. Sculpture of mesoscutellum: granulose. Mesoscutellar profile: mainly flat, anterior and posterior edge at same height or nearly so. Mesoscutellar shape: flat, not depressed. Lateral propodeal area: sparsely transversely carinate. Shape of propodeal anterior spine: short, broad, apex rounded. Sculpture of propodeum between anterior spines: longitudinally costate. Sculpture of ventral half of mesepisternum: smooth or nearly so. Sculpture of upper 1/4 of mesopleuron: densely longitudinally costate across entire width. Metapleural sculpture: midtransverse area smooth, otherwise with cristate, longitudinal carinae.

*Wings*. Stigmal vein: present, short, broad. Campaniform sensilla at distal area of stigmal vein: present.

*Metasoma*. Shape of T1 horn: narrow, elongate. Sculpture of upper portion of T1 horn: smooth. Sculpture of posterior portion of T1 horn: longitudinally carinate. Lateral carinae on T2: absent. Sculpture of T2: meson and posterior margin smooth, otherwise longitudinally costate. Sculpture of T3: weakly coriaceous. Sculpture of S3–S6: finely, weakly coriaceous. S2 anterior carina: present, rounded, uninterrupted.

*Male*. Body length: 1. 39 mm (n=1). Body color: Antenna yellow as legs, metasoma light brown, head dark brown as mesosoma, fore wing with light infuscate color, no dark bands present. Sculpture of antennal scrobe: mesal area smooth, remainder with weakly coriaceous sculpture mixed with weak rugulose sculpture except upper 1/6 more rugulose than coriaceous. Shape and size of anterior ocellus: minute, very round. Vertex posterior area sculpture: with dense, small granulate sculpture. Occipital carina dorsal area: well-defined, conspicuously present. Netrion: practically absent by presence of longitudinal carinae on pronotal lateral areas. Sculpture of mesepisternum: mostly weakly coriaceous. Sculpture of pronotal lateral areas: with dense, fine, straight, transverse carinae. Length of fore wing stigmal vein: short. Angle of stigmal vein in relation to anterior margin of fore wing: at an angle of almost 90°. Sculpture of T2: medially smooth, sublateral areas with semi-curved longitudinal carinae.

##### Diagnosis.

This is one of two species within a group of taxa in which the netrion is obscured and its marginal crenulae are not well-defined, the mesoscutum is completely sculptured, the mesoscutellum is never with smooth areas, and has short, dense setation. *Odontacolus australiensis* can be separated from *Odontacolus gallowayi* by the deep impressed notauli that exhibit few sparse crenulae throughout their length in combination with its brown body color; *Odontacolus gallowayi* has a shallow notauli with dense crenulae throughout their length, and its body color is yellow.

##### Etymology.

This species is named after the collection locality of the species, Australia. The epithet is used as an adjective.

##### Link to distribution map.

38

##### Biology.

On tree trunk of *Eucalyptus stellulata* Sieber (Myrtales: Myrtaceae).

##### Material examined.

Holotype female: **AUSTRALIA**: ACT, Canberra, 8.VII.1966, band trap, A. Wilson, OSUC 239116 (deposited in ANIC). *Paratypes*: **AUSTRALIA**: 22 females, 4 males, OSUC 239143 (AMSA); OSUC 239118–239128, 239142 (ANIC); OSUC 239129 (BMNH); OSUC 239133–239136, 239138–239141 (CNCI); OSUC 239131 (QDPC); OSUC 148638, 239130, 239132 (WINC). *Other material*: **AUSTRALIA**: 1 female, OSUC 239117 (WINC).

##### Comments.

The holotype is in perfect condition; all paratypes are in good condition except for specimen OSUC 239143 which is missing the metasoma.

#### Odontacolus baeri

Valerio & Austin sp. n.

urn:lsid:zoobank.org:act:91C397A0-1C4B-4A32-9A55-DF5A889F3290

urn:lsid:biosci.ohio-state.edu:osuc_concepts:281689

http://species-id.net/wiki/Odontacolus_baeri

Figures 48, 52 , 70 , 95–100 ; Morphbank ^39^

##### Description.

*Female*. Body length: 1.44 mm (n=1). Antenna color: completely yellow. Body color: mainly yellow, T1 horn dark brown, anteromesal mesoscutum honey yellow. Coxae color: whitish yellow. Leg color (excluding coxae): whitish yellow. Fore wing color: slightly infuscate throughout.

*Head*. Size of compound eye: approximately 1/2× height of head. Head shape in lateral view: lower head elongate and broad at mouth, head appearing elongate and somewhat thin. Sculpture of antennal scrobe: weakly coriaceous throughout. Surface of torular triangle: slightly bulging. Development of central keel on frons: present, short (less than 1/3 of frons height). Sculpture on upper frons below anterior ocellus: granulose throughout. Sculpture of malar space: coriaceous throughout, without fan-like striae. Furrow at lateral portion of antennal scrobe: absent. Mesal surface of vertex: flat to weakly convex. Size of lateral ocelli: small. Distance between lateral ocellus and occipital carina: 0.5–1.2× maximum ocellar diameter. Lagrimal: absent or minute. Length of OOL: less than or equal to 1/3× width of ocellus. Sculpture of vertex: granulate. Sculpture of occipital carina: largely simple, at most with sparse weak crenulae medially. Distance from occipital carina to orbital carina: at least 2× width of occipital carina. Shape of occipital carina: simply arcuate medially. Sculpture of occiput: with weak, small granulae. Sculpture of gena: granulose.

*Mesosoma*. Dorsal mesosoma in lateral view: convex. Sculpture of pronotal cervical area: smooth. Sculpture of pronotal lateral area: upper 1/3 granulose, lower 1/3 with transverse foveae, otherwise smooth. Netrion: present, smooth, linear. Notaulus: present, simple. Length of notaulus: approximately less than or equal to 1/3 of length of mesoscutum. Width of notaulus: narrow (notaulus width less than or equal to half the width of tegula). Sculpture of mesoscutum: weakly rugulose mixed with weak granulae. Sculpture of mesoscutellum: granulose. Mesoscutellar profile: elevated, anterior margin higher than posterior. Mesoscutellar shape: flat, not depressed. Lateral propodeal area: densely, finely rugulose. Shape of propodeal anterior spine: elongate, narrow, apex rounded. Sculpture of propodeum between anterior spines: smooth or largely smooth. Sculpture of ventral half of mesepisternum: weakly, finely coriaceous. Sculpture of upper 1/4 of mesopleuron: densely longitudinally costate across entire width. Metapleural sculpture: smooth.

*Wings*. Stigmal vein: present, elongate, narrow. Campaniform sensilla at distal area of stigmal vein: present.

*Metasoma*. Shape of T1 horn: broad, short. Sculpture of upper portion of T1 horn: longitudinally carinate. Sculpture of posterior portion of T1 horn: largely smooth, with sparse longitudinal carinae. Lateral carinae on T2: present, poorly defined. Sculpture of T2: densely, finely, longitudinally costate, weakly coriaceous sublaterally. Sculpture of T3: anterior third weakly costate sublaterally, weakly coriaceous mesally, otherwise granulose. Sculpture of S3–S6: finely, weakly coriaceous. S2 anterior carina: absent.

*Male*. Unknown.

##### Diagnosis.

This species is very similar to *Odontacolus africanus* and *Odontacolus anningae* but can be separated from them by the absence of the S2 anterior carina. Both *Odontacolus africanus* and *Odontacolus anningae* have a clearly divided S2 anterior carina.

##### Etymology.

This species is named after the amazing German biologist Karl Ernst von Baer. The epithet is a noun in the genitive case.

##### Link to distribution map.

40

##### Material examined.

Holotype female: **AUSTRALIA:** NT, Holmes Jungle, rainforest / litter, Q.M. Berlesate No. 93, Berrimah, 12°25'S, 130°55'E, 7.VII.1979, sieved litter, G. Monteith, OSUC 238430 (deposited in QMBA).

##### Comments.

The female holotype is in perfect condition.

#### Odontacolus berryae

Valerio & Austin sp. n.

urn:lsid:zoobank.org:act:A6EA78EA-F23C-4E80-ACF7-E0AA9A79E39D

urn:lsid:biosci.ohio-state.edu:osuc_concepts:254262

http://species-id.net/wiki/Odontacolus_berryae

Figures 21 , 23–24 , 67 , 101–106 ; Morphbank ^41^

##### Description.

*Female*. Body length: 1.52 mm (n=1). Antenna color: A1–A2 honey yellow, otherwise dark brown. Body color: completely dark brown, metasoma lighter in color than head or mesosoma. Coxae color: yellow. Leg color (excluding coxae): yellow. Fore wing color: completely hyaline.

*Head*. Size of compound eye: approximately 1/2× height of head. Head shape in lateral view: lower head moderately short and strongly narrowed towards mouth, head appearing short and broad. Sculpture of antennal scrobe: smooth throughout. Surface of torular triangle: slightly bulging. Development of central keel on frons: present, elongate (equal to or greater than 1/3× height of frons), but not reaching anterior ocellus. Sculpture on upper frons below anterior ocellus: with weak rugulose-aciculate sculpture. Sculpture of malar space: with fan-like striae, striae extending into antennal scrobe. Furrow at lateral portion of antennal scrobe: absent. Mesal surface of vertex: flat to weakly convex. Size of lateral ocelli: small. Distance between lateral ocellus and occipital carina: greater than 1.5× maximum ocellar diameter. Lagrimal: absent or minute. Length of OOL: less than or equal to 1/3× width of ocellus. Sculpture of vertex: with abundant areolate sculpture mixed with dense, fine granulae. Sculpture of occipital carina: strongly crenulate throughout. Distance from occipital carina to orbital carina: at least 2× width of occipital carina. Shape of occipital carina: simply arcuate medially. Sculpture of occiput: with weakly rugulo aciculate sculpture. Sculpture of gena: with weak rugulose sculpture and granulate background sculpture.

*Mesosoma*. Dorsal mesosoma in lateral view: convex. Sculpture of pronotal cervical area: with conspicuously elongate (clearly larger than crenulae on anterior edge of mesoscutum), well-defined foveae. Sculpture of pronotal lateral area: transversely costate. Netrion: absent, obscured by longitudinal sculpture of lateral pronotum. Notaulus: present, with crenulae that extend completely through depth of furrow. Length of notaulus: approximately 2/5× length of mesoscutum. Width of notaulus: distinctly widened (notaulus width greater than half the width of tegula). Sculpture of mesoscutum: finely granulose. Sculpture of mesoscutellum: with weak, fine, granulate sculpture. Mesoscutellar profile: mainly flat, anterior and posterior edge at same height or nearly so. Mesoscutellar shape: flat, not depressed. Lateral propodeal area: longitudinally costate. Shape of propodeal anterior spine: short, broad, apex rounded. Sculpture of propodeum between anterior spines: longitudinally costate. Sculpture of ventral half of mesepisternum: longitudinally costate. Sculpture of upper 1/4 of mesopleuron: densely longitudinally costate across entire width. Metapleural sculpture: midtransverse area smooth, otherwise with cristate, longitudinal carinae.

*Wings*. Stigmal vein: present, short, narrow. Campaniform sensilla at distal area of stigmal vein: present.

*Metasoma*. Shape of T1 horn: narrow, elongate. Sculpture of upper portion of T1 horn: smooth. Sculpture of posterior portion of T1 horn: largely smooth, with sparse longitudinal carinae. Lateral carinae on T2: absent. Sculpture of T2: meson and posterior margin smooth, otherwise longitudinally costate. Sculpture of T3: smooth mesally, otherwise weakly coriaceous. Sculpture of S3–S6: finely, weakly coriaceous. S2 anterior carina: present, rounded, uninterrupted.

*Male*. Unknown.

##### Diagnosis.

This species may be distinguished from all others with an obscured netrion by the combination of the very wide notauli that have a few, broad and well-defined crenulae; the mesoscutum broadly smooth in its posterior half; and the mesoscutellum mesally with a smooth patch and its conspicuously long but somewhat sparse setae.

##### Etymology.

This species is named after our entomological colleague Dr Jo Berry from New Zealand. The epithet is a noun in the genitive case.

##### Link to distribution map.

42

##### Biology.

Recorded as an egg parasitoid of *Trite planiceps* Simon (Araneae: Salticidae) in New Zealand.

##### Material examined.

Holotype female: **NEW ZEALAND**: Gisborne Unit. Auth., Gisborne Dist., grape vines / corrugated cardboard, Manutuke, 25.I.1990, J. G. Charles, OSUC 238563 (deposited in NZAC). *Paratypes*: (12 females) **AUSTRALIA**: 2 females, OSUC 239137 (CNCI); OSUC 238564 (QDPC). **NEW ZEALAND**: 9 females, OSUC 238528, 238537, 238544 (LUNZ); OSUC 238560 (OSUC); OSUC 238509, 238545, 238549, 238580, (NZAC); 238587 (WINC). **NORFOLK ISLAND**: 1 female, OSUC 238515 (ANIC).

##### Comments.

The holotype is in perfect condition as are the remainder of the paratypes except for specimen OSUC 238564 which is covered with dust.

#### Odontacolus bosei

Valerio & Austin sp. n.

urn:lsid:zoobank.org:act:6E58CAAC-BF18-49F3-AE76-EF9EA9564E59

urn:lsid:biosci.ohio-state.edu:osuc_concepts:254277

http://species-id.net/wiki/Odontacolus_bosei

Figures 57 , 107–112 ; Morphbank ^43^

##### Description.

*Female*. Body length: 1.11 – 1.56 mm (n=20). Antenna color: clava and A2 light honey yellow, remainder of antenna yellow. Body color: completely dark brown. Coxae color: yellow. Leg color (excluding coxae): yellow. Fore wing color: slightly infuscate throughout.

*Head*. Size of compound eye: approximately 1/2× height of head. Head shape in lateral view: lower head elongate and broad at mouth, head appearing elongate and somewhat thin. Sculpture of antennal scrobe: largely smooth ventrally, dorsally with sinuate, transverse ridges. Surface of torular triangle: slightly bulging. Development of central keel on frons: present, elongate, reaching anterior ocellus. Sculpture on upper frons below anterior ocellus: with sparse, transverse costae mixed with weak, dense granulae. Sculpture of malar space: with weak rugulose sculpture mixed with granulate sculpture. Furrow at lateral portion of antennal scrobe: absent. Mesal surface of vertex: flat to weakly convex. Size of lateral ocelli: normal. Distance between lateral ocellus and occipital carina: 0.5–1.2× maximum ocellar diameter. Lagrimal: absent or minute. Length of OOL: less than or equal to 1/3× width of ocellus. Sculpture of vertex: with rugulose sculpture mixed with weak granulae. Sculpture of occipital carina: weakly crenulate throughout. Distance from occipital carina to orbital carina: at least 2× width of occipital carina. Shape of occipital carina: simply arcuate medially. Sculpture of occiput: with weakly rugulo aciculate sculpture. Sculpture of gena: with weak rugulose sculpture and granulate background sculpture.

*Mesosoma*. Dorsal mesosoma in lateral view: convex. Sculpture of pronotal cervical area: with small (at most as large as crenulae on anterior edge of mesoscutum), well-defined foveae. Sculpture of pronotal lateral area: upper 1/3 granulose, lower 1/3 with transverse foveae, otherwise smooth. Netrion: present, smooth, well developed, sub-obovate. Notaulus: present, simple. Length of notaulus: approximately less than or equal to 1/3 of length of mesoscutum. Width of notaulus: narrow (notaulus width less than or equal to half the width of tegula). Sculpture of mesoscutum: weakly rugulose mixed with weak granulae. Sculpture of mesoscutellum: granulose. Mesoscutellar profile: elevated, anterior margin higher than posterior. Mesoscutellar shape: flat, not depressed. Lateral propodeal area: densely transversely carinate. Shape of propodeal anterior spine: elongate, narrow, apex rounded. Sculpture of propodeum between anterior spines: smooth or largely smooth. Sculpture of ventral half of mesepisternum: smooth or nearly so. Sculpture of upper 1/4 of mesopleuron: densely longitudinally costate across half width. Metapleural sculpture: largely smooth, longitudinally carinate ventrally; mainly smooth, lower third sparsely longitudinally carinate.

*Wings*. Stigmal vein: present, elongate, narrow. Campaniform sensilla at distal area of stigmal vein: present.

*Metasoma*. Shape of T1 horn: narrow, short. Sculpture of upper portion of T1 horn: longitudinally carinate. Sculpture of posterior portion of T1 horn: largely smooth, with sparse longitudinal carinae. Lateral carinae on T2: present, well-defined. Sculpture of T2: largely weakly coriaceous mixed with longitudinal costae, meson coriaceous. Sculpture of T3: anterior half coriaceous mesally, otherwise granulate. Sculpture of S3–S6: finely, weakly coriaceous. S2 anterior carina: present, cristate, uninterrupted.

*Male*. Unknown.

##### Diagnosis.

This species is very similar to *Odontacolus markadicus*, but can be distinguished by the completely dark brown body and the conspicuously crenulate lateral occipital carina. In *Odontacolus markadicus* the body color is yellow and the lateral occipital carina is mainly smooth.

##### Etymology.

This species is name after the amazing Bengali painter Nandalal Bose, recipient of the “Padma Vibhushan”. The epithet is a noun in the genitive case.

##### Link to distribution map.

44

##### Material examined.

Holotype female: **INDIA**: Karnataka St., Bangalore, 1.VII-10.VII.1988, pan trap, K. Ghorpade, OSUC 239193 (deposited in CNCI). *Paratypes*: (33 females) **INDIA**: 31 females, OSUC 238773-238779, 238784-238792, 238794-238802, 238807-238808, 239194 (CNCI); OSUC 238780-238781, 238783 (OSUC). **MALAYSIA**: 1 female, OSUC 239158 (CNCI). **SRI LANKA**: 1 female, OSUC 239204 (CNCI).

##### Comments.

The holotype is in perfect condition as are most of the paratypes except for specimen OSUC 238789 which has the left fore wing glued to the point, and OSUC 238790 which has the metascutellum detached from the body.

#### Odontacolus cardaleae

Valerio & Austin sp. n.

urn:lsid:zoobank.org:act:A0F1D450-8E8B-46D8-B941-96FEFE39981F

urn:lsid:biosci.ohio-state.edu:osuc_concepts:254270

http://species-id.net/wiki/Odontacolus_cardaleae

Figures 39 , 113–118 ; Morphbank ^45^

##### Description.

*Female*. Body length: 1.65 mm (n=1). Antenna color: completely yellow. Body color: mostly yellow, propodeum, propodeal anterior spines, T1 horn dark brown. Coxae color: yellow. Leg color (excluding coxae): yellow. Fore wing color: completely hyaline.

*Head*. Size of compound eye: approximately 1/2× height of head. Head shape in lateral view: lower head elongate and broad at mouth, head appearing elongate and somewhat thin. Sculpture of antennal scrobe: with weak, sinuate, transverse ridges throughout. Surface of torular triangle: slightly bulging. Development of central keel on frons: present, elongate (equal to or greater than 1/3× height of frons), but not reaching anterior ocellus. Sculpture of vertex: with abundant areolate sculpture mixed with dense, fine granulae sculpture. Sculpture on upper frons below anterior ocellus: covered by sinuate, transverse, fine costae. Sculpture of malar space: granulose throughout, without fan-like striae. Furrow at lateral portion of antennal scrobe: absent. Mesal surface of vertex: flat to weakly convex. Size of lateral ocelli: normal. Distance between lateral ocellus and occipital carina: 0.5–1.2× maximum ocellar diameter. Lagrimal: absent or minute. Length of OOL: less than or equal to 1/3× width of ocellus. Sculpture of occipital carina: largely simple, at most with sparse weak crenulae medially. Distance from occipital carina to orbital carina: at least 2× width of occipital carina. Shape of occipital carina: simply arcuate medially. Sculpture of occiput: with weakly rugulo aciculate sculpture. Sculpture of gena: granulose.

*Mesosoma*. Dorsal mesosoma in lateral view: convex. Sculpture of pronotal cervical area: with small (at most as large as crenulae on anterior edge of mesoscutum), well-defined foveae. Sculpture of pronotal lateral area: largely smooth, dorsal margin with dense, weak punctulae. Netrion: present, smooth, linear. Notaulus: present, with crenulae that extend completely through depth of furrow. Length of notaulus: approximately less than or equal to 1/3 of length of mesoscutum. Width of notaulus: narrow (notaulus width less than or equal to half the width of tegula). Sculpture of mesoscutum: finely granulose. Sculpture of mesoscutellum: with weak, fine, granulate sculpture. Mesoscutellar profile: mainly flat, anterior and posterior edge at same height or nearly so. Mesoscutellar shape: flat, not depressed. Lateral propodeal area: densely, finely rugulose. Shape of propodeal anterior spine: elongate, narrow, apex rounded. Sculpture of propodeum between anterior spines: rugulose throughout. Sculpture of ventral half of mesepisternum: weakly coriaceous. Sculpture of upper 1/4 of mesopleuron: densely longitudinally costate across entire width. Metapleural sculpture: mainly with weak coriaceous sculpture, lower 1/3 without longitudinal costae.

*Wings*. Stigmal vein: present, elongate, narrow. Campaniform sensilla at distal area of stigmal vein: present.

*Metasoma*. Shape of T1 horn: broad, short. Sculpture of upper portion of T1 horn: longitudinally carinate. Sculpture of posterior portion of T1 horn: transversely carinate. Lateral carinae on T2: present, well-defined. Sculpture of T2: longitudinally costate on coriaceous background. Sculpture of T3: weakly coriaceous. Sculpture of S3–S6: finely, weakly coriaceous. S2 anterior carina: present, cristate, uninterrupted.

*Male*. Unknown.

##### Diagnosis.

The species *Odontacolus cardaleae* belongs to a group having the occipital carina separated from the orbital carina, a well-defined netrion, a central keel on the frons, and well-defined notauli. However, *Odontacolus cardaleae* is the only species in the group that has a few broad crenulae across the notauli and the antennal scrobes with transverse sinuate ridges. *Odontacolus heydoni* can be separated from *Odontacolus cardaleae* by its large ocelli (Fig. 40) and the short distance between the lateral ocellus and occipital carina (approximately 0.5× the ocellar diameter); *Odontacolus cardaleae* has small ocelli and the distance between lateral ocellus and occipital carina is approximately 1.5× the ocellar diameter.

##### Etymology.

This species is named after Josephine Cardale, former collection manager of Hymenoptera at the ANIC, who collected this species. The epithet is a noun in the genitive case.

##### Link to distribution map.

46

##### Material examined.

Holotype female: **AUSTRALIA**: QLD, Shiptons Flat, 15°47'S, 145°14'E, 17.X–19.X.1980, Malaise trap, J. C. Cardale, OSUC 237936 (deposited in ANIC).

##### Comments.

The holotype is in perfect condition.

#### Odontacolus darwini

Valerio & Austin sp. n.

urn:lsid:zoobank.org:act:07961E9D-C67B-415A-A854-7A70FC9B8F9F

urn:lsid:biosci.ohio-state.edu:osuc_concepts:254272

http://species-id.net/wiki/Odontacolus_darwini

Figures 63 , 119–124 ; Morphbank ^47^

##### Description.

*Female*. Body length: 1.22 – 1.78 mm (n=5). Antenna color: A1 yellow, otherwise dark brown. Body color: head dark brown, mesosoma, T1 (except dorsal and posterior area of T1 horn dark brown), anterior 1/3 of T2 light orange, remainder of metasoma dark brown. Coxae color: yellow. Leg color (excluding coxae): yellow. Fore wing color: completely hyaline.

*Head*. Size of compound eye: approximately 1/2× height of head. Head shape in lateral view: lower head elongate and broad at mouth, head appearing elongate and somewhat thin.

Sculpture of antennal scrobe: largely smooth ventrally, dorsally with sinuate, transverse ridges. Surface of torular triangle: flat. Development of central keel on frons: present, elongate (equal to or greater than 1/3× height of frons), but not reaching anterior ocellus; present, elongate, reaching anterior ocellus. Sculpture on upper frons below anterior ocellus: with sparse, transverse costae mixed with weak, dense granulae. Sculpture of malar space: with sparse, fine, dorsoventral carinae. Furrow at lateral portion of antennal scrobe: absent. Mesal surface of vertex: flat to weakly convex. Size of lateral ocelli: large. Distance between lateral ocellus and occipital carina: 0.5–1.2× maximum ocellar diameter. Lagrimal: absent or minute. Length of OOL: less than or equal to 1/3× width of ocellus. Sculpture of vertex: granulate. Sculpture of occipital carina: weakly crenulate throughout. Distance from occipital carina to orbital carina: at least 2× width of occipital carina. Shape of occipital carina: simply arcuate medially. Sculpture of occiput: with weak, small granulae. Sculpture of gena: granulose.

*Mesosoma*. Dorsal mesosoma in lateral view: convex. Sculpture of pronotal cervical area: with small (at most as large as crenulae on anterior edge of mesoscutum), well-defined foveae. Sculpture of pronotal lateral area: coriaceous. Netrion: present, smooth, well developed, not looking - linear. Notaulus: present, simple. Length of notaulus: approximately less than or equal to 1/3 of length of mesoscutum. Width of notaulus: narrow (notaulus width less than or equal to half the width of tegula). Sculpture of mesoscutum: densely, finely granulate, posterior 1/4 more coarsely coriaceous. Sculpture of mesoscutellum: with weak, fine, granulate sculpture. Mesoscutellar profile: elevated, anterior margin higher than posterior. Mesoscutellar shape: flat, not depressed. Lateral propodeal area: sparsely transversely carinate. Shape of propodeal anterior spine: short, broad, apex subtriangular. Sculpture of propodeum between anterior spines: smooth or largely smooth. Sculpture of ventral half of mesepisternum: smooth or nearly so. Sculpture of upper 1/4 of mesopleuron: densely longitudinally costate across half width. Metapleural sculpture: sparsely longitudinally costate.

*Wings*. Stigmal vein: present, elongate, narrow. Campaniform sensilla at distal area of stigmal vein: present.

*Metasoma*. Shape of T1 horn: broad, short. Sculpture of upper portion of T1 horn: longitudinally carinate. Sculpture of posterior portion of T1 horn: largely smooth, with sparse longitudinal carinae. Lateral carinae on T2: present, well-defined. Sculpture of T2: longitudinally costate on coriaceous background. Sculpture of T3: anterior third weakly costate sublaterally, weakly coriaceous mesally, otherwise granulose. Sculpture of S3–S6: finely, weakly coriaceous. S2 anterior carina: present, cristate, uninterrupted.

*Male*. Unknown.

##### Diagnosis.

This species is easily identified based on its unique color pattern; there is no other known species that has the head and metasoma black in combination with an orange mesosoma and yellow legs.

##### Etymology.

This species is named after the famed British biologist Charles Robert Darwin. The epithet is a noun in the genitive case.

##### Link to distribution map.

48

##### Material examined.

Holotype female: **THAILAND**: Nakhon Nayok Prov., behind vegetable garden, T149, Khao Yai National Park, 14°24.761'N, 101°22.815'E, 19.VII–26.VII.2006, Malaise trap, P. Sandao, OSUC 233092 (deposited in QSBG). *Paratypes*: (13 females) **MALAYSIA**: 3 females, OSUC 239206–239207 (CNCI); OSUC 239208 (WINC). **THAILAND**: 10 females, OSUC 247787, 339582, 339603 (OSUC); OSUC 250602, 339576, 339581, 339583, 339589 (QSBG); UCRC ENT 135102, 135138 (UCRC).

##### Comments.

The holotype is in perfect condition. Paratype OSUC 250602 is smaller in size than other specimens, and also has the mesal area of T2 smooth, the anterior mesal area of T3 mainly smooth with the exception of some weak, sparse longitudinal costae contrasting with the rest of specimens in which the sculpturing of T3 is as follows: anterior third is weakly costate sublaterally, weakly coriaceous mesally but otherwise granulose. Specimen OSUC 339589 has the metasoma lighter in color than other specimens.

#### Odontacolus dayi

Valerio & Austin sp. n.

urn:lsid:zoobank.org:act:A1879B39-5334-4E71-A5E1-5A0082DCD8BD

urn:lsid:biosci.ohio-state.edu:osuc_concepts:254328

http://species-id.net/wiki/Odontacolus_dayi

Figures 43 , 125–130 ; Morphbank ^49^

##### Description.

*Female*. Body length: 1.68 mm (n=1). Antenna color: clava and A2 light honey yellow, remainder of antenna yellow. Body color: head and metasoma beyond T1 dark brown, mesosoma and T1 honey yellow. Coxae color: yellow. Leg color (excluding coxae): yellow. Fore wing color: slightly infuscate throughout.

*Head*. Size of compound eye: approximately 1/2× height of head. Head shape in lateral view: lower head elongate and broad at mouth, head appearing elongate and somewhat thin. Sculpture of antennal scrobe: largely smooth ventrally, sparsely granulate ventrally. Surface of torular triangle: flat. Development of central keel on frons: present, elongate (equal to or greater than 1/3× height of frons), but not reaching anterior ocellus. Sculpture on upper frons below anterior ocellus: granulose throughout. Sculpture of malar space: with sparse, short fan-like striae, striae not extending into scrobal area, mixed with weak coriaceous sculpture. Furrow at lateral portion of antennal scrobe: absent. Mesal surface of vertex: slightly depressed. Size of lateral ocelli: large. Distance between lateral ocellus and occipital carina: 0.5–1.2× maximum ocellar diameter. Lagrimal: absent or minute. Length of OOL: less than or equal to 1/3× width of ocellus. Sculpture of vertex: granulate. Sculpture of occipital carina: largely simple, at most with sparse weak crenulae medially. Distance from occipital carina to orbital carina: at least 2× width of occipital carina. Shape of occipital carina: simply arcuate medially. Sculpture of occiput: with weak, small granulae. Sculpture of gena: granulose.

*Mesosoma*. Dorsal mesosoma in lateral view: convex. Sculpture of pronotal cervical area: with small (at most as large as crenulae on anterior edge of mesoscutum), well-defined foveae. Sculpture of pronotal lateral area: coriaceous. Netrion: present, smooth, well developed, sub-obovate. Notaulus: present, simple. Length of notaulus: approximately less than or equal to 1/3 of length of mesoscutum. Width of notaulus: narrow (notaulus width less than or equal to half the width of tegula). Sculpture of mesoscutum: weakly rugulose mixed with weak granulae. Sculpture of mesoscutellum: granulose. Mesoscutellar profile: elevated, anterior margin higher than posterior. Mesoscutellar shape: flat, not depressed. Lateral propodeal area: coarsely rugulose. Shape of propodeal anterior spine: elongate, narrow, apex rounded. Sculpture of propodeum between anterior spines: smooth or largely smooth. Sculpture of ventral half of mesepisternum: smooth or nearly so. Sculpture of upper 1/4 of mesopleuron: densely longitudinally costate across half width. Metapleural sculpture: largely smooth except lower half with longitudinal carinae.

*Wings*. Stigmal vein: present, elongate, narrow. Campaniform sensilla at distal area of stigmal vein: present.

*Metasoma*. Shape of T1 horn: narrow, short. Sculpture of upper portion of T1 horn: longitudinally carinate. Sculpture of posterior portion of T1 horn: largely smooth, with sparse longitudinal carinae. Lateral carinae on T2: present, well-defined. Sculpture of T2: largely weakly coriaceous mixed with longitudinal costae, meson coriaceous. Sculpture of T3: anterior half weakly, longitudinally costate, coriaceous mesally, otherwise weakly coriaceous. Sculpture of S3–S6: finely, weakly coriaceous. S2 anterior carina: present, cristate, uninterrupted.

*Male*. Unknown.

##### Diagnosis.

This is the only known species that has a longitudinally depressed vertex. In the Neotropics there is one undescribed species (in CNCI) with a very conspicuous longitudinal depression on the vertex, but the general sculpture of the body is very different from *Odontacolus dayi*.

##### Etymology.

This species is named after the hymenopterist M. C. Day, now retired from the Natural History Museum, London. The epithet is a noun in the genitive case.

##### Link to distribution map.

50

##### Material examined.

Holotype female: **INDONESIA**: Maluku Prov., Ceram (Seram) Isl., Solea, VIII–1987, M. C. Day, OSUC 238418 (deposited in BMNH).

##### Comments.

The holotype is in perfect condition.

#### Odontacolus gallowayi

Valerio & Austin sp. n.

urn:lsid:zoobank.org:act:E956719F-F685-4677-AEEF-F8A6E20F9B76

urn:lsid:biosci.ohio-state.edu:osuc_concepts:254260

http://species-id.net/wiki/Odontacolus_gallowayi

Figures 12 , 137–142 ; Morphbank ^51^

##### Description.

*Female*. Body length: 1.49mm (n=2). Antenna color: completely yellow. Body color: completely yellow. Coxae color: yellow. Leg color (excluding coxae): yellow. Fore wing color: completely hyaline.

*Head*. Size of compound eye: approximately 1/2× height of head. Head shape in lateral view: lower head moderately short and strongly narrowed towards mouth, head appearing short and broad. Sculpture of antennal scrobe: smooth throughout. Surface of torular triangle: slightly bulging. Development of central keel on frons: present, elongate, reaching anterior ocellus. Sculpture on upper frons below anterior ocellus: with weak rugulose-aciculate sculpture. Sculpture of malar space: with fan-like striae, striae extending into antennal scrobe. Furrow at lateral portion of antennal scrobe: absent. Mesal surface of vertex: flat to weakly convex. Size of lateral ocelli: small. Distance between lateral ocellus and occipital carina: greater than 1.5× maximum ocellar diameter. Lagrimal: absent or minute. Length of OOL: less than or equal to 1/3× width of ocellus. Sculpture of vertex: with abundant areolate sculpture mixed with dense, fine granulae. Sculpture of occipital carina: weakly crenulate throughout. Distance from occipital carina to orbital carina: at least 2× width of occipital carina. Shape of occipital carina: weakly sinuate medially. Sculpture of occiput: with weakly rugulo aciculate sculpture. Sculpture of gena: with weak rugulose sculpture and granulate background sculpture.

*Mesosoma*. Dorsal mesosoma in lateral view: convex. Sculpture of pronotal cervical area: with small (at most as large as crenulae on anterior edge of mesoscutum), well-defined foveae. Sculpture of pronotal lateral area: largely covered by longitudinal costae except upper 1/5 foveate. Netrion: absent, obscured by longitudinal sculpture of lateral pronotum. Notaulus: present, with low crenulae that do not extend through depth of furrow. Length of notaulus: approximately less than or equal to 1/3 of length of mesoscutum. Width of notaulus: narrow (notaulus width less than or equal to half the width of tegula). Sculpture of mesoscutum: finely granulose. Sculpture of mesoscutellum: with weak, fine, granulate sculpture. Mesoscutellar profile: elevated, anterior margin higher than posterior. Mesoscutellar shape: flat, not depressed. Lateral propodeal area: sparsely transversely carinate. Shape of propodeal anterior spine: short, broad, apex rounded. Sculpture of propodeum between anterior spines: smooth or largely smooth. Sculpture of ventral half of mesepisternum: smooth or nearly so. Sculpture of upper 1/4 of mesopleuron: sparsely longitudinally costate across entire width. Metapleural sculpture: smooth.

*Wings*. Stigmal vein: present, short, narrow. Campaniform sensilla at distal area of stigmal vein: present.

*Metasoma*. Shape of T1 horn: narrow, elongate. Sculpture of upper portion of T1 horn: smooth. Sculpture of posterior portion of T1 horn: longitudinally carinate. Lateral carinae on T2: absent. Sculpture of T2: largely longitudinally costate, meson smooth, weakly granulate laterally. Sculpture of T3: weakly coriaceous. Sculpture of S3–S6: finely, weakly coriaceous. S2 anterior carina: present, rounded, uninterrupted.

*Male*. Unknown.

##### Diagnosis.

This is one of two species within a group of taxa that has an obscured netrion, notaular crenulae poorly defined, and the mesoscutum completely sculptured (without smooth areas) and entirely covered by short, abundant setae. *Odontacolus australiensis* can be separated from *Odontacolus gallowayi* by the deep impressed notauli that exhibit few sparse crenulae throughout their length in combination with its brown body color; *Odontacolus gallowayi* has a shallow notauli with dense crenulae throughout its length, and its body color is yellow.

##### Etymology.

This species is named after the Australian hymenopterist I. D. Galloway. The epithet is a noun in the genitive case

##### Link to distribution map.

52

##### Material examined.

Holotype female: **AUSTRALIA**: QLD, via Julatten, Mount Lewis, 28.III.1976, I. D. Galloway, OSUC 238000 (deposited in QMBA). *Paratype*: **AUSTRALIA**: 1 female, OSUC 237999 (QDPC).

##### Comments.

The holotype is in perfect condition; the paratype has the right hind wing and metasoma detached but glued to a card point.

#### Odontacolus gentingensis

Valerio & Austin sp. n.

urn:lsid:zoobank.org:act:3ACBDD21-9C7B-4537-A2B8-875F2578D3AA

urn:lsid:biosci.ohio-state.edu:osuc_concepts:254259

http://species-id.net/wiki/Odontacolus_gentingensis

Figures 36 , 143–148 ; Morphbank ^53^

##### Description.

*Female*. Body length: 1.88 mm (n=1). Antenna color: A1 yellow, otherwise dark brown. Body color: head yellow, mesosoma mostly yellow, mesoscutum anteromesally and posterolaterally dark honey yellow, metasoma mainly dark honey yellow, sublateral areas of T3 yellow. Coxae color: yellow. Leg color (excluding coxae): yellow. Fore wing color: completely hyaline.

*Head*. Size of compound eye: approximately 1/2× height of head. Head shape in lateral view: lower head elongate and broad at mouth, head appearing elongate and somewhat thin. Sculpture of antennal scrobe: smooth throughout. Surface of torular triangle: flat. Development of central keel on frons: present, elongate (equal to or greater than 1/3× height of frons), but not reaching anterior ocellus. Sculpture on upper frons below anterior ocellus: granulose throughout. Sculpture of malar space: with fan-like striae, striae extending into antennal scrobe. Furrow at lateral portion of antennal scrobe: absent. Mesal surface of vertex: flat to weakly convex. Size of lateral ocelli: large. Distance between lateral ocellus and occipital carina: less than 0.5× maximum ocellar diameter. Lagrimal: conspicuously present (approximately 0.4× of malar sulcus length). Length of OOL: less than or equal to 1/3× width of ocellus. Sculpture of vertex: granulate. Sculpture of occipital carina: largely simple, at most with sparse weak crenulae medially. Distance from occipital carina to orbital carina: at least 2× width of occipital carina. Shape of occipital carina: simply arcuate medially. Sculpture of occiput: with weakly rugulo aciculate sculpture. Sculpture of gena: granulose.

*Mesosoma*. Dorsal mesosoma in lateral view: convex. Sculpture of pronotal cervical area: with small (at most as large as crenulae on anterior edge of mesoscutum), well-defined foveae. Sculpture of pronotal lateral area: smooth or nearly so. Netrion: present, smooth, linear. Notaulus: present, simple. Length of notaulus: approximately less than or equal to 1/3 of length of mesoscutum. Width of notaulus: narrow (notaulus width less than or equal to half the width of tegula). Sculpture of mesoscutum: anterior half coriaceous, otherwise densely granulose. Sculpture of mesoscutellum: granulose. Mesoscutellar profile: mainly flat, anterior and posterior edge at same height or nearly so. Mesoscutellar shape: flat, not depressed. Lateral propodeal area: longitudinally costate. Shape of propodeal anterior spine: elongate, narrow, apex rounded. Sculpture of propodeum between anterior spines: transversely costate. Sculpture of ventral half of mesepisternum: weakly coriaceous. Sculpture of upper 1/4 of mesopleuron: densely longitudinally costate across half width. Metapleural sculpture: mainly with weak coriaceous sculpture, lower 1/3 with sparse longitudinal carinae.

*Wings*. Stigmal vein: present, elongate, narrow. Campaniform sensilla at distal area of stigmal vein: present.

*Metasoma*. Shape of T1 horn: narrow, elongate. Sculpture of upper portion of T1 horn: longitudinally carinate. Sculpture of posterior portion of T1 horn: longitudinally carinate. Lateral carinae on T2: present, poorly defined. Sculpture of T2: longitudinally costate, posterior margin smooth. Sculpture of T3: anterior third weakly longitudinally costate, otherwise coriaceous. Sculpture of S3–S6: finely, weakly coriaceous. S2 anterior carina: present, cristate, uninterrupted.

*Male*. Unknown.

##### Diagnosis.

This species can be easily distinguished from all other *Odontacolus* species based on the large and conspicuous lagrimal in combination with the occipital carina which is almost touching the orbital carina.

##### Etymology.

This species is named after the Genting Tea Estate, Malaysia from where the species was collected. The epithet is used as an adjective.

##### Link to distribution map.

54

##### Material examined.

Holotype female: **MALAYSIA**: Pahang St., Genting Tea Estate, 2000ft, VII–1985–VIII–1985, Malaise trap, W. Budenberg, OSUC 237935 (deposited in CNCI).

##### Comments.

The holotype is in perfect condition.

#### Odontacolus guineensis

Valerio & Austin sp. n.

urn:lsid:zoobank.org:act:29CCAAFE-F71F-44F3-89A5-D35DD067BA57

urn:lsid:biosci.ohio-state.edu:osuc_concepts:254257

http://species-id.net/wiki/Odontacolus_guineensis

Figures 33–34 , 149–154 ; Morphbank ^55^

##### Description.

*Female*. Body length: 2.38 mm (n=1). Antenna color: A1 yellow, otherwise dark brown. Body color: completely dark brown. Coxae color: yellow. Leg color (excluding coxae): yellow. Fore wing color: slightly infuscate throughout.

*Head*. Size of compound eye: approximately 1/2× height of head. Head shape in lateral view: lower head elongate and broad at mouth, head appearing elongate and somewhat thin. Sculpture of antennal scrobe: with weak, sinuate, transverse ridges throughout. Surface of torular triangle: flat. Development of central keel on frons: present, elongate (equal to or greater than 1/3× height of frons), but not reaching anterior ocellus. Sculpture on upper frons below anterior ocellus: with weak rugulose-aciculate sculpture. Sculpture of malar space: weakly rugulose throughout, without fan-like striae. Furrow at lateral portion of antennal scrobe: absent. Mesal surface of vertex: flat to weakly convex. Size of lateral ocelli: large. Distance between lateral ocellus and occipital carina: 0.5–1.2× maximum ocellar diameter. Lagrimal: absent or minute. Length of OOL: less than or equal to 1/3× width of ocellus. Sculpture of vertex: with abundant areolate sculpture mixed with dense, fine granulae. Sculpture of occipital carina: weakly crenulate throughout. Distance from occipital carina to orbital carina: at least 2× width of occipital carina. Shape of occipital carina: simply arcuate medially. Sculpture of occiput: with weakly rugulo aciculate sculpture. Sculpture of gena: with weak rugulose sculpture and granulate background sculpture.

*Mesosoma*. Dorsal mesosoma in lateral view: convex. Sculpture of pronotal cervical area: with small (at most as large as crenulae on anterior edge of mesoscutum), well-defined foveae. Sculpture of pronotal lateral area: weakly punctate, smooth posteroventrally. Netrion: present, smooth, linear. Notaulus: present, simple. Length of notaulus: approximately less than or equal to 1/3 of length of mesoscutum. Width of notaulus: narrow (notaulus width less than or equal to half the width of tegula). Sculpture of mesoscutum: weakly rugulose mixed with weak granulae. Sculpture of mesoscutellum: granulose. Mesoscutellar profile: mainly flat, anterior and posterior edge at same height or nearly so. Mesoscutellar shape: depressed. Lateral propodeal area: coarsely rugulose. Shape of propodeal anterior spine: elongate, narrow, apex sharply acute. Sculpture of propodeum between anterior spines: longitudinally costate. Sculpture of ventral half of mesepisternum: longitudinally costate on weakly coriaceous background. Sculpture of upper 1/4 of mesopleuron: sparsely longitudinally costate across entire width. Metapleural sculpture: mainly with weak coriaceous sculpture, lower 1/3 without longitudinal costae.

*Wings*. Stigmal vein: present, elongate, narrow. Campaniform sensilla at distal area of stigmal vein: present.

*Metasoma*. Shape of T1 horn: narrow, elongate. Sculpture of upper portion of T1 horn: longitudinally carinate. Sculpture of posterior portion of T1 horn: longitudinally carinate. Lateral carinae on T2: present, poorly defined. Sculpture of T2: longitudinally costate, posterior margin smooth. Sculpture of T3: anterior two-thirds longitudinally costate, otherwise coriaceous. Sculpture of S3–S6: weakly, longitudinally costate mixed with setigerous punctulae. S2 anterior carina: present, cristate, interrupted medially.

*Male*. Unknown.

##### Diagnosis.

This species can be distinguished from all other *Odontacolus* in which the netrion is present and the occipital carina almost touches the orbital carina by the interrupted S2 anterior carina, and the depressed posteromesal area of the mesoscutellum.

##### Etymology.

This species is named after the African country from which it was collected. The epithet is used as an adjective.

##### Link to distribution map.

56

##### Material examined.

Holotype female: **GUINEA**: Lola Pref., rainforest, Mount Nimba, 07°41 – 42'N, 08°23'W, 514–740m, XII–1990–III–1991, flight intercept trap, L. Leblanc, OSUC 237933 (deposited in CNCI).

##### Comments.

The holotype is in perfect condition.

#### Odontacolus hackeri

(Dodd)

urn:lsid:zoobank.org:act:4FED7ED3-8261-4A5F-A3E6-BE95A8419ADE

urn:lsid:biosci.ohio-state.edu:osuc_concepts:4947

http://species-id.net/wiki/Odontacolus_hackeri

Figures 155–160 ; Morphbank ^57^

Ceratobaeoides hackeri Dodd 1913: 337 (original description); Dodd 1914a: 66 (description); Kieffer 1926: 273 (description, keyed).
Odontacolus hackeri (Dodd): Austin 1981: 89 (generic transfer, type information).

##### Description.

*Female*. Body length: 1.75 mm (n=1). Antenna color: completely dark brown. Body color: head and metasoma honey yellow except for T1 horn which is darker than remainder of metasoma color, mesosoma yellow. Coxae color: yellow. Leg color (excluding coxae): yellow. Fore wing color: slightly infuscate throughout.

*Head*. Size of compound eye: approximately 1/2× height of head. Head shape in lateral view: lower head moderately short and strongly narrowed towards mouth, head appearing short and broad. Sculpture of antennal scrobe: weakly rugulose throughout. Surface of torular triangle: flat. Development of central keel on frons: present, short (less than 1/3 of frons height). Sculpture on upper frons below anterior ocellus: coriaceous throughout. Sculpture of malar space: weakly rugulose throughout, without fan-like striae. Furrow at lateral portion of antennal scrobe: absent. Mesal surface of vertex: flat to weakly convex. Size of lateral ocelli: normal. Lagrimal: absent or minute. Distance from occipital carina to orbital carina: slightly greater than width of occipital carina. Shape of occipital carina: simply arcuate medially. Sculpture of gena: granulose.

*Mesosoma*. Sculpture of pronotal lateral area: upper 1/3 granulose, lower 1/3 with transverse foveae, otherwise smooth. Netrion: present, smooth, linear. Notaulus: present, simple. Length of notaulus: approximately less than or equal to 1/3 of length of mesoscutum. Width of notaulus: narrow (notaulus width less than or equal to half the width of tegula). Sculpture of mesoscutum: weakly rugulose mixed with weak granulae. Sculpture of mesoscutellum: weakly rugulose mixed with granulate sculpture. Mesoscutellar shape: flat, not depressed. Lateral propodeal area: sparsely transversely carinate. Shape of propodeal anterior spine: short, broad, apex rounded. Sculpture of ventral half of mesepisternum: smooth or nearly so. Sculpture of upper 1/4 of mesopleuron: densely longitudinally costate across half width. Metapleural sculpture: smooth.

*Wings*. Stigmal vein: present, elongate, narrow. Campaniform sensilla at distal area of stigmal vein: present.

*Metasoma*. Shape of T1 horn: broad, short. Sculpture of upper portion of T1 horn: longitudinally carinate. Sculpture of posterior portion of T1 horn: transversely carinate. Lateral carinae on T2: present, well-defined. S2 anterior carina: present, cristate, interrupted medially.

*Male*. Unknown.

##### Diagnosis.

*Odontacolus hackeri* can be distinguished from all other Australian *Odontacolus* species by the short, smooth notauli; a well-defined netrion; a central keel being present, sculptured antennal scrobe; a short, broad T1 horn which has conspicuously transverse carinae on its posterior face; and by the flat vertex.

##### Link to distribution map.

58

##### Material examined.

Holotype female, *Ceratobaeoides hackeri*: **AUSTRALIA**: QLD, among undergrowth, Brisbane, 26.IV.1913, H. Hacker, QMBA HY1630 (deposited in QMBA).

##### Comments.

The holotype is slide mounted and partly destroyed (Figs 155–160).

#### Odontacolus harveyi

Valerio & Austin sp. n.

urn:lsid:zoobank.org:act:AC5B30E0-0542-45AB-A6E1-13A378F1C1BA

urn:lsid:biosci.ohio-state.edu:osuc_concepts:254264

http://species-id.net/wiki/Odontacolus_harveyi

Figures 17 , 29 , 153 –158 ; Morphbank ^59^

##### Description.

*Female*. Body length: 1.50 mm (n=1). Antenna color: A1–A5 yellow, otherwise dark brown. Body color: head and body honey yellow, metasoma mainly dark honey yellow, T1 (except horn) and most of T3 yellow. Coxae color: honey yellow. Leg color (excluding coxae): honey yellow. Fore wing color: completely hyaline.

*Head*. Size of compound eye: approximately 1/2× height of head. Head shape in lateral view: lower head moderately short and strongly narrowed towards mouth, head appearing short and broad. Sculpture of antennal scrobe: smooth throughout. Surface of torular triangle: slightly bulging. Development of central keel on frons: present, elongate, reaching anterior ocellus. Sculpture on upper frons below anterior ocellus: covered by sinuate, transverse, fine costae. Sculpture of malar space: with fan-like striae, striae not extending into antennal scrobe. Furrow at lateral portion of antennal scrobe: absent. Mesal surface of vertex: flat to weakly convex. Size of lateral ocelli: minute. Distance between lateral ocellus and occipital carina: greater than 1.5× maximum ocellar diameter. Lagrimal: absent or minute. Length of OOL: less than or equal to 1/3× width of ocellus. Sculpture of vertex: with sparse punctate sculpture mixed with dense, fine granulae. Sculpture of occipital carina: largely simple, at most with sparse weak crenulae medially. Distance from occipital carina to orbital carina: at least 2× width of occipital carina. Shape of occipital carina: weakly sinuate medially. Sculpture of occiput: with weakly rugulo aciculate sculpture. Sculpture of gena: only with weakly rugulose sculpture.

*Mesosoma*. Dorsal mesosoma in lateral view: convex. Sculpture of pronotal cervical area: with small (at most as large as crenulae on anterior edge of mesoscutum), weakly defined foveae. Sculpture of pronotal lateral area: transversely costate. Netrion: absent, obscured by longitudinal sculpture of lateral pronotum. Notaulus: present, simple. Length of notaulus: approximately 0.5× length of mesoscutum. Width of notaulus: narrow (notaulus width less than or equal to half the width of tegula). Sculpture of mesoscutum: granulose, posteriorly with conspicuous smooth areas. Sculpture of mesoscutellum: smooth or nearly so. Mesoscutellar profile: mainly flat, anterior and posterior edge at same height or nearly so. Mesoscutellar shape: flat, not depressed. Lateral propodeal area: sparsely transversely carinate. Shape of propodeal anterior spine: short, broad, apex rounded. Sculpture of propodeum between anterior spines: longitudinally costate. Sculpture of ventral half of mesepisternum: smooth or nearly so. Sculpture of upper 1/4 of mesopleuron: densely longitudinally costate across entire width. Metapleural sculpture: midtransverse area smooth, otherwise with cristate, longitudinal carinae.

*Wings*. Stigmal vein: present, short, broad. Campaniform sensilla at distal area of stigmal vein: present.

*Metasoma*. Shape of T1 horn: narrow, elongate. Sculpture of upper portion of T1 horn: smooth. Sculpture of posterior portion of T1 horn: largely smooth, with sparse longitudinal carinae. Lateral carinae on T2: absent. Sculpture of T2: meson and posterior margin smooth, otherwise longitudinally costate. Sculpture of T3: coriaceous. Sculpture of S3–S6: finely, weakly coriaceous. S2 anterior carina: present, rounded, uninterrupted.

*Male*. Unknown.

##### Diagnosis.

This species can be separated from all other species with an obscured netrion by the subtriangular area below the eyes (seen in anterior view) that narrows towards the mandibles, unsculptured notauli, and non-ornamented occipital carina.

##### Etymology.

This species is named after our colleague and arachnologist Dr Mark Harvey from the Western Australian Museum, who also manages to collect numerous parasitic Hymenoptera. The epithet is a noun in the genitive case.

##### Link to distribution map.

60

##### Material examined.

Holotype female: **AUSTRALIA**: WA, airport site, PA 5, Perth, 31°58'03"S, 115°58'11"E, 11.XI–6.I.1994, pitfall trap, J. M. Waldock, K. Goodsell & J. Webb, OSUC 237921 (deposited in WAMP). *Paratypes*: **AUSTRALIA**: 12 females, OSUC 237917 (OSUC); OSUC 237918–237919, 237922–237929 (WAMP); OSUC 237930 (WINC).

##### Comments.

The holotype is in good condition except for the right wings which are missing. The paratypes are in good condition except OSUC 237919 which has the metasoma detached and glued to the wings.

In some female specimens the color of the mesoscutum varies from completely yellow to almost completely brown. Additionally, the color of the lateral portions of the T1 horn may vary from completely yellow to almost completely light brown.

#### Odontacolus heratyi

Valerio & Austin sp. n.

urn:lsid:zoobank.org:act:892F4167-310A-425E-B414-C0FE8360761D

urn:lsid:biosci.ohio-state.edu:osuc_concepts:254268

http://species-id.net/wiki/Odontacolus_heratyi

Figures 11 , 167–172 ; Morphbank ^61^

##### Description.

*Female*. Body length: 1.40 – 1.82 mm (n=3). Antenna color: completely yellow. Body color: head and mesosoma dark brown, T1 almost completely honey yellow, metasoma otherwise brown, lighter in color than head or mesosoma. Coxae color: yellow. Leg color (excluding coxae): yellow. Fore wing color: slightly infuscate throughout.

*Head*. Size of compound eye: approximately 1/2× height of head. Head shape in lateral view: lower head elongate and broad at mouth, head appearing elongate and somewhat thin. Sculpture of antennal scrobe: weakly rugulose throughout. Surface of torular triangle: flat. Development of central keel on frons: present, elongate (equal to or greater than 1/3× height of frons), but not reaching anterior ocellus. Sculpture on upper frons below anterior ocellus: with weak rugulose-aciculate sculpture. Sculpture of malar space: with weak rugulose sculpture mixed with granulate sculpture. Furrow at lateral portion of antennal scrobe: absent. Mesal surface of vertex: flat to weakly convex. Size of lateral ocelli: large. Distance between lateral ocellus and occipital carina: less than 0.5× maximum ocellar diameter. Lagrimal: absent or minute. Length of OOL: less than or equal to 1/3× width of ocellus. Sculpture of vertex: with abundant areolate sculpture mixed with dense, fine granulae. Sculpture of occipital carina: weakly crenulate throughout. Distance from occipital carina to orbital carina: slightly greater than width of occipital carina. Shape of occipital carina: weakly sinuate medially. Sculpture of occiput: with weakly rugulo aciculate sculpture. Sculpture of gena: with weak rugulose sculpture and granulate background sculpture.

*Mesosoma*. Dorsal mesosoma in lateral view: convex. Sculpture of pronotal cervical area: with small (at most as large as crenulae on anterior edge of mesoscutum), well-defined foveae. Sculpture of pronotal lateral area: largely smooth, dorsal margin with dense, weak punctulae. Netrion: present, smooth, linear. Notaulus: absent. Length of notaulus: not applicable, notauli absent. Width of notaulus: not applicable, notauli absent. Sculpture of mesoscutum: weakly rugulose mixed with weak granulae. Sculpture of mesoscutellum: weakly rugulose mixed with granulate sculpture. Mesoscutellar profile: mainly flat, anterior and posterior edge at same height or nearly so. Mesoscutellar shape: flat, not depressed. Lateral propodeal area: densely, finely rugulose. Shape of propodeal anterior spine: elongate, narrow, apex rounded. Sculpture of propodeum between anterior spines: longitudinally costate. Sculpture of ventral half of mesepisternum: weakly coriaceous. Sculpture of upper 1/4 of mesopleuron: densely longitudinally costate across entire width. Metapleural sculpture: smooth.

*Wings*. Stigmal vein: present, elongate, narrow. Campaniform sensilla at distal area of stigmal vein: present.

*Metasoma*. Shape of T1 horn: narrow, elongate. Sculpture of upper portion of T1 horn: longitudinally carinate. Sculpture of posterior portion of T1 horn: largely smooth, with sparse longitudinal carinae. Lateral carinae on T2: present, well-defined. Sculpture of T2: longitudinally costate on coriaceous background. Sculpture of T3: anterior two-thirds longitudinally costate, otherwise coriaceous. Sculpture of S3–S6: finely, weakly coriaceous. S2 anterior carina: absent.

*Male*. Unknown.

##### Diagnosis.

This species can be distinguished from the other species from Fiji, all of which lack notauli, by the convex mesosoma, and the short distance between the posterior ocellus and occipital carina (approximately 0.5× the ocellar diameter).

##### Etymology.

This species is named after our friend and colleague Dr John Heraty, from the University of California, Riverside. The epithet is a noun in the genitive case.

##### Link to distribution map.

62

##### Material examined.

Holotype female: **FIJI**: Western Div., Ba Prov., Viti Levu Isl., Eteni, FJ_11a, Navai, 17°37'S, 177°59'E, 700m, 15.V–2.VII.2003, malaise trap, M. Irwin, E. Schlinger & M. Tokota’a, FBA029316 (deposited in BPBM). *Paratypes*: **FIJI**: 3 females, FBA014379, FBA041608 (BPBM); OSUC 237934 (CNCI).

##### Comments.

The holotype and paratypes are in perfect condition.

#### Odontacolus heydoni

Valerio & Austin sp. n.

urn:lsid:zoobank.org:act:8D42F907-EA56-4875-91C8-96330AFB3F4B

urn:lsid:biosci.ohio-state.edu:osuc_concepts:254271

http://species-id.net/wiki/Odontacolus_heydoni

Figures 41–42 , 173–178 ; Morphbank ^63^

##### Description.

*Female*. Body length: 1.69 – 1.98 mm (n=3). Antenna color: completely yellow except distal half of clava slightly darker than remainder of antenna. Body color: mostly yellow, distal half of T1, margins and mesal areas of T2–T3, T4–T6 honey yellow. Coxae color: yellow. Leg color (excluding coxae): yellow. Fore wing color: completely hyaline.

*Head*. Size of compound eye: approximately 1/2× height of head. Head shape in lateral view: lower head elongate and broad at mouth, head appearing elongate and somewhat thin. Sculpture of antennal scrobe: coriaceous throughout. Surface of torular triangle: flat. Development of central keel on frons: present, elongate (equal to or greater than 1/3× height of frons), but not reaching anterior ocellus. Sculpture on upper frons below anterior ocellus: with sparse, transverse costae mixed with weak, dense granulae. Sculpture of malar space: granulose throughout, without fan-like striae. Furrow at lateral portion of antennal scrobe: absent. Mesal surface of vertex: flat to weakly convex. Size of lateral ocelli: large. Distance between lateral ocellus and occipital carina: 0.5–1.2× maximum ocellar diameter. Lagrimal: absent or minute. Length of OOL: less than or equal to 1/3× width of ocellus. Sculpture of vertex: granulate. Sculpture of occipital carina: largely simple, at most with sparse weak crenulae medially. Distance from occipital carina to orbital carina: at least 2× width of occipital carina. Shape of occipital carina: simply arcuate medially. Sculpture of occiput: with weak, small granulae. Sculpture of gena: granulose.

*Mesosoma*. Dorsal mesosoma in lateral view: convex. Sculpture of pronotal cervical area: with small (at most as large as crenulae on anterior edge of mesoscutum), well-defined foveae. Sculpture of pronotal lateral area: coriaceous. Netrion: present, smooth, well developed, sub-obovate. Notaulus: present, simple. Length of notaulus: approximately less than or equal to 1/3 of length of mesoscutum. Width of notaulus: narrow (notaulus width less than or equal to half the width of tegula). Sculpture of mesoscutum: finely granulose. Sculpture of mesoscutellum: with weak, fine, granulate sculpture. Mesoscutellar profile: mainly flat, anterior and posterior edge at same height or nearly so. Mesoscutellar shape: flat, not depressed. Lateral propodeal area: sparsely transversely carinate. Shape of propodeal anterior spine: elongate, narrow, apex rounded. Sculpture of propodeum between anterior spines: smooth or largely smooth. Sculpture of ventral half of mesepisternum: weakly coriaceous. Sculpture of upper 1/4 of mesopleuron: densely longitudinally costate across half width. Metapleural sculpture: largely smooth except lower half with longitudinal carinae.

*Wings*. Stigmal vein: present, elongate, narrow. Campaniform sensilla at distal area of stigmal vein: present.

*Metasoma*. Shape of T1 horn: narrow, elongate. Sculpture of upper portion of T1 horn: longitudinally carinate. Sculpture of posterior portion of T1 horn: largely smooth, with sparse longitudinal carinae. Lateral carinae on T2: present, well-defined. Sculpture of T2: longitudinally costate on coriaceous background. Sculpture of T3: weakly coriaceous. Sculpture of S3–S6: finely, weakly coriaceous. S2 anterior carina: present, cristate, uninterrupted.

Male. Unknown.

##### Diagnosis.

*Odontacolus heydoni* belongs to a group of species that has the occipital carina separated from the orbital carina, a well-defined netrion, a central keel on the frons, and well-defined notauli. Along with *Odontacolus cardaleae* it is the only species in the group that has a few, broad crenulae across the notauli and the antennal scrobes with transverse sinuate ridges. *Odontacolus heydoni* can be separated from *Odontacolus cardaleae* by its large ocelli (Fig. 40) and the short distance between the lateral ocellus and occipital carina (approximately 0.5× ocellar diameter); *Odontacolus cardaleae* has small ocelli and the distance between lateral ocellus and occipital carina is approximately 1.5× the ocellar diameter.

##### Etymology.

This species is named after Dr Steve Heydon at the Bohart Museum, who collected this magnificent species. The epithet is a noun in the genitive case.

##### Link to distribution map.

64

##### Material examined.

Holotype female: **MALAYSIA**: Sarawak St., Borneo Isl., SW of Mount Buda, 04°13'N, 114°56'E, 22.XI–28.XI.1996, Malaise trap, S. Heydon, OSUC 239203 (deposited in CNCI). *Paratypes*: **THAILAND**: 2 females, OSUC 238768 (CNCI); OSUC 268731 (OSUC).

##### Comments.

The holotype is in perfect condition as are the paratypes except for OSUC 238768 which has the right antenna missing. Some specimens have the body color completely yellow and without darker areas on the metasomal terga.

#### Odontacolus irwini

Valerio & Austin sp. n.

urn:lsid:zoobank.org:act:FFD246F0-54BE-4DCD-92BD-1C7671D55D35

urn:lsid:biosci.ohio-state.edu:osuc_concepts:254275

http://species-id.net/wiki/Odontacolus_irwini

Figures 16 , 179–184 ; Morphbank ^65^

##### Description.

*Female*. Body length: 1.04 mm (n=1). Antenna color: completely yellow. Body color: head and mesosoma dark brown, metasoma honey yellow. Coxae color: yellow. Leg color (excluding coxae): yellow. Fore wing color: slightly infuscate throughout.

*Head*. Size of compound eye: approximately 1/2× height of head. Head shape in lateral view: lower head elongate and broad at mouth, head appearing elongate and somewhat thin. Sculpture of antennal scrobe: weakly granulose throughout. Surface of torular triangle: slightly bulging. Development of central keel on frons: present, short (less than 1/3 of frons height). Sculpture on upper frons below anterior ocellus: granulose throughout. Sculpture of malar space: with weak rugulose sculpture mixed with granulate sculpture. Furrow at lateral portion of antennal scrobe: absent. Mesal surface of vertex: flat to weakly convex. Size of lateral ocelli: normal. Distance between lateral ocellus and occipital carina: 0.5–1.2× maximum ocellar diameter. Lagrimal: absent or minute. Length of OOL: less than or equal to 1/3× width of ocellus. Sculpture of vertex: granulate. Sculpture of occipital carina: weakly crenulate throughout. Distance from occipital carina to orbital carina: at least 2× width of occipital carina. Shape of occipital carina: weakly sinuate medially. Sculpture of occiput: with weak, small granulae. Sculpture of gena: with weak rugulose sculpture and granulate background sculpture.

*Mesosoma*. Dorsal mesosoma in lateral view: convex. Sculpture of pronotal cervical area: with small (at most as large as crenulae on anterior edge of mesoscutum), weakly defined foveae. Sculpture of pronotal lateral area: largely smooth, dorsal margin with dense, weak punctulae. Netrion: present, smooth, well-developed, not looking linear. Notaulus: absent. Length of notaulus: not applicable, notauli absent. Width of notaulus: not applicable, notauli absent. Sculpture of mesoscutum: finely granulose. Sculpture of mesoscutellum: with weak, fine, granulate sculpture. Mesoscutellar profile: mainly flat, anterior and posterior edge at same height or nearly so. Mesoscutellar shape: flat, not depressed. Lateral propodeal area: longitudinally costate. Shape of propodeal anterior spine: short, broad, apex subtriangular. Sculpture of propodeum between anterior spines: smooth or largely smooth. Sculpture of ventral half of mesepisternum: smooth or nearly so. Sculpture of upper 1/4 of mesopleuron: smooth. Metapleural sculpture: smooth.

*Wings*. Stigmal vein: present, elongate, narrow. Campaniform sensilla at distal area of stigmal vein: present.

*Metasoma*. Shape of T1 horn: broad, short. Sculpture of upper portion of T1 horn: longitudinally carinate. Sculpture of posterior portion of T1 horn: largely smooth, with sparse longitudinal carinae. Lateral carinae on T2: absent. Sculpture of T2: meson and posterior margin smooth, otherwise longitudinally costate. Sculpture of T3: weakly coriaceous. Sculpture of S3–S6: mainly smooth, with sparse, setigerous punctulae. S2 anterior carina: absent.

*Male*. Unknown.

##### Diagnosis.

This species can be distinguished from *Odontacolus veroae* by the cicatrose sculpture on the posterior sublateral areas of the mesoscutum, the remainder of the sclerite having dense, weak, granulate sculpture; and the large body size (1.47–1.71 mm). Additionally, within the Fijian group of species, *Odontacolus irwini* can be separated from *Odontacolus heratyi* by the wider space between the occipital carina and the ocular carina, and from *Odontacolus schlingeri* by the convex mesosoma.

##### Etymology.

This species is named after the dipterist Dr Mike Irwin, who collected the type series. The epithet is a noun in the genitive case.

##### Link to distribution map.

66

##### Material examined.

Holotype female: **FIJI**: Western Div., Ba Prov., Viti Levu Isl., Eteni, FJ_11D, Navai, 17°37'S, 177°59'E, 700m, 24.X–8.XI.2003, Malaise trap, M. Irwin, E. Schlinger & M. Tokota’a, FBA021030 (deposited in BPBM). *Paratypes*: **FIJI**: 3 females, FBA014433 (BPBM), FBA059072 (FNIC); FBA074688 (OSUC).

##### Comments.

The holotype is in perfect condition as are the paratypes.

#### Odontacolus jacksonae

Valerio & Austin sp. n.

urn:lsid:zoobank.org:act:F80D0041-7F6D-47ED-9D3F-80007373DFB8

urn:lsid:biosci.ohio-state.edu:osuc_concepts:254256

http://species-id.net/wiki/Odontacolus_jacksonae

Figures 1 , 35 , 65 , 185–190 ; Morphbank ^67^

##### Description.

*Female*. Body length: 1.75 – 1.78 mm (n=2). Antenna color: A1 yellow, otherwise dark brown. Body color: completely dark brown. Coxae color: honey yellow. Leg color (excluding coxae): legs yellow with hind femora honey yellow. Fore wing color: slightly infuscate throughout.

*Head*. Size of compound eye: approximately 1/2× height of head. Head shape in lateral view: lower head elongate and broad at mouth, head appearing elongate and somewhat thin. Sculpture of antennal scrobe: largely smooth ventrally, sparsely granulate ventrally. Surface of torular triangle: slightly bulging. Development of central keel on frons: present, elongate (equal to or greater than 1/3× height of frons), but not reaching anterior ocellus. Sculpture on upper frons below anterior ocellus: with weak rugulose-aciculate sculpture. Sculpture of malar space: with sparse, short fan-like striae, striae not extending into scrobal area, mixed with weak coriaceous sculpture. Furrow at lateral portion of antennal scrobe: absent. Mesal surface of vertex: flat to weakly convex. Size of lateral ocelli: normal. Distance between lateral ocellus and occipital carina: 0.5–1.2× maximum ocellar diameter. Lagrimal: absent or minute. Length of OOL: less than or equal to 1/3× width of ocellus. Sculpture of vertex: granulate. Sculpture of occipital carina: weakly crenulate throughout. Distance from occipital carina to orbital carina: contiguous or nearly so, subequal to width of occipital carina. Shape of occipital carina: simply arcuate medially. Sculpture of occiput: with weakly rugulo aciculate sculpture. Sculpture of gena: granulose.

*Mesosoma*. Dorsal mesosoma in lateral view: convex. Sculpture of pronotal cervical area: with small (at most as large as crenulae on anterior edge of mesoscutum), well-defined foveae. Sculpture of pronotal lateral area: dorsally punctate, otherwise smooth. Netrion: present, smooth, linear. Notaulus: present, simple. Length of notaulus: approximately less than or equal to 1/3 of length of mesoscutum. Width of notaulus: narrow (notaulus width less than or equal to half the width of tegula). Sculpture of mesoscutum: finely granulose. Sculpture of mesoscutellum: granulose. Mesoscutellar profile: mainly flat, anterior and posterior edge at same height or nearly so. Mesoscutellar shape: flat, not depressed. Lateral propodeal area: coarsely rugulose. Shape of propodeal anterior spine: elongate, narrow, apex sharply acute. Sculpture of propodeum between anterior spines: smooth or largely smooth. Sculpture of ventral half of mesepisternum: weakly, finely coriaceous. Sculpture of upper 1/4 of mesopleuron: densely longitudinally costate across entire width. Metapleural sculpture: largely smooth except lower half with longitudinal carinae.

*Wings*. Stigmal vein: present, elongate, narrow. Campaniform sensilla at distal area of stigmal vein: present.

*Metasoma*. Shape of T1 horn: narrow, elongate. Sculpture of upper portion of T1 horn: longitudinally carinate. Sculpture of posterior portion of T1 horn: longitudinally carinate. Lateral carinae on T2: present, poorly defined. Sculpture of T2: finely longitudinally costate, granulate laterally. Sculpture of T3: smooth mesally, area flanking mesal smooth area weakly costate in anterior third, otherwise weakly coriaceous. Sculpture of S3–S6: S3 weakly granulose, S4–S6 weakly, finely coriaceous. S2 anterior carina: present, cristate, interrupted medially.

*Male*. Unknown.

##### Diagnosis.

Within the group of species with a netrion present, the occipital carina almost touching the orbital carina, and having a sculptured gena, *Odontacolus jacksonae* can be separated from *Odontacolus gentingensis* by having a reduced lagrimal; in *Odontacolus gentingensis* the lagrimal is larger and very conspicuous.

##### Etymology.

This species is named after Ms Dorothy Jackson, who collected Hymenoptera for the BMNH in Cameroon, including this species. The epithet is a noun in the genitive case.

##### Link to distribution map.

68

##### Material examined.

Holotype female: **CAMEROON**: Nkoemvom, IV-1980–V-1980, D. Jackson, OSUC 238415 (deposited in BMNH). *Paratypes*: (17 females) **CAMEROON**: 13 females, OSUC 238414, 238416, 238432–238433, 238440–238441, 238443, 321892–321893, 321896(BMNH); OSUC 321898(WINC); OSUC 238420, 238434 (CNCI). **GUINEA**: 2 females, OSUC 238417 (BMNH); OSUC 238431 (CNCI). **MADAGASCAR**: 2 females, CASENT 2079143 (CASC); OSUC 229796 (OSUC).

##### Comments.

The holotype is in perfect condition as are the paratypes.

#### Odontacolus kiau

Valerio & Austin sp. n.

urn:lsid:zoobank.org:act:EEC2DDAA-3DB7-4427-AF41-30B897C3BA96

urn:lsid:biosci.ohio-state.edu:osuc_concepts:280211

http://species-id.net/wiki/Odontacolus–kiau

Figures 59 , 191–196 ; Morphbank ^69^

##### Description.

*Female*. Body length: 1.68 mm (n=1). Antenna color: completely yellow. Body color: completely dark brown. Coxae color: dark brown. Leg color (excluding coxae): honey yellow. Fore wing color: slightly infuscate throughout.

*Head*. Size of compound eye: approximately 1/2× height of head. Head shape in lateral view: lower head elongate and broad at mouth, head appearing elongate and somewhat thin. Sculpture of antennal scrobe: largely smooth ventrally, dorsally with sinuate, transverse ridges.Surface of torular triangle: flat. Development of central keel on frons: present, short (less than 1/3 of frons height). Sculpture on upper frons below anterior ocellus: with sparse, transverse costae mixed with weak, dense granulae. Sculpture of malar space: weakly rugulose throughout, without fan-like striae. Furrow at lateral portion of antennal scrobe: absent. Mesal surface of vertex: flat to weakly convex. Size of lateral ocelli: large. Distance between lateral ocellus and occipital carina: 0.5–1.2× maximum ocellar diameter. Lagrimal: absent or minute. Length of OOL: less than or equal to 1/3× width of ocellus. Sculpture of vertex: granulate. Sculpture of occipital carina: weakly crenulate throughout. Distance from occipital carina to orbital carina: at least 2× width of occipital carina. Shape of occipital carina: simply arcuate medially. Sculpture of occiput: with weakly rugulo aciculate sculpture. Sculpture of gena: granulose.

*Mesosoma*. Dorsal mesosoma in lateral view: convex. Sculpture of pronotal cervical area: with small (at most as large as crenulae on anterior edge of mesoscutum), well-defined foveae. Sculpture of pronotal lateral area: coriaceous. Netrion: present, smooth, well developed, sub-obovate. Notaulus: present, simple. Length of notaulus: approximately less than or equal to 1/3 of length of mesoscutum. Width of notaulus: narrow (notaulus width less than or equal to half the width of tegula). Sculpture of mesoscutum: weakly rugulose mixed with weak granulae. Sculpture of mesoscutellum: granulose. Mesoscutellar profile: elevated, anterior margin higher than posterior. Mesoscutellar shape: flat, not depressed. Lateral propodeal area: densely, finely rugulose. Shape of propodeal anterior spine: short, broad, apex rounded. Sculpture of propodeum between anterior spines: smooth or largely smooth. Sculpture of ventral half of mesepisternum: smooth or nearly so. Sculpture of upper 1/4 of mesopleuron: densely longitudinally costate across entire width. Metapleural sculpture: mainly with weak coriaceous sculpture, lower 1/3 without longitudinal costae.

*Wings*. Stigmal vein: present, elongate, narrow. Campaniform sensilla at distal area of stigmal vein: present.

*Metasoma*. Shape of T1 horn: narrow, elongate. Sculpture of upper portion of T1 horn: longitudinally carinate. Sculpture of posterior portion of T1 horn: largely smooth, with sparse longitudinal carinae. Lateral carinae on T2: present, well-defined. Sculpture of T2: longitudinally costate on weak coriaceous background. Sculpture of T3: weakly coriaceous. Sculpture of S3–S6: S3 weakly granulose, S4–S6 weakly, finely coriaceous. S2 anterior carina: absent.

*Male*. Unknown.

##### Diagnosis.

*Odontacolus kiau* is very similar to *Odontacolus whitfieldi*, but the former lacks an anterior transverse carina on S2; *Odontacolus whitfieldi* has this carina complete and cristate. Thesespecies belong to a group that have short, smooth notauli, a well-defined netrion, a central keel on the frons, and the sculpture of the frons always has transverse costae. Additionally, *Odontacolus kiau* can be separated from *Odontacolus mayri* by the dark brown coxae and the slightly bulging torular triangle observed on the former; in *Odontacolus mayri* the coxae are yellow and the torular triangle is flat.

##### Etymology.

This species is named after the East New Britain word ‘kiau’ (in Kuanua language) which means ‘egg’, and refers to the stage of the host parasitized by *Odontacolus*. The name is used as a noun in apposition.

##### Link to distribution map.

70

##### Material examined.

Holotype female: **PAPUA NEW GUINEA**: East New Britain Prov., DPI base camp, Baining Mountains, 04°26.36'S, 151°49.02'E, 28.X–11.XI.1999, flight intercept trap, A. Mararuai & M. Kalamen, OSUC 239159 (deposited in CNCI).

##### Comments.

The holotype is in perfect condition.

#### Odontacolus lamarcki

Valerio & Austin sp. n.

urn:lsid:zoobank.org:act:A7EDDF35-0A42-47C0-AB41-14FCA5812BBD

urn:lsid:biosci.ohio-state.edu:osuc_concepts:280244

http://species-id.net/wiki/Odontacolus_lamarcki

Figures 45 , 66 , 197–202 ; Morphbank ^71^

##### Description.

*Female*. Body length: 1.19 mm (n=1). Antenna color: completely yellow. Body color: head and metasoma honey yellow except for T1 horn which is darker than remainder of metasoma color, mesosoma yellow. Coxae color: whitish yellow. Leg color (excluding coxae): yellow. Fore wing color: slightly infuscate throughout.

*Head*. Size of compound eye: approximately 1/2× height of head. Head shape in lateral view: lower head elongate and broad at mouth, head appearing elongate and somewhat thin. Sculpture of antennal scrobe: largely smooth ventrally, dorsally with sinuate, transverse ridges. Surface of torular triangle: slightly bulging. Development of central keel on frons: completely absent. Sculpture on upper frons below anterior ocellus: granulose throughout. Sculpture of malar space: granulose throughout. Furrow at lateral portion of antennal scrobe: absent. Mesal surface of vertex: flat to weakly convex. Size of lateral ocelli: normal. Distance between lateral ocellus and occipital carina: 0.5–1.2× maximum ocellar diameter. Lagrimal: absent or minute. Length of OOL: less than or equal to 1/3× width of ocellus. Sculpture of vertex: granulate. Sculpture of occipital carina: weakly crenulate throughout. Distance from occipital carina to orbital carina: at least 2× width of occipital carina. Shape of occipital carina: simply arcuate medially. Sculpture of occiput: with weak, small granulae. Sculpture of gena: granulose.

*Mesosoma*. Dorsal mesosoma in lateral view: convex. Sculpture of pronotal cervical area: with small (at most as large as crenulae on anterior edge of mesoscutum), weakly defined foveae. Sculpture of pronotal lateral area: upper 1/3 granulose, lower 1/3 with transverse foveae, otherwise smooth. Netrion: present, smooth, well developed, sub-obovate. Notaulus: present, simple. Length of notaulus: approximately less than or equal to 1/3 of length of mesoscutum. Width of notaulus: narrow (notaulus width less than or equal to half the width of tegula). Sculpture of mesoscutum: weakly rugulose mixed with weak granulae. Sculpture of mesoscutellum: granulose. Mesoscutellar profile: mainly flat, anterior and posterior edge at same height or nearly so. Mesoscutellar shape: flat, not depressed. Lateral propodeal area: longitudinally costate. Shape of propodeal anterior spine: elongate, narrow, apex rounded. Sculpture of propodeum between anterior spines: smooth or largely smooth. Sculpture of ventral half of mesepisternum: smooth or nearly so. Sculpture of upper 1/4 of mesopleuron: densely longitudinally costate across half width. Metapleural sculpture: largely smooth except lower half with longitudinal carinae.

*Wings*. Stigmal vein: present, elongate, narrow. Campaniform sensilla at distal area of stigmal vein: present.

*Metasoma*. Shape of T1 horn: broad, short. Sculpture of upper portion of T1 horn: longitudinally carinate. Sculpture of posterior portion of T1 horn: largely smooth, with sparse longitudinal carinae. Lateral carinae on T2: present, well-defined. Sculpture of T2: largely weakly coriaceous mixed with longitudinal costae, meson coriaceous. Sculpture of T3: smooth mesally, otherwise weakly coriaceous. Sculpture of S3–S6: finely, weakly coriaceous. S2 anterior carina: present, cristate, uninterrupted.

*Male*. Unknown.

##### Diagnosis.

Along with *Odontacolus wallacei* this is the only other species without a medial facial keel in combination with a well-defined netrion, and the occipital carina being separated from the orbital carina. *Odontacolus lamarcki* can be separated from *Odontacolus wallacei* by the short distance between the occipital carina and the lateral ocellus (approximately less than or equal to 0.6× ocellus diameter); in *Odontacolus wallacei* the distance between the occipital carina and the lateral ocellus is approximately equal to or greater than 1.2×.

##### Etymology.

This species is named after the great French zoologist Jean-Baptiste Lamarck. The epithet is a noun in the genitive case.

##### Link to distribution map.

72

##### Material examined.

Holotype female: **THAILAND**: Phetchabun Prov., mixed deciduous forest, T1398, Khao Kho National Park, 16°39.589'N, 101°08.185'E, 168m, 19.I–26.I.2007, Malaise trap, S. Chachumnan & S. Singtong, OSUC 339597 (deposited in QSBG).

##### Comments.

The holotype specimen is in good condition.

#### Odontacolus longiceps

Kieffer

urn:lsid:zoobank.org:act:E67B7102-8BE1-4519-8509-63FAEF48E40C

urn:lsid:biosci.ohio-state.edu:osuc_concepts:4948

http://species-id.net/wiki/Odontacolus_longiceps

Figures 44 , 203–208 ; Morphbank ^73^

Odontacolus longiceps Kieffer 1910a: 294 (original description); Kieffer 1912a: 54 (redescribed as new); Kieffer 1926: 145 (description, keyed); Masner 1965: 85 (type information).

##### Description.

*Female*. Body length: 1.29 mm (n=1). Antenna color: A1 yellow, otherwise dark brown. Body color: mostly dark brown, head and dorsal mesosoma darker than remainder of body, T1 and anterior margin of T2 yellow. Coxae color: yellow. Leg color (excluding coxae): yellow. Fore wing color: slightly infuscate throughout.

*Head*. Size of compound eye: approximately 1/2× height of head. Head shape in lateral view: lower head elongate and broad at mouth, head appearing elongate and somewhat thin. Sculpture of antennal scrobe: smooth throughout. Surface of torular triangle: flat. Development of central keel on frons: completely absent. Sculpture on upper frons below anterior ocellus: granulose throughout. Sculpture of malar space: granulose throughout, without fan-like striae. Furrow at lateral portion of antennal scrobe: absent. Mesal surface of vertex: flat to weakly convex. Size of lateral ocelli: normal. Distance between lateral ocellus and occipital carina: 0.5–1.2× maximum ocellar diameter. Lagrimal: absent or minute. Length of OOL: less than or equal to 1/3× width of ocellus. Sculpture of vertex: granulate. Sculpture of occipital carina: largely simple, at most with sparse weak crenulae medially. Distance from occipital carina to orbital carina: at least 2× width of occipital carina. Shape of occipital carina: simply arcuate medially. Sculpture of occiput: with weak, small granulae. Sculpture of gena: granulose.

*Mesosoma*. Dorsal mesosoma in lateral view: convex. Sculpture of pronotal lateral area: largely smooth, dorsal margin with dense, weak punctulae. Netrion: present, smooth, linear. Notaulus: present, simple. Length of notaulus: approximately less than or equal to 1/3 of length of mesoscutum. Width of notaulus: narrow (notaulus width less than or equal to half the width of tegula). Sculpture of mesoscutum: coriaceous. Sculpture of mesoscutellum: weakly coriaceous. Mesoscutellar profile: mainly flat, anterior and posterior edge at same height or nearly so. Mesoscutellar shape: flat, not depressed. Lateral propodeal area: longitudinally costate. Shape of propodeal anterior spine: short, broad, apex subtriangular. Sculpture of propodeum between anterior spines: smooth or largely smooth; with strong, oblique ridges laterally. Sculpture of ventral half of mesepisternum: smooth or nearly so. Sculpture of upper 1/4 of mesopleuron: densely longitudinally costate across half width. Metapleural sculpture: largely smooth except lower half with longitudinal carinae.

*Wings*. Stigmal vein: present, elongate, narrow. Campaniform sensilla at distal area of stigmal vein: present.

*Metasoma*. Shape of T1 horn: broad, short. Sculpture of upper portion of T1 horn: longitudinally carinate. Sculpture of posterior portion of T1 horn: smooth. Lateral carinae on T2: present, poorly defined. Sculpture of T2: longitudinally costate on coriaceous background. Sculpture of T3: coriaceous. Sculpture of S3–S6: mainly smooth, with sparse, setigerous punctulae. S2 anterior carina: present, cristate, uninterrupted.

*Male*. Unknown.

##### Diagnosis.

This species can be easily separated from all other *Odontacolus* with smooth, short notauli and a well-developed netrion by the combination of the completely dark brown body, the nearly smooth posterior portion of the mesoscutum (between the notaulus and tegula), and the nearly smooth posterior 1/3 and mesal region of the mesoscutellum.

##### Link to distribution map.

74

##### Material examined.

Holotype female: **SEYCHELLES**: Mahé Island, 1908–1909, B.M. TYPE HYM. 9.400 (deposited in BMNH).

##### Comments.

The holotype is in good condition except that the right hind wing and right antenna are detached from body and glued to the point.

#### Odontacolus madagascarensis

Valerio & Austin sp. n.

urn:lsid:zoobank.org:act:8C05170C-EC87-47C4-8339-FB13F6CAD954

urn:lsid:biosci.ohio-state.edu:osuc_concepts:254269

http://species-id.net/wiki/Odontacolus_madagascarensis

Figures 26 , 30 , 209–214 ; Morphbank ^75^

##### Description.

*Female*. Body length: 1.25 mm (n=2). Antenna color: completely yellow. Body color: completely dark brown. Coxae color: yellow. Leg color (excluding coxae): yellow. Fore wing color: completely hyaline.

*Head*. Size of compound eye: approximately 1/2× height of head. Head shape in lateral view: lower head elongate and broad at mouth, head appearing elongate and somewhat thin. Sculpture of antennal scrobe: smooth throughout. Surface of torular triangle: flat. Development of central keel on frons: present, elongate (equal to or greater than 1/3× height of frons), but not reaching anterior ocellus. Sculpture on upper frons below anterior ocellus: largely smooth, with sparse coriaceous sculpture close to compound eyes. Sculpture of malar space: largely smooth, coriaceous sculpture present near compound eyes (somewhat weak). Furrow at lateral portion of antennal scrobe: absent. Mesal surface of vertex: flat to weakly convex. Size of lateral ocelli: small. Distance between lateral ocellus and occipital carina: 0.5–1.2× maximum ocellar diameter. Lagrimal: absent or minute. Length of OOL: less than or equal to 1/3× width of ocellus. Sculpture of vertex: coriaceous. Sculpture of occipital carina: largely simple, at most with sparse weak crenulae medially. Distance from occipital carina to orbital carina: slightly greater than width of occipital carina. Shape of occipital carina: simply arcuate medially. Sculpture of occiput: with weakly rugulo aciculate sculpture. Sculpture of gena: coriaceous dorsally, otherwise smooth.

*Mesosoma*. Dorsal mesosoma in lateral view: convex. Sculpture of pronotal cervical area: with small (at most as large as crenulae on anterior edge of mesoscutum), well-defined foveae. Sculpture of pronotal lateral area: largely smooth, dorsal margin with dense, weak punctulae. Netrion: present, smooth, linear. Notaulus: present, simple. Length of notaulus: approximately less than or equal to 1/3 of length of mesoscutum. Width of notaulus: narrow (notaulus width less than or equal to half the width of tegula). Sculpture of mesoscutum: coriaceous. Sculpture of mesoscutellum: weakly coriaceous. Mesoscutellar profile: mainly flat, anterior and posterior edge at same height or nearly so. Mesoscutellar shape: flat, not depressed. Lateral propodeal area: densely, finely rugulose. Shape of propodeal anterior spine: short, broad, apex subtriangular. Sculpture of propodeum between anterior spines: smooth or largely smooth. Sculpture of ventral half of mesepisternum: smooth or nearly so. Sculpture of upper 1/4 of mesopleuron: densely longitudinally costate across half width. Metapleural sculpture: smooth.

*Wings*. Stigmal vein: present, elongate, narrow. Campaniform sensilla at distal area of stigmal vein: present.

*Metasoma*. Shape of T1 horn: narrow, short. Sculpture of upper portion of T1 horn: largely smooth, with sparse longitudinal carinae. Sculpture of posterior portion of T1 horn: largely smooth, with sparse longitudinal carinae. Lateral carinae on T2: present, poorly defined. Sculpture of T2: longitudinally costate, posterior margin smooth. Sculpture of T3: anterior third weakly longitudinally costate, otherwise coriaceous. Sculpture of S3–S6: mainly smooth, with sparse, setigerous punctulae. S2 anterior carina: present, cristate, uninterrupted.

*Male*. Unknown.

##### Diagnosis.

This species can be distinguished from all other *Odontacolus* species by the combination of the completely smooth gena, the well-defined netrion, the presence of notauli, and the confused, dense rugulose sculpture of the lateral propodeum.

##### Etymology.

This species is named after the island from which the species was collected, Madagascar. The epithet is used as an adjective.

##### Link to distribution map.

76

##### Material examined.

Holotype female: **MADAGASCAR**: Fianarantsoa Auto. Prov., Belle Vue trail, Ranomafana National Park, 21°15.99'S, 47°25.21'E, 1070m, 10.XII–25.XII.1999, Malaise trap, M. E. Irwin, OSUC 238728 (deposited in CASC). *Paratype*: **MADAGASCAR**: 1 female, CASENT 2134814 (CASC).

##### Comments.

The holotype and paratype are in perfect condition.

#### Odontacolus markadicus

Veenakumari

urn:lsid:zoobank.org:act:200E9F3D-87EC-49AF-AEB7-7A7981B1277A

urn:lsid:biosci.ohio-state.edu:osuc_concepts:254255

http://species-id.net/wiki/Odontacolus_markadicus

Figures 54, 56 , 215–220 , 305–306 , 312 , 316 , 331–332 ; Morphbank ^77^

##### Description.

*Female*. Body length: 1.15 – 1.72 mm (n=15). Antenna color: completely yellow. Body color: head, mesosoma, T1 (except horn), anterior portion of T2 yellow, metasoma otherwise dark brown. Coxae color: yellow. Leg color (excluding coxae): yellow. Fore wing color: completely hyaline.

*Head*. Size of compound eye: approximately 1/2× height of head. Head shape in lateral view: lower head elongate and broad at mouth, head appearing elongate and somewhat thin. Sculpture of antennal scrobe: smooth throughout; weakly coriaceous throughout. Surface of torular triangle: slightly bulging. Development of central keel on frons: present, elongate (equal to or greater than 1/3× height of frons), but not reaching anterior ocellus. Sculpture on upper frons below anterior ocellus: with sparse, transverse costae mixed with weak, dense granulae. Sculpture of malar space: coriaceous throughout, without fan-like striae. Furrow at lateral portion of antennal scrobe: absent. Mesal surface of vertex: flat to weakly convex. Size of lateral ocelli: normal. Distance between lateral ocellus and occipital carina: 0.5–1.2× maximum ocellar diameter. Lagrimal: absent or minute. Length of OOL: less than or equal to 1/3× width of ocellus. Sculpture of vertex: granulate. Sculpture of occipital carina: largely simple, at most with sparse weak crenulae medially. Distance from occipital carina to orbital carina: slightly greater than width of occipital carina. Shape of occipital carina: simply arcuate medially. Sculpture of occiput: with weak, small granulae. Sculpture of gena: granulose.

*Mesosoma*. Dorsal mesosoma in lateral view: convex. Sculpture of pronotal cervical area: with small (at most as large as crenulae on anterior edge of mesoscutum), well-defined foveae. Sculpture of pronotal lateral area: upper 1/3 granulose, lower 1/3 with transverse foveae, otherwise smooth. Netrion: present, smooth, well developed, sub-obovate. Notaulus: present, simple. Length of notaulus: approximately less than or equal to 1/3 of length of mesoscutum. Width of notaulus: distinctly widened; narrow. Sculpture of mesoscutum: weakly granulose. Sculpture of mesoscutellum: granulose. Mesoscutellar profile: elevated, anterior margin higher than posterior. Mesoscutellar shape: flat, not depressed. Lateral propodeal area: densely transversely carinate. Shape of propodeal anterior spine: elongate, narrow, apex rounded. Sculpture of propodeum between anterior spines: smooth or largely smooth. Sculpture of ventral half of mesepisternum: smooth or nearly so. Sculpture of upper 1/4 of mesopleuron: densely longitudinally costate across half width. Metapleural sculpture: mainly smooth, lower third sparsely longitudinally carinate.

*Wings*. Stigmal vein: present, elongate, narrow. Campaniform sensilla at distal area of stigmal vein: present.

*Metasoma*. Shape of T1 horn: narrow, short. Sculpture of upper portion of T1 horn: longitudinally carinate. Sculpture of posterior portion of T1 horn: largely smooth, with sparse longitudinal carinae. Lateral carinae on T2: present, well-defined. Sculpture of T2: finely longitudinally costate, granulate laterally. Sculpture of T3: anterior two-thirds weakly longitudinally costate, posterior third weakly coriaceous mesally, otherwise densely granulose. Sculpture of S3–S6: S3 weakly granulose, S4–S6 weakly, finely coriaceous. S2 anterior carina: present, cristate, uninterrupted.

*Male*. Body length: 1.45 mm (n=1). Body color: completely yellow. Sculpture of antennal scrobe: with weak coriaceous sculpture except dorsal area with sinuate, transverse carina below anterior ocellus. Shape and size of anterior ocellus: large, oblong. Vertex posterior area sculpture: densely granulate. Occipital carina dorsal area: present, cristate. Netrion: well-defined, suboval. Sculpture of mesepisternum: coriaceous. Sculpture of pronotal lateral areas: with few, straight, fine transvers carinae. Length of fore wing stigmal vein: conspicuously elongate. Angle of stigmal vein in relation to anterior margin of fore wing: at an angle of approximately 45°. Sculpture of T2: with weak longitudinal carinae mixed with weak coriaceous sculpture.

##### Diagnosis.

This species is very similar to *Odontacolus bosei* and *Odontacolus mot*. *Odontacolus markadicus* may be distinguished from *Odontacolus bosei* bythe yellow body color and the lateral occipital carina which is mainly smooth; in *Odontacolus bosei* by the body is completely dark brown and the lateral portion of the occipital carina is conspicuously crenulate. In turn, *Odontacolus markadicus* may be separated from *Odontacolus mot* by its smaller size (1.74 mm or less vs. 1.92 mm), the different sculpture on the upper frons (absent or fine and straight carinae as opposed to broad, sinuate carinae), and by the even distribution of setae on the metasoma.

##### Link to distribution map.

78

##### Type material.

*Not examined*: Holotype, female, INDIA: Karnataka: Bengaluru: Gandhi Krishi Vigyan Kendra, 29.xii.2009 at an elevation of 910m (13°2'3"N, 77°35'18"E) emerged from spider eggs (ICAR); *Paratypes*: 8 females, data same as holotype (ICAR, INCP); 1 male Karnataka: Bengaluru: Adugodi, 23.vi. 2011 (12°56'49"N, 77°36'37"E) (ICAR).

##### Material examined.

28 females, 1 male: **BRUNEI**: 1 female, OSUC 321894 (BMNH). **INDIA**: 15 females, OSUC 238769–238770, 238772, 239190–239192, 239195–239197, 239199–239201 (CNCI); OSUC 238771, 239198, 239202 (OSUC). **MALAYSIA**: 3 females, OSUC 238767, 239209, 239213 (CNCI). **THAILAND**: 7 females, 1 male, OSUC 238803–238805, 239212 (CNCI); OSUC 261831, 261852, 268732(OSUC); OSUC 321806 (WINC). **VIETNAM**: 2 females, OSUC 239211 (CNCI); OSUC 248896 (ROME).

##### Comments.

We were unable to obtain any of the type series, and so our interpretation of *Odontacolus markadicus* is based on comparision of the additional material (listed above) with the images and description in Veenakumari and Mohanraj (2011) as well as images kindly sent to us by our colleague Dr Rajmohana K.

When taking all of the available material into account some variation is evident in the females of this species: the antennal clava can be completely yellow or the distal half of the clava slightly darker, and the vertex and mesoscutum vary from completely yellow to slightly honey yellow in color. In addition, the specimens: OSUC 239209, OSUC 239211– 239213, OSUC 238767, OSUC 238803-238805, OSUC 248896, OSUC 250858, OSUC 261831, OSUC 261852, OSUC 268732, OSUC 321806 and OSUC 321894 differ from the remaining material by having the body completely yellow, but otherwise they match *Odontacolus markadicus* in every respect.

#### Odontacolus mayri

Valerio & Austin sp. n.

urn:lsid:zoobank.org:act:57EEE8E8-ED2D-4881-8D34-9983B683FD93

urn:lsid:biosci.ohio-state.edu:osuc_concepts:284013

http://species-id.net/wiki/Odontacolus_mayri

Figures 221–226 ; Morphbank ^79^

##### Description.

*Female*. Body length: 1.40 – 1.90 mm (n=14). Antenna color: completely yellow except distal half of clava slightly darker than remainder of antenna. Body color: mainly yellow, T5–T7 slightly darker. Coxae color: yellow. Leg color (excluding coxae): yellow. Fore wing color: slightly infuscate throughout.

*Head*. Size of compound eye: approximately 1/2× height of head. Head shape in lateral view: lower head elongate and broad at mouth, head appearing elongate and somewhat thin. Sculpture of antennal scrobe: largely smooth ventrally, dorsally with sinuate, transverse ridges.Surface of torular triangle: slightly bulging. Development of central keel on frons: present, elongate, reaching anterior ocellus. Sculpture on upper frons below anterior ocellus: with sparse, transverse costae mixed with weak, dense granulae. Sculpture of malar space: weakly rugulose throughout, without fan-like striae. Furrow at lateral portion of antennal scrobe: absent. Mesal surface of vertex: flat to weakly convex. Size of lateral ocelli: large. Distance between lateral ocellus and occipital carina: 0.5–1.2× maximum ocellar diameter. Lagrimal: absent or minute. Length of OOL: less than or equal to 1/3× width of ocellus. Sculpture of vertex: granulate. Sculpture of occipital carina: weakly crenulate throughout. Distance from occipital carina to orbital carina: at least 2× width of occipital carina. Shape of occipital carina: simply arcuate medially. Sculpture of occiput: with weakly rugulo aciculate sculpture. Sculpture of gena: granulose.

*Mesosoma*. Dorsal mesosoma in lateral view: convex. Sculpture of pronotal cervical area: with small (at most as large as crenulae on anterior edge of mesoscutum), well-defined foveae. Sculpture of pronotal lateral area: upper 1/3 granulose, lower 1/3 with transverse foveae, otherwise smooth. Netrion: present, smooth, well-developed, not looking linear. Notaulus: present, simple. Length of notaulus: approximately less than or equal to 1/3 of length of mesoscutum. Width of notaulus: narrow (notaulus width less than or equal to half the width of tegula). Sculpture of mesoscutum: weakly granulose. Sculpture of mesoscutellum: with weak, fine, granulate sculpture. Mesoscutellar profile: elevated, anterior margin higher than posterior. Mesoscutellar shape: flat, not depressed. Lateral propodeal area: densely, finely rugulose. Shape of propodeal anterior spine: elongate, narrow, apex rounded. Sculpture of propodeum between anterior spines: smooth or largely smooth. Sculpture of ventral half of mesepisternum: smooth or nearly so. Sculpture of upper 1/4 of mesopleuron: densely longitudinally costate across half width. Metapleural sculpture: mainly smooth, lower third sparsely longitudinally carinate.

*Wings*. Stigmal vein: present, elongate, narrow. Campaniform sensilla at distal area of stigmal vein: present.

*Metasoma*. Shape of T1 horn: broad, short. Sculpture of upper portion of T1 horn: longitudinally carinate. Sculpture of posterior portion of T1 horn: largely smooth, with sparse longitudinal carinae. Lateral carinae on T2: present, well-defined. Sculpture of T2: largely longitudinally costate, meson smooth, weakly granulate laterally. Sculpture of T3: anterior third weakly costate sublaterally, weakly coriaceous mesally, otherwise granulose. Sculpture of S3–S6: finely, weakly coriaceous. S2 anterior carina: present, cristate, uninterrupted.

*Male*. Unknown.

##### Diagnosis.

Thisspecies belongs to a group that has short, smooth notauli, a well-defined netrion, central keel present, and sculptured antennal scrobe always with transverse costae in the background. Within this group *Odontacolus mayri* can be separated from *Odontacolus kiau* and *Odontacolus whitfieldi* by its completely yellow body and the weakly granulose sculpture of the mesoscutum.

**Etymology**. This species is named after the German biologist Ernst Walter Mayr. The epithet is a noun in the genitive case.

##### Link to distribution map.

80

##### Material examined.

Holotype female: **THAILAND**: Prachuap Khiri Khan Prov., Laem Sala Beach, T3012, Khao Sam Roi Yot National Park, 12°12.234'N, 100°00.767'E, 6.VII–13.VII.2008, Malaise trap, Amnad & Yai, OSUC 321876 (deposited in QSBG). *Paratypes*: (13 females) **INDONESIA**: 1 female, OSUC 321895 (BMNH). **THAILAND**: 12 females, OSUC 339596, 339599 (CNCI); OSUC 268733, 321872, 321874, 374179 (OSUC); OSUC 250611, 250857–250858, 250860, 266235(QSBG); OSUC 339595 (WINC).

##### Comments.

The holotype is in perfect condition.

#### Odontacolus mot

Valerio & Austin sp. n.

urn:lsid:zoobank.org:act:08D833F9-97C9-4787-BCCE-D865270E9F5A

urn:lsid:biosci.ohio-state.edu:osuc_concepts:283809

http://species-id.net/wiki/Odontacolus_mot

Figures 55 , 227–232 ; Morphbank ^81^

##### Description.

*Female*. Body length: 1.92 mm (n=1). Antenna color: completely yellow. Body color: head, mesosoma, T1 (except horn), anterior portion of T2 yellow, metasoma otherwise dark brown. Coxae color: yellow. Leg color (excluding coxae): yellow. Fore wing color: completely hyaline.

*Head*. Size of compound eye: approximately 1/2× height of head. Head shape in lateral view: lower head elongate and broad at mouth, head appearing elongate and somewhat thin. Sculpture of antennal scrobe: largely smooth ventrally, dorsally with sinuate, transverse ridges. Surface of torular triangle: slightly bulging. Development of central keel on frons: present, elongate, reaching anterior ocellus. Sculpture on upper frons below anterior ocellus: covered by sinuate, transverse, fine costae. Sculpture of malar space: granulose throughout. Furrow at lateral portion of antennal scrobe: absent. Mesal surface of vertex: flat to weakly convex. Size of lateral ocelli: normal. Distance between lateral ocellus and occipital carina: 0.5–1.2× maximum ocellar diameter. Lagrimal: present, well-defined but small (length less than 0.3 of malar sulcus length). Length of OOL: less than or equal to 1/3× width of ocellus. Sculpture of vertex: granulate. Sculpture of occipital carina: largely simple, at most with sparse weak crenulae medially. Distance from occipital carina to orbital carina: at least 2× width of occipital carina. Shape of occipital carina: simply arcuate medially. Sculpture of occiput: with weak, small granulae. Sculpture of gena: granulose.

Mesosoma. Dorsal mesosoma in lateral view: convex. Sculpture of pronotal cervical area: with small (at most as large as crenulae on anterior edge of mesoscutum), well-defined foveae. Sculpture of pronotal lateral area: upper 1/3 granulose, lower 1/3 with transverse foveae, otherwise smooth. Netrion: present, smooth, well developed, sub-obovate. Notaulus: present, simple. Length of notaulus: approximately less than or equal to 1/3 of length of mesoscutum. Width of notaulus: narrow (notaulus width less than or equal to half the width of tegula). Sculpture of mesoscutum: weakly rugulose mixed with weak granulae. Sculpture of mesoscutellum: granulose. Mesoscutellar profile: elevated, anterior margin higher than posterior. Mesoscutellar shape: flat, not depressed. Lateral propodeal area: densely, finely rugulose. Shape of propodeal anterior spine: elongate, narrow, apex rounded. Sculpture of propodeum between anterior spines: smooth or largely smooth. Sculpture of ventral half of mesepisternum: weakly coriaceous. Sculpture of upper 1/4 of mesopleuron: densely longitudinally costate across half width. Metapleural sculpture: mainly smooth, lower third sparsely longitudinally carinate.

*Wings*. Stigmal vein: present, elongate, narrow. Campaniform sensilla at distal area of stigmal vein: present.

*Metasoma*. Shape of T1 horn: narrow, short. Sculpture of upper portion of T1 horn: longitudinally carinate. Sculpture of posterior portion of T1 horn: largely smooth, with sparse longitudinal carinae. Lateral carinae on T2: present, well-defined. Sculpture of T2: longitudinally costate, posterior margin smooth. Sculpture of T3: anterior two-thirds weakly longitudinally costate, posterior third weakly coriaceous mesally, otherwise densely granulose. Sculpture of S3–S6: S3 weakly granulose, S4–S6 weakly, finely coriaceous. S2 anterior carina: present, cristate, uninterrupted.

*Male*. Unknown.

##### Diagnosis.

This species is very similar to *Odontacolus markadicus*; however, *Odontacolus mot* differs from it by the larger body size (1.92 mm vs. 1.74 mm or less), the broader transverse sinuate carinae on the upper frons (in *Odontacolus markadicus* the sculpturing is completely absent or if present then the carinae are thin and straighter than in *Odontacolus mot*), and by the denser and less evenly distributed setae on the metasoma. Additionally, T2 in *Odontacolus mot* is more quadrate, while it is more elongate and less broad posteriorly in *Odontacolus markadicus*.

##### Etymology.

The name of this species is an arbitrary combination of letters and is used as a noun in apposition.

##### Link to distribution map.

82

##### Material Examined.

Holotype female: **INDIA:** Tamil Nadu St., Coimbatore, 7.XI.1979, Boucek, OSUC 239205 (deposited in BMNH).

##### Comments.

The holotype is in perfect condition. The differences between *Odontacolus mot* and *Odontacolus markadicus* could be associated with the difference in body size. However, since *Odontacolus mot* also differs in metasomal setation and sculpture, we have chosen to treat them as separate species until additional material of *Odontacolus mot* is available.

#### Odontacolus noyesi

Valerio & Austin sp. n.

urn:lsid:zoobank.org:act:2323FDC8-52FD-4358-8D00-3ED8A50F7449

urn:lsid:biosci.ohio-state.edu:osuc_concepts:254258

http://species-id.net/wiki/Odontacolus_noyesi

Figures 18 , 27 , 233–238 ; Morphbank ^83^

##### Description.

*Female*. Body length: 1.54 – 1.83 mm (n=2). Antenna color: completely yellow. Body color: mostly yellow, T1 horn and T4–T6 dark brown. Coxae color: yellow. Leg color (excluding coxae): yellow. Fore wing color: completely hyaline.

*Head*. Size of compound eye: reduced, approximately less than or equal to 1/3× height of head. Head shape in lateral view: lower head elongate and broad at mouth, head appearing elongate and somewhat thin. Sculpture of antennal scrobe: coriaceous throughout. Surface of torular triangle: flat. Development of central keel on frons: present, elongate (equal to or greater than 1/3× height of frons), but not reaching anterior ocellus. Sculpture on upper frons below anterior ocellus: with sparse, transverse costae mixed with weak, dense granulae. Sculpture of malar space: coriaceous throughout, without fan-like striae. Furrow at lateral portion of antennal scrobe: absent. Mesal surface of vertex: flat to weakly convex. Size of lateral ocelli: normal. Distance between lateral ocellus and occipital carina: 0.5–1.2× maximum ocellar diameter. Lagrimal: absent or minute. Length of OOL: less than or equal to 1/3× width of ocellus. Sculpture of vertex: granulate. Sculpture of occipital carina: largely simple, at most with sparse weak crenulae medially. Distance from occipital carina to orbital carina: at least 2× width of occipital carina. Shape of occipital carina: simply arcuate medially. Sculpture of occiput: with weak, small granulae. Sculpture of gena: granulose.

*Mesosoma*. Dorsal mesosoma in lateral view: convex. Sculpture of pronotal cervical area: with small (at most as large as crenulae on anterior edge of mesoscutum), well-defined foveae. Sculpture of pronotal lateral area: coriaceous. Netrion: present, smooth, well developed, sub-obovate. Notaulus: present, with crenulae that extend completely through depth of furrow. Length of notaulus: approximately less than or equal to 1/3 of length of mesoscutum. Width of notaulus: narrow (notaulus width less than or equal to half the width of tegula). Sculpture of mesoscutum: finely granulose. Sculpture of mesoscutellum: with weak, fine, granulate sculpture. Mesoscutellar profile: mainly flat, anterior and posterior edge at same height or nearly so. Mesoscutellar shape: flat, not depressed. Lateral propodeal area: densely, finely rugulose. Shape of propodeal anterior spine: short, broad, apex subtriangular. Sculpture of propodeum between anterior spines: smooth or largely smooth; longitudinally costate. Sculpture of ventral half of mesepisternum: weakly coriaceous. Sculpture of upper 1/4 of mesopleuron: densely longitudinally costate across entire width. Metapleural sculpture: mainly with weak coriaceous sculpture, lower 1/3 without longitudinal costae.

*Wings*. Stigmal vein: present, elongate, narrow. Campaniform sensilla at distal area of stigmal vein: present.

*Metasoma*. Shape of T1 horn: broad, short. Sculpture of upper portion of T1 horn: longitudinally carinate. Sculpture of posterior portion of T1 horn: longitudinally carinate. Lateral carinae on T2: present, poorly defined. Sculpture of T2: longitudinally costate on coriaceous background. Sculpture of T3: anterior half weakly longitudinally costate, otherwise coriaceous, microsculpture strongest medially. Sculpture of S3–S6: finely, weakly coriaceous. S2 anterior carina: present, cristate, uninterrupted.

*Male*. Unknown.

##### Diagnosis.

This is the only species of *Odontacolus* that has the compound eyes unusually small, approximately 1/3 of the height of the head.

##### Etymology.

This species is named after our friend and colleague, the chalcid specialist and great insect collector Dr John Noyes from the Natural History Museum, London. The epithet is a noun in the genitive case.

##### Link to distribution map.

84

##### Material examined.

Holotype female: **INDIA**: Karnataka St., Bannerghatta National Park, 5.XI.1979, Bouček & Noyes, OSUC 237932 (deposited in BMNH). *Paratype*: **INDONESIA**: 1 female, OSUC 237931 (CNCI).

##### Comments.

The holotype has the left legs separate and glued to the point. Otherwise it is in good condition. The paratype in perfect condition: it has the metasoma completely honey yellow and the remainder of the body, including the legs, yellow.

#### Odontacolus pintoi

Valerio & Austin sp. n.

urn:lsid:zoobank.org:act:E872D1A1-2C3C-4322-B08A-02957BA46B17

urn:lsid:biosci.ohio-state.edu:osuc_concepts:254267

http://species-id.net/wiki/Odontacolus_pintoi

Figures 20, 22 , 239-244 , 296 , 300, 302 , 318 , 323–324 ; Morphbank ^85^

##### Description.

*Female*. Body length: 1.06 – 1.25 mm (n=7). Antenna color: antennal clava dark brown, otherwise yellow. Body color: mostly dark brown, T1 (except dark brown horn) and anterior portion of T2 yellow. Coxae color: honey yellow. Leg color (excluding coxae): yellow. Fore wing color: basal half hyaline, otherwise weakly infuscate.

*Head*. Size of compound eye: approximately 1/2× height of head. Head shape in lateral view: lower head elongate and broad at mouth, head appearing elongate and somewhat thin. Sculpture of antennal scrobe: smooth throughout. Surface of torular triangle: slightly bulging. Development of central keel on frons: present, elongate (equal to or greater than 1/3× height of frons), but not reaching anterior ocellus. Sculpture on upper frons below anterior ocellus: granulose throughout. Sculpture of malar space: largely smooth, with sparse, weak, fan-like striae. Furrow at lateral portion of antennal scrobe: absent. Mesal surface of vertex: flat to weakly convex. Size of lateral ocelli: minute. Distance between lateral ocellus and occipital carina: greater than 1.5× maximum ocellar diameter. Lagrimal: absent or minute. Length of OOL: less than or equal to 1/3× width of ocellus. Sculpture of vertex: granulate. Sculpture of occipital carina: largely simple, at most with sparse weak crenulae medially. Distance from occipital carina to orbital carina: at least 2× width of occipital carina. Shape of occipital carina: simply arcuate medially. Sculpture of occiput: with weak, small granulae. Sculpture of gena: coriaceous dorsally, otherwise smooth.

*Mesosoma*. Dorsal mesosoma in lateral view: convex. Sculpture of pronotal cervical area: with small (at most as large as crenulae on anterior edge of mesoscutum), well-defined foveae. Sculpture of pronotal lateral area: transversely costate. Netrion: present, smooth, linear. Notaulus: present, simple. Length of notaulus: approximately less than or equal to 1/3 of length of mesoscutum. Width of notaulus: narrow (notaulus width less than or equal to half the width of tegula). Sculpture of mesoscutum: coriaceous. Sculpture of mesoscutellum: smooth or nearly so. Mesoscutellar profile: mainly flat, anterior and posterior edge at same height or nearly so. Mesoscutellar shape: flat, not depressed. Lateral propodeal area: sparsely transversely carinate. Shape of propodeal anterior spine: short, broad, apex subtriangular. Sculpture of propodeum between anterior spines: smooth or largely smooth. Sculpture of ventral half of mesepisternum: smooth or nearly so. Sculpture of upper 1/4 of mesopleuron: densely longitudinally costate across entire width. Metapleural sculpture: smooth.

*Wings*. Stigmal vein: present, short, narrow. Campaniform sensilla at distal area of stigmal vein: present.

*Metasoma*. Shape of T1 horn: narrow, elongate. Sculpture of upper portion of T1 horn: smooth. Sculpture of posterior portion of T1 horn: largely smooth, with sparse longitudinal carinae. Lateral carinae on T2: absent. Sculpture of T2: meson and posterior margin smooth, otherwise longitudinally costate. Sculpture of T3: sublaterally weakly longitudinally costate in anterior third, otherwise smooth. Sculpture of S3–S6: finely, weakly coriaceous. S2 anterior carina: present, rounded, uninterrupted.

*Male*. Body length: 1.27 – 1.30 mm (n= 3). Body color: antenna yellow as legs and T1, remainder of metasoma dark brown as dorsal mesosoma as head, remainder of body dark honey yellow. Sculpture of antennal scrobe: with weakly sinuate, fine, weak dorsoventral carinae, except area below anterior ocellus with granulate sculpture. Shape and size of anterior ocellus: minute, very round. Vertex posterior area sculpture: dense granulate sculpture. Occipital carina dorsal area: cristate, conspicuously present. Netrion: well-defined, suboval, broad longitudinal carinae present before netrion. Sculpture of mesepisternum: absent (smooth). Sculpture of pronotal lateral areas: with few, sinuate, transverse carinae. Length of fore wing stigmal vein: conspicuously elongate. Angle of stigmal vein in relation to anterior margin of fore wing: at an angle of approximately 45°. Sculpture of T2: mesal area smooth, remainder of tergum with weak longitudinal carinae.

##### Diagnosis.

*Odontacolus pintoi* can be distinguished from all other species that have an obscured netrion in combination with the elongate and broad ventral portion of the head (below the eyes in anterior view) (Fig. 243) by the mostly smooth gena (shiny, sometimes with large coriaceous sculpture present but restricted to upper 1/3), and the smooth antennal scrobe (except for the presence of a central keel).

##### Etymology.

This species is named after the trichogrammatid specialist Dr John Pinto, formerly of the University of California, Riverside. The epithet is a noun in the genitive case.

##### Link to distribution map.

86

##### Biology.

Specimens from South Australia have been reared from eggs of *Clubiona cycladata* Simon (Araneae: Clubionidae) under the bark of eucalypt trees (recorded as *Odontacolus* sp. in Austin 1984 & Austin 1985).

##### Material examined.

Holotype female: **AUSTRALIA**: WA, Fitzgerald River National Park, 33°41.6'S, 119°42.9'E, 17.X.2002, yellow pan trap, J. Pinto, OSUC 239098 (deposited in WAMP). *Paratypes*: (79 females, 4 males) **AUSTRALIA**: 54 females, 4 males, OSUC 239044–239054, 239064–239067, 239069, 239075–239076, 239090, 239097, 239103 (ANIC); OSUC 239085–239088 (BMNH); OSUC 238542, 239072, 239100–239102 (CNCI); OSUC 239099 (OSUC); OSUC 239070–239071, 239073–239074, 239096, 239106–239107, 239109–239115 (QDPC); OSUC 239055–239063, 239092–239095 (WINC). **NEW ZEALAND**: 8 females, OSUC 239082 (LUNZ); OSUC 239077–239081, 239083–239084 (NZAC). **NORFOLK ISLAND**: 17 females, OSUC 239031–239042, 239068, 239089, 239104–239105 (ANIC); OSUC 239091 (QDPC). *Other material*: **AUSTRALIA**: 1 female, OSUC 239043 (ANIC).

##### Comments.

The holotype is in perfect condition; the paratypes are for the most part in very good condition except for OSUC 239063 and OSUC 239074 which have the heads detached from body and glued to the point, and OSUC 239046 which is missing the metasoma. Some females have a different color pattern in that the whole body is dark brown with the anterior edge of T1 (except the horn), T2 and the legs yellow.

#### Odontacolus schlingeri

Valerio & Austin sp. n.

urn:lsid:zoobank.org:act:016D0F23-8713-48A2-AD18-6C9D83FA1DE4

urn:lsid:biosci.ohio-state.edu:osuc_concepts:254274

http://species-id.net/wiki/Odontacolus_schlingeri

Figures 13 , 245–250 ; Morphbank ^87^

##### Description.

*Female*. Body length: 1.47 mm (n=1). Antenna color: completely whitish yellow. Body color: head and mesosoma dark brown, head lighter in color than mesosoma, metasoma mostly whitish yellow, T1 dark honey yellow, T2 light honey yellow anteriorly. Coxae color: whitish yellow. Leg color (excluding coxae): whitish yellow. Fore wing color: completely hyaline.

*Head*. Size of compound eye: approximately 1/2× height of head. Head shape in lateral view: lower head elongate and broad at mouth, head appearing elongate and somewhat thin. Sculpture of antennal scrobe: weakly granulose throughout. Surface of torular triangle: flat. Development of central keel on frons: present, short (less than 1/3 of frons height). Sculpture on upper frons below anterior ocellus: granulose throughout. Sculpture of malar space: granulose throughout, without fan-like striae. Furrow at lateral portion of antennal scrobe: absent. Mesal surface of vertex: flat to weakly convex. Size of lateral ocelli: large. Distance between lateral ocellus and occipital carina: 0.5–1.2× maximum ocellar diameter. Lagrimal: absent or minute. Length of OOL: less than or equal to 1/3× width of ocellus. Sculpture of vertex: granulate. Sculpture of occipital carina: weakly crenulate throughout. Distance from occipital carina to orbital carina: slightly greater than width of occipital carina. Shape of occipital carina: weakly sinuate medially. Sculpture of occiput: with weak, small granulae. Sculpture of gena: with weak rugulose sculpture and granulate background sculpture.

*Mesosoma*. Dorsal mesosoma in lateral view: conspicuously flattened. Sculpture of pronotal lateral area: largely smooth, dorsal margin with dense, weak punctulae. Netrion: present, smooth, linear. Notaulus: absent. Length of notaulus: not applicable, notauli absent. Width of notaulus: not applicable, notauli absent. Sculpture of mesoscutum: weakly rugulose, carinae thin in shape. Sculpture of mesoscutellum: rugulose. Mesoscutellar profile: mainly flat, anterior and posterior edge at same height or nearly so. Mesoscutellar shape: flat, not depressed. Lateral propodeal area: longitudinally costate. Shape of propodeal anterior spine: short, broad, apex rounded. Sculpture of propodeum between anterior spines: smooth or largely smooth. Sculpture of ventral half of mesepisternum: smooth or nearly so. Sculpture of upper 1/4 of mesopleuron: smooth. Metapleural sculpture: smooth.

*Wings*. Stigmal vein: present, elongate, narrow. Campaniform sensilla at distal area of stigmal vein: present.

*Metasoma*. Shape of T1 horn: broad, short. Sculpture of upper portion of T1 horn: longitudinally carinate. Sculpture of posterior portion of T1 horn: smooth. Lateral carinae on T2: absent. Sculpture of T2: weakly coriaceous. Sculpture of T3: coriaceous. Sculpture of S3–S6: finely, weakly coriaceous. S2 anterior carina: absent.

*Male*. Unknown.

##### Diagnosis.

This species can be distinguished from all other *Odontacolus* species lacking notauli by the conspicuous dorsoventrally depressed mesosoma.

##### Etymology.

This species is named after the dipteran expert Dr Evert Schlinger, collector of this species. The epithet is a noun in the genitive case.

##### Link to distribution map.

88

##### Material examined.

Holotype female: **FIJI**: Central Div., Rewa Prov., Viti Levu Isl., 3.8km N Veisari Settlement, MT2, logging road to Waivudawa, 18.079°S, 178.363°E, 300m, 12.XII–3.I.2003, Malaise trap, Schlinger & Tokota’a, FBA104329 (deposited in BPBM).

##### Comments.

The holotype is in perfect condition.

#### Odontacolus sharkeyi

Valerio & Austin sp. n.

urn:lsid:zoobank.org:act:F72B6124-C01B-444F-BC75-43323B09FA8F

urn:lsid:biosci.ohio-state.edu:osuc_concepts:275238

http://species-id.net/wiki/Odontacolus_sharkeyi

Figures 46 , 251–256 ; Morphbank ^89^

##### Description.

*Female*. Body length: 1.28 – 1.60 mm (n=9). Antenna color: A1 lighter in color than remainder of dark brown antennomeres. Body color: completely dark brown. Coxae color: dark brown. Leg color (excluding coxae): yellow. Fore wing color: completely hyaline.

*Head*. Size of compound eye: approximately 1/2× height of head. Head shape in lateral view: lower head elongate and broad at mouth, head appearing elongate and somewhat thin. Sculpture of antennal scrobe: largely smooth ventrally, dorsally with sinuate, transverse ridges.Surface of torular triangle: flat. Development of central keel on frons: present, elongate, reaching anterior ocellus. Sculpture on upper frons below anterior ocellus: with sparse, transverse costae mixed with weak, dense granulae. Sculpture of malar space: with fan-like striae, striae extending into antennal scrobe. Furrow at lateral portion of antennal scrobe: present. Mesal surface of vertex: flat to weakly convex. Size of lateral ocelli: large. Distance between lateral ocellus and occipital carina: 0.5–1.2× maximum ocellar diameter. Lagrimal: absent or minute. Length of OOL: less than or equal to 1/3× width of ocellus. Sculpture of vertex: granulate. Sculpture of occipital carina: weakly crenulate throughout. Distance from occipital carina to orbital carina: at least 2× width of occipital carina. Shape of occipital carina: simply arcuate medially. Sculpture of occiput: with weakly rugulo aciculate sculpture. Sculpture of gena: granulose.

*Mesosoma*. Dorsal mesosoma in lateral view: convex. Sculpture of pronotal cervical area: with small (at most as large as crenulae on anterior edge of mesoscutum), well-defined foveae. Sculpture of pronotal lateral area: upper 1/3 granulose, lower 1/3 with transverse foveae, otherwise smooth. Netrion: present, smooth, linear. Notaulus: present, simple. Length of notaulus: approximately less than or equal to 1/3 of length of mesoscutum. Width of notaulus: narrow (notaulus width less than or equal to half the width of tegula). Sculpture of mesoscutum: weakly rugulose mixed with weak granulae. Sculpture of mesoscutellum: granulose. Mesoscutellar profile: mainly flat, anterior and posterior edge at same height or nearly so. Mesoscutellar shape: flat, not depressed. Lateral propodeal area: coarsely rugulose. Shape of propodeal anterior spine: elongate, narrow, apex rounded. Sculpture of propodeum between anterior spines: smooth or largely smooth. Sculpture of ventral half of mesepisternum: smooth or nearly so. Sculpture of upper 1/4 of mesopleuron: densely longitudinally costate across half width. Metapleural sculpture: midtransverse area smooth, otherwise with cristate, longitudinal carinae.

*Wings*. Stigmal vein: present, elongate, narrow. Campaniform sensilla at distal area of stigmal vein: present.

*Metasoma*. Shape of T1 horn: broad, short. Sculpture of upper portion of T1 horn: longitudinally carinate. Sculpture of posterior portion of T1 horn: smooth. Lateral carinae on T2: present, poorly defined. Sculpture of T2: largely weakly coriaceous mixed with longitudinal costae, meson coriaceous. Sculpture of T3: anterior third weakly costate sublaterally, weakly coriaceous mesally, otherwise granulose. Sculpture of S3–S6: S3 weakly granulose, S4–S6 weakly, finely coriaceous. S2 anterior carina: present, cristate, uninterrupted.

*Male*. Unknown.

##### Diagnosis.

This is the only known species to have a furrow at the lateral areas of the antennal scrobes in combination with a completely dark brown body.

##### Etymology.

This species is named after our friend and colleague, hymenopterist Dr Mike Sharkey from the University of Kentucky. The epithet is a noun in the genitive case.

##### Link to distribution map.

90

##### Material examined.

Holotype female: **THAILAND**: Chanthaburi Prov., Prabad Unit, 120m to bridge, T3957, Khao Khitchakut National Park, 12°49.15'N, 102°07.13'E, 219m, 16.X–23.X.2008, Malaise trap, Sutthida & Charoenchai, OSUC 321869 (deposited in QSBG). *Paratypes*: **THAILAND**: 26 females, OSUC 339587 (CNCI); OSUC 339588, 339590–339592, 339598, 339600–339601, 339604–339605, 339607, 374180 (OSUC); OSUC 247710, 250612, 251977, 261748, 321867–321868, 321870–321871, 321873, 321887, 339577, 339579, 339586 (QSBG); OSUC 336787 (WINC).

##### Comments.

The holotype is in perfect condition except for the left antenna which is detached from the body but glued to the point; the paratype specimens are in good condition.

#### Odontacolus spinosus

(Dodd)

urn:lsid:zoobank.org:act:87C08D6D-E3EC-4FB0-B3B6-EB60BC0CC15A

urn:lsid:biosci.ohio-state.edu:osuc_concepts:4951

http://species-id.net/wiki/Odontacolus_spinosus

Figures 47 , 257–262 ; Morphbank ^91^

Ceratobaeoides spinosus Dodd 1914b: 125 (original description); Kieffer 1926: 273, 274 (description, keyed).
Odontacolus spinosus (Dodd): Austin 1981: 89 (generic transfer, type information).
Ceratobaeoides longiceps Dodd 1913: 338 (original description. Preoccupied by *Odontacolus longiceps* Kieffer 1910a); Dodd 1914a: 66 (description); Kieffer 1926: 273, 274 (description, keyed).
Odontacolus spinosus urn:lsid:zoobank.org:act:91260D13-006C-4F64-B6EF-15C984A4EB1F
Odontacolus spinosus urn:lsid:biosci.ohio-state.edu:osuc_concepts:9360
Odontacolus doddi Austin 1981: 88 (replacement name, generic transfer, type information). **Syn. n.**
Odontacolus spinosus urn:lsid:zoobank.org:act:E815E65F-BD05-4045-BEF3-4FFF3445D1CD
Odontacolus spinosus urn:lsid:biosci.ohio-state.edu:osuc_concepts:4946

##### Description.

*Female*. Body length: 1.09 – 1.63 mm (n=20). Antenna color: antennal clava dark brown, otherwise yellow; distal 2/3 of antennal clava dark brown, otherwise yellow. Body color: mainly yellow, except for occiput, mesoscutum midanterior and lateral areas and mesepisternum lower area dark brown, T1 horn black, and T4–T6 honey yellow. Coxae color: yellow. Leg color (excluding coxae): yellow. Fore wing color: completely hyaline.

*Head*. Size of compound eye: approximately 1/2× height of head. Head shape in lateral view: lower head elongate and broad at mouth, head appearing elongate and somewhat thin. Sculpture of antennal scrobe: weakly rugulose throughout. Surface of torular triangle: flat. Development of central keel on frons: present, elongate (equal to or greater than 1/3× height of frons), but not reaching anterior ocellus. Sculpture on upper frons below anterior ocellus: with sparse, transverse costae mixed with weak, dense granulae. Sculpture of malar space: weakly rugulose throughout, without fan-like striae. Furrow at lateral portion of antennal scrobe: absent. Mesal surface of vertex: flat to weakly convex. Size of lateral ocelli: small. Distance between lateral ocellus and occipital carina: 0.5–1.2× maximum ocellar diameter. Lagrimal: absent or minute. Length of OOL: less than or equal to 1/3× width of ocellus. Sculpture of vertex: granulate. Sculpture of occipital carina: weakly crenulate throughout. Distance from occipital carina to orbital carina: at least 2× width of occipital carina. Shape of occipital carina: simply arcuate medially. Sculpture of occiput: with weakly rugulo aciculate sculpture. Sculpture of gena: granulose.

*Mesosoma*. Dorsal mesosoma in lateral view: convex. Sculpture of pronotal cervical area: with small (at most as large as crenulae on anterior edge of mesoscutum), well-defined foveae. Sculpture of pronotal lateral area: coriaceous. Netrion: present, smooth, linear. Notaulus: present, simple. Length of notaulus: approximately less than or equal to 1/3 of length of mesoscutum. Width of notaulus: narrow (notaulus width less than or equal to half the width of tegula). Sculpture of mesoscutum: weakly rugulose mixed with weak granulae. Sculpture of mesoscutellum: weakly rugulose mixed with granulate sculpture. Mesoscutellar profile: elevated, anterior margin higher than posterior. Mesoscutellar shape: flat, not depressed. Lateral propodeal area: densely, finely rugulose. Shape of propodeal anterior spine: elongate, narrow, apex rounded. Sculpture of propodeum between anterior spines: rugulose throughout. Sculpture of ventral half of mesepisternum: weakly, finely coriaceous. Sculpture of upper 1/4 of mesopleuron: densely longitudinally costate across entire width. Metapleural sculpture: sparsely longitudinally costate.

*Wings*. Stigmal vein: present, elongate, narrow. Campaniform sensilla at distal area of stigmal vein: present.

*Metasoma*. Shape of T1 horn: broad, short. Sculpture of upper portion of T1 horn: longitudinally carinate. Sculpture of posterior portion of T1 horn: longitudinally carinate. Lateral carinae on T2: present, well-defined. Sculpture of T2: longitudinally costate on coriaceous background. Sculpture of T3: anterior half weakly, longitudinally costate, coriaceous mesally, otherwise weakly coriaceous. Sculpture of S3–S6: finely, weakly coriaceous. S2 anterior carina: present, cristate, uninterrupted.

*Male*. Unknown.

##### Diagnosis.

*Odontacolus spinosus* can be separated from all other Australian *Odontacolus* species by the short and unsculptured notauli in combination with a well-defined netrion, central keel present on the frons, sculptured antennal scrobes, small lateral ocelli, and the long distance between the lateral ocellus and the occipital carina, approximately 1.2× the ocellus diameter.

##### Link to distribution map.

92

##### Material examined.

Holotype female, *Ceratobaeoides longiceps*: **AUSTRALIA**: QLD, among undergrowth, Brisbane, 26.IV.1913, H. Hacker, QMBA HY1631 (deposited in QMBA). Holotype female, *Ceratobaeoides spinosus*: **AUSTRALIA**: QLD, forest, Childers, 2.VII.1914, sweeping, A. P. Dodd, SAMA DB 32-001530 (deposited in SAMA). *Other material*: **AUSTRALIA**: 31 females, OSUC 239167, 239173, 239180, 239182 (ANIC); OSUC 239153, 239165–239166, 239174, 239177, 239184, 339575 (CNCI); OSUC 239188 (OSUC); OSUC 238011, 239015, 239154, 239164, 239168–239172, 239175–239176, 239179, 239183, 239185–239186 (QDPC); OSUC 239017 (QMBA); UCRC ENT 171076–171077 (UCRC); OSUC 239189 (WINC).

##### Comments.

The holotype of *Odontacolus spinosus* has the head and most of the wings detached from the body; the head and first pair of legs are glued to the point; the remainder of the legs has the distal tarsomeres missing. The holotype of *Odontacolus doddi* is slide mounted and the specimen was partly destroyed in the process.

The following color variations occur in this species: the body ranges from yellow with the tips of the propodeal spines and T1 horn dark brown to mostly dark brown with the posterior area of the mesoscutum and mesoscutellum light yellow. The occipital carina of the specimens OSUC 239180 and OSUC 239173 is present but very fine which renders it difficult to see; in all other specimens it is well developed. The sculpture also varies between the anterior propodeal spines, from being completely smooth to completely punctate in some specimens with darker color (i.e. OSUC 239165, 265167, 239170–239172). The sculpture of the mesal portion of T2 varies from in the development of the costae. In any case there is always coriaceous background sculpture.

#### Odontacolus veroae

Valerio & Austin sp. n.

urn:lsid:zoobank.org:act:8C19EABF-FCA1-498D-989C-6E918DB53408

urn:lsid:biosci.ohio-state.edu:osuc_concepts:254273

http://species-id.net/wiki/Odontacolus_veroae

Figures 14, 15 , 60 , 263–268 ; Morphbank ^93^

##### Description.

*Female*. Body length: 1.47 – 1.71 mm (n=3). Antenna color: completely yellow. Body color: completely dark brown. Coxae color: yellow. Leg color (excluding coxae): yellow. Fore wing color: slightly infuscate throughout.

*Head*. Size of compound eye: approximately 1/2× height of head. Head shape in lateral view: lower head elongate and broad at mouth, head appearing elongate and somewhat thin. Sculpture of antennal scrobe: weakly granulose throughout. Surface of torular triangle: flat. Development of central keel on frons: present, elongate (equal to or greater than 1/3× height of frons), but not reaching anterior ocellus. Sculpture on upper frons below anterior ocellus: granulose throughout. Sculpture of malar space: granulose throughout, without fan-like striae. Furrow at lateral portion of antennal scrobe: absent. Mesal surface of vertex: flat to weakly convex. Size of lateral ocelli: large. Distance between lateral ocellus and occipital carina: 0.5–1.2× maximum ocellar diameter. Lagrimal: absent or minute. Length of OOL: less than or equal to 1/3× width of ocellus. Sculpture of vertex: granulate. Sculpture of occipital carina: largely simple, at most with sparse weak crenulae medially. Distance from occipital carina to orbital carina: contiguous or nearly so, subequal to width of occipital carina. Shape of occipital carina: simply arcuate medially. Sculpture of occiput: uncertain, with weakly rugulo aciculate sculpture. Sculpture of gena: granulose.

*Mesosoma*. Dorsal mesosoma in lateral view: convex. Sculpture of pronotal cervical area: with small (at most as large as crenulae on anterior edge of mesoscutum), well-defined foveae. Sculpture of pronotal lateral area: largely smooth, dorsal margin with dense, weak punctulae. Netrion: present, smooth, linear. Notaulus: absent. Length of notaulus: not applicable, notauli absent. Width of notaulus: not applicable, notauli absent. Sculpture of mesoscutum: rugulose, mixed with weak granulae. Sculpture of mesoscutellum: granulose. Mesoscutellar profile: mainly flat, anterior and posterior edge at same height or nearly so. Mesoscutellar shape: flat, not depressed. Lateral propodeal area: densely, finely rugulose. Shape of propodeal anterior spine: short, broad, apex subtriangular. Sculpture of propodeum between anterior spines: smooth or largely smooth. Sculpture of ventral half of mesepisternum: weakly coriaceous. Sculpture of upper 1/4 of mesopleuron: densely longitudinally costate across entire width. Metapleural sculpture: mainly with weak coriaceous sculpture, lower 1/3 without longitudinal costae.

*Wings*. Stigmal vein: present, elongate, narrow. Campaniform sensilla at distal area of stigmal vein: present.

*Metasoma*. Shape of T1 horn: broad, short. Sculpture of upper portion of T1 horn: longitudinally carinate. Sculpture of posterior portion of T1 horn: smooth. Lateral carinae on T2: absent. Sculpture of T2: largely weakly coriaceous mixed with longitudinal costae, meson coriaceous. Sculpture of T3: coriaceous. Sculpture of S3–S6: finely, weakly coriaceous. S2 anterior carina: absent.

*Male*. Unknown.

##### Diagnosis.

The species *Odontacolus veroae* is very similar to *Odontacolus irwini* but can be distinguished from it by the cicatrose sculpture of the posterior sublateral portions of the mesoscutum, the remainder of the mesoscutum having dense, weak, granulate sculpture; and the large body size (1.47–1.71 mm) of *Odontacolus irwini*. Additionally, within the Fijian species, *Odontacolus irwini* can be separated from *Odontacolus heratyi* by the larger space between the occipital carina and the ocular carina, and from *Odontacolus schlingeri* by the convex mesosoma.

##### Etymology.

This species is named after Veronica Valerio, sister of the first author. The epithet is a noun in the genitive case.

##### Link to distribution map.

94

##### Material examined.

Holotype female: **FIJI**: Northern Div., Cakaudrove Prov., Taveuni Isl., FJ-9, Devo Forest Reserve, 16°50'S, 179°59'E, 800m, 3.I–10.I.2003, malaise trap, M. Irwin, E. Schlinger & M. Tokota’a, FBA042201 (deposited in BPBM). *Paratypes*: **FIJI**: 13 females, FBA030104, FBA030106, FBA030114, FBA081512, FBA081515, FBA084170 (BPBM); FBA021043, FBA039550, FBA049221 (CNCI); FBA014430, FBA104362 (FNIC); FBA081513, FBA182160 (OSUC).

##### Comments.

The holotype has all but one leg missing, but otherwise is in perfect condition; the paratypes are in good condition. The color of the metasoma varies from completely dark brown (matching the color of the head and mesosoma) to being conspicuously lighter than the head and mesosoma. The color of the antenna may vary from yellow to brown.

#### Odontacolus wallacei

Valerio & Austin sp. n.

urn:lsid:zoobank.org:act:122C0426-04B2-4079-87AB-A532A6C126EC

urn:lsid:biosci.ohio-state.edu:osuc_concepts:254261

http://species-id.net/wiki/Odontacolus_wallacei

Figures 28 , 32 , 37–38 , 269–274 , 301 , 308 , 314 , 325–326 ; Morphbank ^95^

##### Description.

*Female*. Body length: 1.01 – 1.64 mm (n=9). Antenna color: A1 yellow, otherwise dark brown. Body color: head and mesosoma yellow, metasoma honey yellow with T1 horn dark brown, T3 whitish yellow, T4–T6 darker in color than T1–T2. Coxae color: yellow. Leg color (excluding coxae): yellow. Fore wing color: slightly infuscate throughout.

*Head*. Size of compound eye: approximately 1/2× height of head. Head shape in lateral view: lower head elongate and broad at mouth, head appearing elongate and somewhat thin. Sculpture of antennal scrobe: weakly coriaceous throughout. Surface of torular triangle: flat. Development of central keel on frons: completely absent. Sculpture on upper frons below anterior ocellus: coriaceous throughout. Sculpture of malar space: coriaceous throughout, without fan-like striae. Furrow at lateral portion of antennal scrobe: absent. Mesal surface of vertex: flat to weakly convex. Size of lateral ocelli: minute. Distance between lateral ocellus and occipital carina: greater than 1.5× maximum ocellar diameter. Lagrimal: absent or minute. Length of OOL: less than or equal to 1/3× width of ocellus. Sculpture of vertex: granulate. Sculpture of occipital carina: largely simple, at most with sparse weak crenulae medially. Distance from occipital carina to orbital carina: at least 2× width of occipital carina. Shape of occipital carina: simply arcuate medially. Sculpture of occiput: with weak, small granulae. Sculpture of gena: granulose.

*Mesosoma*. Dorsal mesosoma in lateral view: convex. Sculpture of pronotal cervical area: with small (at most as large as crenulae on anterior edge of mesoscutum), well-defined foveae. Sculpture of pronotal lateral area: largely smooth, dorsal margin with dense, weak punctulae. Netrion: present, smooth, well developed, sub-obovate. Notaulus: present, simple. Length of notaulus: approximately less than or equal to 1/3 of length of mesoscutum. Width of notaulus: narrow (notaulus width less than or equal to half the width of tegula). Sculpture of mesoscutum: finely granulose. Sculpture of mesoscutellum: with weak, fine, granulate sculpture. Mesoscutellar profile: mainly flat, anterior and posterior edge at same height or nearly so. Mesoscutellar shape: flat, not depressed. Lateral propodeal area: sparsely transversely carinate. Shape of propodeal anterior spine: elongate, narrow, apex rounded. Sculpture of propodeum between anterior spines: smooth or largely smooth. Sculpture of ventral half of mesepisternum: weakly coriaceous. Sculpture of upper 1/4 of mesopleuron: with sparse, broad, smooth costate sculpture reaching just half of its width. Metapleural sculpture: mainly with weak coriaceous sculpture, lower 1/3 with sparse longitudinal carinae.

*Wings*. Stigmal vein: present, elongate, narrow. Campaniform sensilla at distal area of stigmal vein: present.

*Metasoma*. Shape of T1 horn: narrow, elongate. Sculpture of upper portion of T1 horn: longitudinally carinate. Sculpture of posterior portion of T1 horn: longitudinally carinate. Lateral carinae on T2: present, poorly defined. Sculpture of T2: anterior 2/3 longitudinally costate, otherwise coriaceous. Sculpture of T3: coriaceous. Sculpture of S3–S6: finely, weakly coriaceous. S2 anterior carina: present, cristate, interrupted medially.

*Male*. Body length: 1.53 mm (n=1). Body color: yellow except anteromedian area of mesoscutum trough anterior 2/3 of its length honey-yellow. Sculpture of antennal scrobe: mainly smooth medially throughout height, remainder with coriaceous sculpture. Shape and size of anterior ocellus: large, round. Vertex posterior area sculpture: densely granulose. Occipital carina dorsal area: cristate, conspicuously present. Netrion: well-defined, suboval. Sculpture of mesepisternum: absent (smooth). Sculpture of pronotal lateral areas: with dense, thin longitudinal carinae. Length of fore wing stigmal vein: conspicuously elongate. Angle of stigmal vein in relation to anterior margin of fore wing: at an angle of approximately 45° with respect to anterior margin of wing. Sculpture of T2: mesal area with weak, longitudinal carinae, sublateral areas with weak longitudinal carinae mixed with coriaceous sculpture.

##### Diagnosis.

Along with *Odontacolus lamarcki* this is the only other species without a central keel on the frons in combination with a well-defined netrion, and the occipital carina being separated from orbital carina. The species *Odontacolus wallacei* has the occipital carina separated from the lateral ocellus by approximately equal to or greater than 1.2× which contrasts with the much shorter distance present on *Odontacolus lamarcki* (of approximately less than or equal to 0.6× ocellus diameter).

##### Etymology.

This species is named after the great English naturalist Alfred Russel Wallace: long live his legacy! The epithet is a noun in the genitive case.

##### Link to distribution map.

96

##### Material examined.

Holotype female: **INDONESIA**: Sulawesi Utara Prov., Kotamobagu, 1300m, V-1985, Malaise trap, J. S. Noyes, OSUC 238001 (deposited in BMNH). *Paratypes*: (27 females, 1 male) **AUSTRALIA**: 11 females, OSUC 239018–239022 (ANIC); OSUC 239023 (CNCI); OSUC 239014 (QDPC); OSUC 239016 (QMBA); OSUC 239024–239026 (WINC). **INDONESIA**: 8 females, OSUC 238002, 238006, 238012 (CNCI); OSUC 148683–148684, 238003–238005 (WINC). **MALAWI**: 1 male, OSUC 238013 (BMNH). **PAPUA NEW GUINEA**: 8 females, OSUC 238007–238010, 239027, 239030 (CNCI); OSUC 239028–239029 (OSUC).

##### Comments.

The holotype generally is in good condition: the left front and hind legs are glued to the point, and the right hind leg is covered in glue. The metasomal color pattern varies between specimens from almost completely yellow (i.e. OSUC 238004) to being mostly honey yellow with whitish areas on T3 and dark brown T4–T6 (i.e. OSUC 238001). In some specimens (i.e. OSUC 239020) the upper area of the T1 horn is dark brown with the remainder of the metasoma yellow, and the mesoscutum (which is normally light yellow) being a honey yellow color mesally.

#### Odontacolus whitfieldi

Valerio & Austin sp. n.

urn:lsid:zoobank.org:act:A81CB2CA-F023-4392-AB4E-81C6BA9BB7B6

urn:lsid:biosci.ohio-state.edu:osuc_concepts:278397

http://species-id.net/wiki/Odontacolus_whitfieldi

Figures 275–280 , 304 , 311 , 315 , 327–328 ; Morphbank ^97^

##### Description.

*Female*. Body length: 1.35 – 1.97 mm (n=9). Antenna color: completely yellow. Body color: completely dark brown. Coxae color: yellow. Leg color (excluding coxae): yellow. Fore wing color: slightly infuscate throughout.

*Head*. Size of compound eye: approximately 1/2× height of head. Head shape in lateral view: lower head elongate and broad at mouth, head appearing elongate and somewhat thin. Sculpture of antennal scrobe: largely smooth ventrally, dorsally with sinuate, transverse ridges.Surface of torular triangle: slightly bulging. Development of central keel on frons: present, elongate (equal to or greater than 1/3× height of frons), but not reaching anterior ocellus. Sculpture on upper frons below anterior ocellus: with sparse, transverse costae mixed with weak, dense granulae. Sculpture of malar space: weakly rugulose throughout, without fan-like striae. Furrow at lateral portion of antennal scrobe: absent. Mesal surface of vertex: flat to weakly convex. Size of lateral ocelli: normal. Distance between lateral ocellus and occipital carina: 0.5–1.2× maximum ocellar diameter. Lagrimal: absent or minute. Length of OOL: less than or equal to 1/3× width of ocellus. Sculpture of vertex: granulate. Sculpture of occipital carina: weakly crenulate throughout. Distance from occipital carina to orbital carina: at least 2× width of occipital carina; slightly greater than width of occipital carina. Shape of occipital carina: simply arcuate medially. Sculpture of occiput: with weak, small granulae. Sculpture of gena: granulose.

*Mesosoma*. Dorsal mesosoma in lateral view: convex. Sculpture of pronotal cervical area: with small (at most as large as crenulae on anterior edge of mesoscutum), well-defined foveae. Sculpture of pronotal lateral area: coriaceous. Netrion: present, smooth, well developed, sub-obovate. Notaulus: present, simple. Length of notaulus: approximately less than or equal to 1/3 of length of mesoscutum. Width of notaulus: narrow (notaulus width less than or equal to half the width of tegula). Sculpture of mesoscutum: weakly rugulose mixed with weak granulae. Sculpture of mesoscutellum: granulose. Mesoscutellar profile: elevated, anterior margin higher than posterior. Mesoscutellar shape: flat, not depressed. propodeal area: densely, finely rugulose. Shape of propodeal anterior spine: elongate, narrow, apex rounded. Sculpture of propodeum between anterior spines: smooth or largely smooth. Sculpture of ventral half of mesepisternum: smooth or nearly so. Sculpture of upper 1/4 of mesopleuron: with sparse, broad, smooth costate sculpture reaching just half of its width. Metapleural sculpture: largely smooth except lower half with longitudinal carinae.

*Wings*. Stigmal vein: present, elongate, narrow. Campaniform sensilla at distal area of stigmal vein: present.

*Metasoma*. Shape of T1 horn: broad, short. Sculpture of upper portion of T1 horn: longitudinally carinate. Sculpture of posterior portion of T1 horn: largely smooth, with sparse longitudinal carinae. Lateral carinae on T2: present, well-defined. Sculpture of T2: longitudinally costate on coriaceous background. Sculpture of T3: anterior third weakly longitudinally costate, otherwise coriaceous. Sculpture of S3–S6: S3 weakly granulose, S4–S6 weakly, finely coriaceous. S2 anterior carina: present, cristate, uninterrupted.

*Male*. Body length: 1.29 – 1.37 mm (n=5). Body color: antenna yellow as legs, head, mesoscutum, mesoscutellum and metasoma dark brown, remainder of body light honey-yellow. Sculpture of antennal scrobe: lower mesal area smooth, remainder with weak coriaceous sculpture mixed with somewhat sinuate carinae (especially at upper 1/5), except area below anterior ocellus with granulose sculpture. Shape and size of anterior ocellus: small, round. Vertex posterior area sculpture: with dense, small granulae. Occipital carina dorsal area: cristate, conspicuously present. Netrion: well-defined, suboval. Sculpture of mesepisternum: with few, thin, transverse carinae. Sculpture of pronotal lateral areas: with few, thin transverse carinae, otherwise mainly smooth. Length of fore wing stigmal vein: conspicuously elongate. Angle of stigmal vein in relation to anterior margin of fore wing: at an angle of approximately 45°. Sculpture of T2: mostly smooth, lateral areas with few sparse longitudinal carinae.

##### Diagnosis.

*Odontacolus whitfieldi* is very similar to *Odontacolus kiau* but the former has a complete S2 anterior carina; *Odontacolus kiau* lacks this carina. Thesespecies belong to a group that have short and smooth notauli, a well-defined netrion, a central keel present on the frons and sculptured antennal scrobes always with transverse costae; within this group *Odontacolus mayri* can be separated from *Odontacolus whitfieldi* and *Odontacolus kiau* by its completely yellow body and the weakly granulose sculpture of the mesoscutum.

##### Etymology.

This species is named after our friend and colleague, the microgastrine systematist Dr Jim Whitfield at the University of Illinois. The epithet is a noun in the genitive case.

##### Link to distribution map.

98

##### Material examined.

Holotype female: **INDONESIA**: Sulawesi Utara Prov., Toraut, forest edge, Bogani Nani Wartabone (Dumoga-Bone) National Park, 15.VI–20.VI.1985, Malaise trap, OSUC 239160 (deposited in CNCI). *Paratypes*: (43 females, 5 males) **CHINA**: 13 females, 5 males, UCRC ENT 171075, 171078–171094 (UCRC). **INDIA**: 3 females, OSUC 321899 (BMNH); OSUC 238793, 238806 (CNCI). **INDONESIA**: 3 females, OSUC 238423, 238426, 239181 (CNCI). **MALAYSIA**: 5 females, OSUC 239157, 239162, 239178 (CNCI); OSUC 239187 (OSUC); OSUC 239161 (WINC). **THAILAND**: 18 females, OSUC 239156 (BMNH); OSUC 261747 (CNCI); OSUC 250856, 261746, 266234, 280625 (OSUC); OSUC 239163, 247709, 250610, 250859, 321866, 321875, 339578, 339580, 339584–339585, 339593, 339602 (QSBG). **VIETNAM**: 1 female, OSUC 278520 (RMNH).

##### Comments.

Holotype specimen is in perfect condition. Most of paratype specimens are in good condition.

#### Odontacolus zborowskii

Valerio & Austin sp. n.

urn:lsid:zoobank.org:act:182717E1-B89D-494B-9708-7056A17AA949

urn:lsid:biosci.ohio-state.edu:osuc_concepts:254266

http://species-id.net/wiki/Odontacolus_zborowskii

Figures 19 , 62 , 281–286 , 293, 297 , 309 , 329–330 ; Morphbank ^99^

##### Description.

*Female*. Body length: 1.45 – 1.52 mm (n=6). Antenna color: completely yellow. Body color: mostly yellow, mesopleuron, metapleuron and posterior area of T1–T3 dark brown. Coxae color: yellow. Leg color (excluding coxae): yellow. Fore wing color: largely hyaline, with transverse infumate band below stigma vein.

*Head*. Size of compound eye: approximately 1/2× height of head. Head shape in lateral view: lower head moderately short and strongly narrowed towards mouth, head appearing short and broad. Sculpture of antennal scrobe: with weak, sinuate, transverse ridges throughout. Surface of torular triangle: slightly bulging. Development of central keel on frons: present, short (less than 1/3 of frons height); present, elongate, reaching anterior ocellus. Sculpture on upper frons below anterior ocellus: covered by sinuate, transverse, fine costae. Sculpture of malar space: with fan-like striae, striae not extending into antennal scrobe. Furrow at lateral portion of antennal scrobe: absent. Mesal surface of vertex: flat to weakly convex. Size of lateral ocelli: small. Distance between lateral ocellus and occipital carina: greater than 1.5× maximum ocellar diameter. Lagrimal: absent or minute. Length of OOL: less than or equal to 1/3× width of ocellus. Sculpture of vertex: with sinuate, transverse, fine ridges. Sculpture of occipital carina: largely simple, at most with sparse weak crenulae medially. Distance from occipital carina to orbital carina: at least 2× width of occipital carina. Shape of occipital carina: weakly sinuate medially. Sculpture of occiput: with dense, fine transverse costae across its width. Sculpture of gena: with sinuate dorsoventral costae.

*Mesosoma*. Dorsal mesosoma in lateral view: convex. Sculpture of pronotal cervical area: with small (at most as large as crenulae on anterior edge of mesoscutum), well-defined foveae. Sculpture of pronotal lateral area: transversely costate. Netrion: absent, obscured by longitudinal sculpture of lateral pronotum. Notaulus: present, with low crenulae that do not extend through depth of furrow. Length of notaulus: approximately equal to or greater than 2/3 of length of mesoscutum. Width of notaulus: distinctly widened (notaulus width greater than half the width of tegula). Sculpture of mesoscutum: weakly rugulose, carinae somewhat broad in shape. Sculpture of mesoscutellum: anterior 1/3 with weak granulae, otherwise with rugulae. Mesoscutellar profile: elevated, anterior margin higher than posterior. Mesoscutellar shape: flat, not depressed. Lateral propodeal area: densely transversely carinate. Shape of propodeal anterior spine: elongate, narrow, apex rounded. Sculpture of propodeum between anterior spines: longitudinally costate. Sculpture of ventral half of mesepisternum: longitudinally costate. Sculpture of upper 1/4 of mesopleuron: densely longitudinally costate across entire width. Metapleural sculpture: midtransverse area smooth, otherwise with cristate, longitudinal carinae.

*Wings*. Stigmal vein: present, short, broad. Campaniform sensilla at distal area of stigmal vein: present.

*Metasoma*. Shape of T1 horn: narrow, elongate. Sculpture of upper portion of T1 horn: longitudinally carinate. Sculpture of posterior portion of T1 horn: longitudinally carinate. Lateral carinae on T2: absent. Sculpture of T2: anterior fourth longitudinally costate, otherwise smooth. Sculpture of T3: weakly coriaceous. Sculpture of S3–S6: finely, weakly coriaceous. S2 anterior carina: present, rounded, uninterrupted.

*Male*. Body length: 1.39 – 1.58 mm (n= 3). Body color: antenna light yellow as metasoma and legs, head honey-yellow; mesosoma dark brown; fore wing with a conspicuous dark band just before stigmal vein, remainder of fore wing lightly infuscate. Sculpture of antennal scrobe: lower, mesal area smooth, remainder of scrobe with weak coriaceous sculpture. Shape and size of anterior ocellus: small, very circular in shape. Vertex posterior area sculpture: with dense, setigerous punctulate sculpture. Occipital carina at dorsal area: present, not conspicuously present. Netrion: practically absent by presence of longitudinal carinae on pronotal lateral areas. Sculpture of mesepisternum; absent (smooth). Sculpture of pronotal lateral areas: with dense, fine, sinuate, transverse carinae. Length of fore wing stigmal vein: short. Angle of stigmal vein in relation to anterior margin of fore wing: at an angle of approximately 45° with respect to anterior margin of wings. Sculpture of T2: mainly smooth except lateral areas with few, sparse, weak carinae.

##### Diagnosis.

This species can be separated from all *Odontacolus* species with an obscured netrion by the well-defined and wide notauli with clearly defined large crenulae throughout, the broad rugulose sculpture on frons and mesoscutum, and the yellow body color.

##### Etymology.

This species is named after the Australian entomologist and invertebrate photographer Paul Zborowski. The epithet is a noun in the genitive case.

##### Link to distribution map.

100

##### Material examined.

Holotype female: **AUSTRALIA**: QLD, Heathlands, 11°45'S, 142°35'E, 25.VII–18.VIII.1992, Malaise trap, P. Zborowski & J. Cardale, OSUC 237939 (deposited in ANIC). *Paratypes*: **AUSTRALIA**: 57 females, 3 males, OSUC 237938, 237940–237943, 237945–237948, 237950–237956, 237958–237973, 237976–237998, 238424 (ANIC); OSUC 237949, 237957, 237975 (OSUC); OSUC 237944 (WINC).

##### Comments.

The holotype is in perfect condition as are the paratypes. In some specimens (e.g. OSUC 237956) the area between the propodeal anterior spines has some dark honey yellow color instead of the typical yellow.

#### Odontacolus zimi

Valerio & Austin sp. n.

urn:lsid:zoobank.org:act:1347681A-D163-4666-BB0C-4F223B654C6D

urn:lsid:biosci.ohio-state.edu:osuc_concepts:302168

http://species-id.net/wiki/Odontacolus zimi

Figures 287–292 ; Morphbank ^101^

##### Description.

*Female*. Body length: 1.49 mm (n=1). Antenna color: completely yellow. Body color: head and mesosoma dark brown, head darker than mesosoma, metasoma light honey yellow. Coxae color: yellow. Leg color (excluding coxae): yellow. Fore wing color: slightly infuscate throughout.

*Head*. Size of compound eye: approximately 1/2× height of head. Head shape in lateral view: lower head moderately short and strongly narrowed towards mouth, head appearing short and broad. Sculpture of antennal scrobe: smooth throughout. Surface of torular triangle: slightly bulging. Development of central keel on frons: present, elongate (equal to or greater than 1/3× height of frons), but not reaching anterior ocellus. Sculpture on upper frons below anterior ocellus: coriaceous throughout. Sculpture of malar space: with fan-like striae, striae not extending into antennal scrobe. Furrow at lateral portion of antennal scrobe: absent. Mesal surface of vertex: flat to weakly convex. Size of lateral ocelli: minute. Distance between lateral ocellus and occipital carina: 0.5–1.2× maximum ocellar diameter. Lagrimal: absent or minute. Length of OOL: less than or equal to 1/3× width of ocellus. Sculpture of vertex: coriaceous. Sculpture of occipital carina: weakly crenulate throughout. Distance from occipital carina to orbital carina: contiguous or nearly so, subequal to width of occipital carina. Shape of occipital carina: simply arcuate medially. Sculpture of occiput: with weakly rugulo aciculate sculpture. Sculpture of gena: coriaceous dorsally, otherwise weakly rugulose.

*Mesosoma*. Dorsal mesosoma in lateral view: convex. Sculpture of pronotal lateral area: dorsal 2/3 punctate, otherwise smooth. Netrion: present, smooth, linear. Notaulus: present, simple. Length of `notaulus: approximately less than or equal to 1/3 of length of mesoscutum. Width of notaulus: narrow (notaulus width less than or equal to half the width of tegula). Sculpture of mesoscutum: anterior half coriaceous, otherwise densely granulose. Sculpture of mesoscutellum: granulose. Mesoscutellar profile: mainly flat, anterior and posterior edge at same height or nearly so. Mesoscutellar shape: flat, not depressed. Lateral propodeal area: sparsely transversely carinate. Shape of propodeal anterior spine: short, broad, apex subtriangular. Sculpture of propodeum between anterior spines: smooth or largely smooth. Sculpture of ventral half of mesepisternum: longitudinally costate. Sculpture of upper 1/4 of mesopleuron: densely longitudinally costate across entire width. Metapleural sculpture: smooth.

*Wings*. Stigmal vein: present, elongate, narrow. Campaniform sensilla at distal area of stigmal vein: present.

*Metasoma*. Shape of T1 horn: narrow, short. Sculpture of upper portion of T1 horn: longitudinally carinate. Sculpture of posterior portion of T1 horn: longitudinally carinate. Lateral carinae on T2: present, poorly defined. Sculpture of T2: finely costate, nearly smooth laterally. Sculpture of T3: smooth mesally, otherwise weakly coriaceous. Sculpture of S3–S6: S3 weakly coriaceous, S4–S6 mainly smooth with few, sparse, setigerous punctulae sculpture. S2 anterior carina: absent.

*Male*. Unknown.

##### Diagnosis.

This species can be separated from all the species which have the occipital carina almost touching the orbital carina by the absence of the S2 anterior carina, the posterior medial area of the mesoscutellum not depressed, and the complete absence of the lagrimal. The closest species is *Odontacolus jacksonae*, but the body of this species is completely dark brown in contrast with the honey yellow metasoma and dark brown head and mesosoma of *Odontacolus zimi*.

##### Etymology.

This species is named after the anime character ‘Invader Zim’, in reference to the invasion of the spider egg sacs that occurs when *Odontacolus* oviposit. The epithet is a noun in the genitive case.

##### Link to distribution map.

102

##### Material examined.

Holotype female: **MADAGASCAR**: Prov. Antsiranana, Sakalava Beach, dwarf littoral for., 10m, 15.ii–6.iii.2001 Malaise, across sandy trail, R. Harin’Hala coll. MA-01-048-03, 12°15'46"S, 49°23'51"E; CASENT 2136841 (CASC).

**Comments**. The holotype is in perfect condition.

## Plates

**Figure 5–10.**
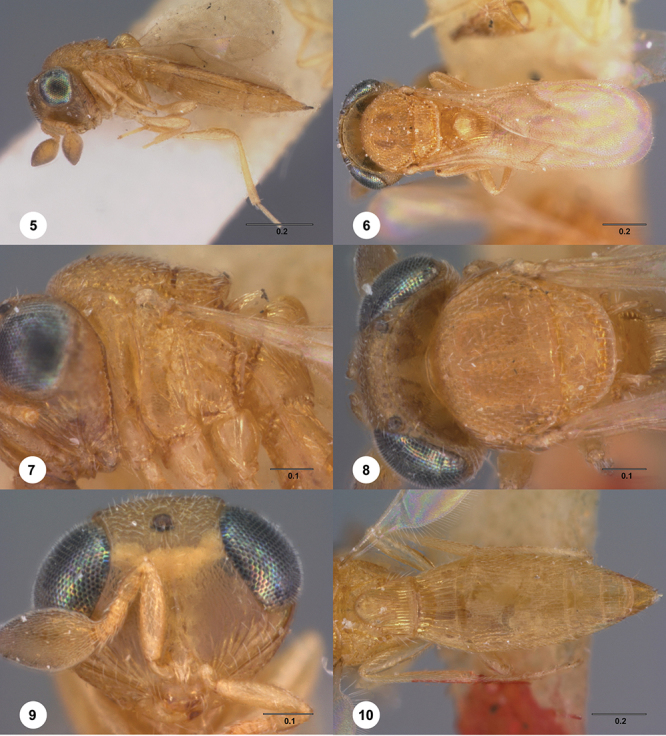
135 *Idris longispinosus*, female paratype (USNM Type No. 20634 PLT) **5** Lateral habitus **6** Dorsal habitus **7** Mesosoma, lateral view **8** Mesosoma, dorsal view **9** Head, anterior view **10** Metasoma, dorsal view. Scale bars in mm.

**Figures 11–16.**
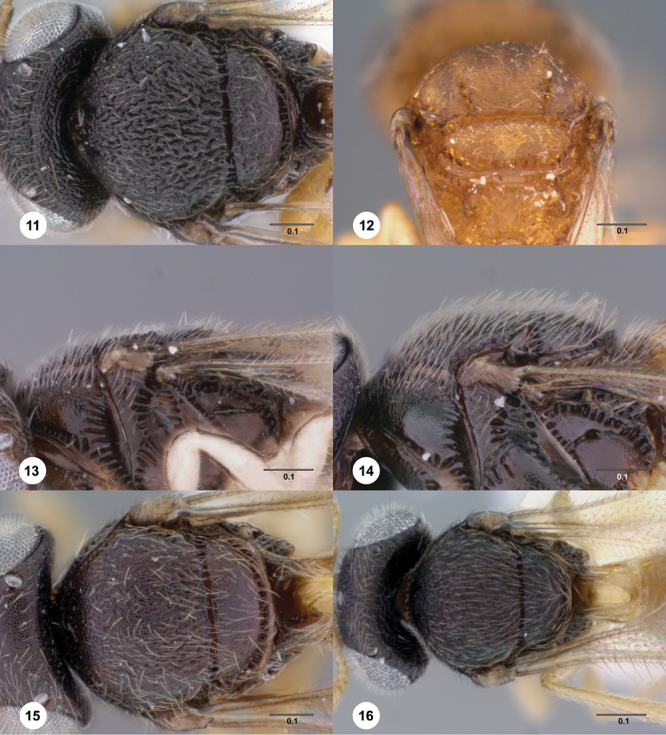
136 **11** *Odontacolus heratyi*, mesosoma, dorsal view, female paratype (FBA014379) **12** *Odontacolus gallowayi*, mesoscutellum, posterodorsal view, femaleholotype (OSUC 237999) **13** *Odontacolus schlingeri*, mesosoma, lateral view, female holotype (FBA104329) **14** *Odontacolus veroae*, mesosoma, lateral view, female paratype (FBA014430) **15** *Odontacolus veroae*, mesosoma, dorsal view, female paratype (FBA081512) **16** *Odontacolus irwini*, mesosoma, dorsal view, female paratype (FBA059072). Scale bars in mm.

**Figures 17–22.**
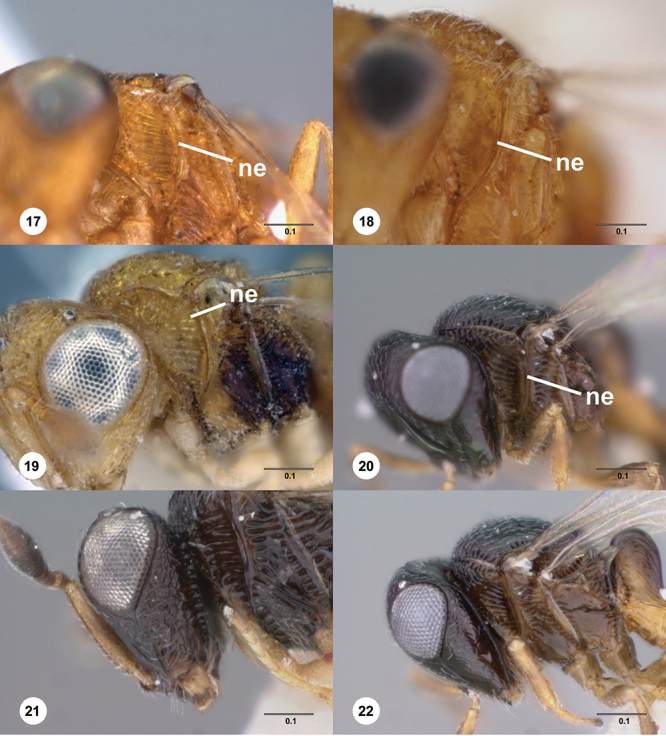
137 **17** *Odontacolus harveyi*, netrion, lateral view, female paratype (OSUC 237929) **18** *Odontacolus noyesi*, netrion, lateral view, female paratype (OSUC 237931) **19** *Odontacolus zborowskii*, netrion, lateral view, female paratype (OSUC 237946) **20** *Odontacolus pintoi*, netrion, lateral view, female paratype (OSUC 239099) **21** *Odontacolus berryae*, gena, lateral view, female paratype (OSUC 238580) **22** *Odontacolus pintoi*, gena, lateral view, female paratype (OSUC 239099). Scale bars in mm; ne: netrion. Morphbank.

**Figures 23–28.**
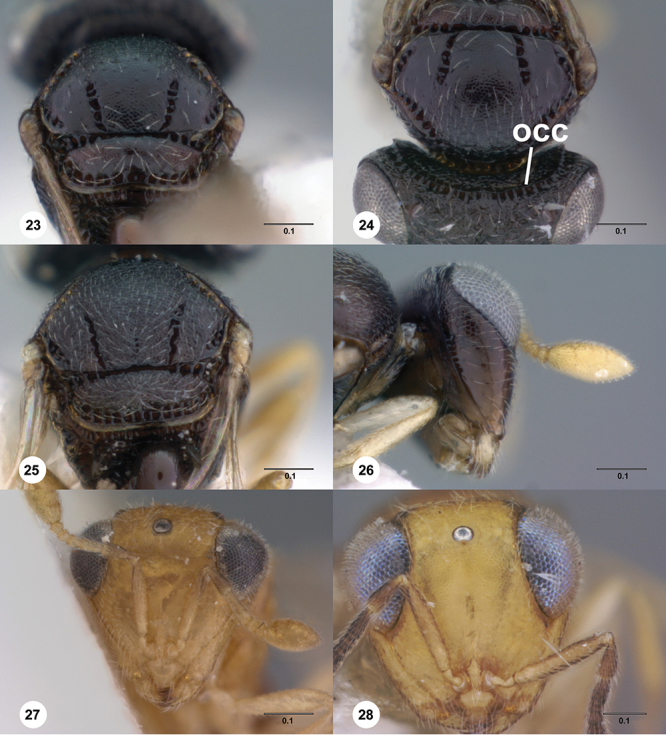
138 **23** *Odontacolus berryae*, mesoscutellum, posterodorsal view, female paratype (OSUC 238580) **24** *Odontacolus berryae*, occipital carina, dorsal view, female paratype (OSUC 238580) **25** *Odontacolus australiensis*, mesoscutellum, posterodorsal view, male paratype (OSUC 239120) **26** *Odontacolus madagascarensis*, gena, lateral view, female paratype (CASENT 2134814) **27** *Odontacolus noyesi*, head, anterior view, female paratype (OSUC 237931) **28** *Odontacolus wallacei*, head, anterior view, female paratype (OSUC 238004). Scale bars in mm.

**Figures 29–34.**
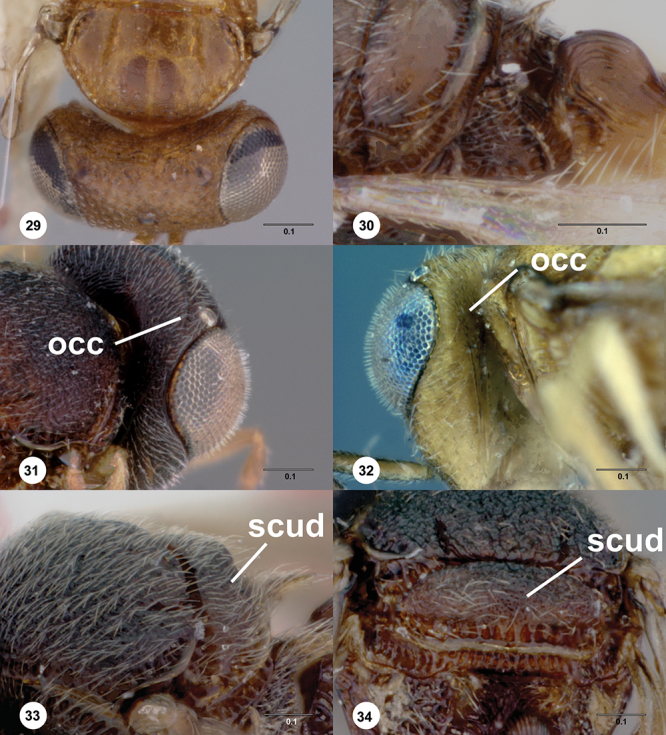
139 **29** *Odontacolus harveyi*, occipital carina, dorsal view, female paratype (OSUC 237919) **30** *Odontacolus madagascarensis*, propodeum, dorsolateral view, female paratype (CASENT 2134814) **31** *Odontacolus jacksonae*, occipital carina and orbital carina, posterodorsal view, female paratype (OSUC 238416) **32** *Odontacolus wallacei*, occipital carina and orbital carina, posterior view, female paratype (OSUC 148684) **33** *Odontacolus guineensis*, mesoscutellum, dorsolateral view **34** mesoscutellum, posterior view, female holotype (OSUC 237933). Scale bars in mm.

**Figures 35–40.**
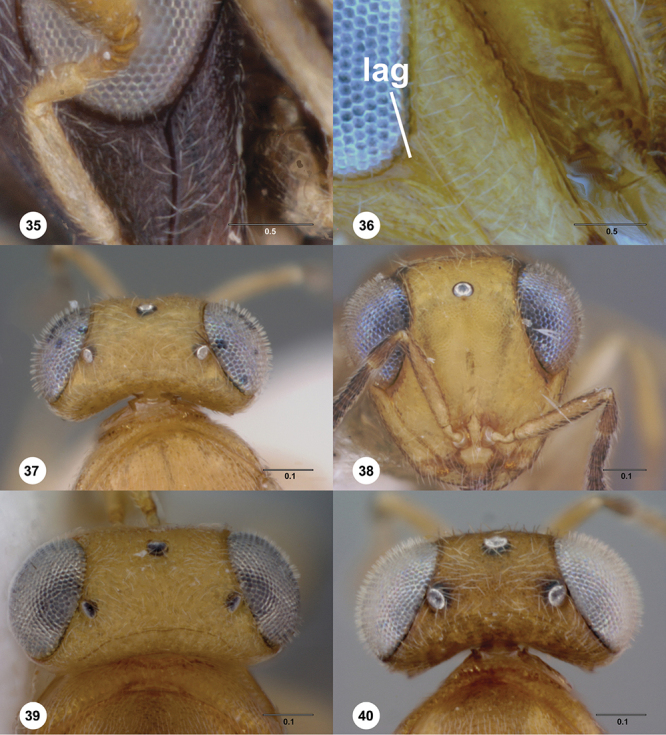
140 **35** *Odontacolus jacksonae*, lagrimal, lateral view, female paratype (OSUC 238416) **36** *Odontacolus gentingensis*, lagrimal, lateral view, female holotype (OSUC 237935) **37** *Odontacolus wallacei*, vertex, dorsal view, female holotype (OSUC 238001) **38** *Odontacolus wallacei*, head, anterior view, female paratype (OSUC 238004) **39** *Odontacolus cardaleae*, vertex, dorsal view, female holotype (OSUC 237936) **40** *Odontacolus heydoni*, vertex, dorsal view, female holotype (OSUC 239203). Scale bars in mm.

**Figures 41–46.**
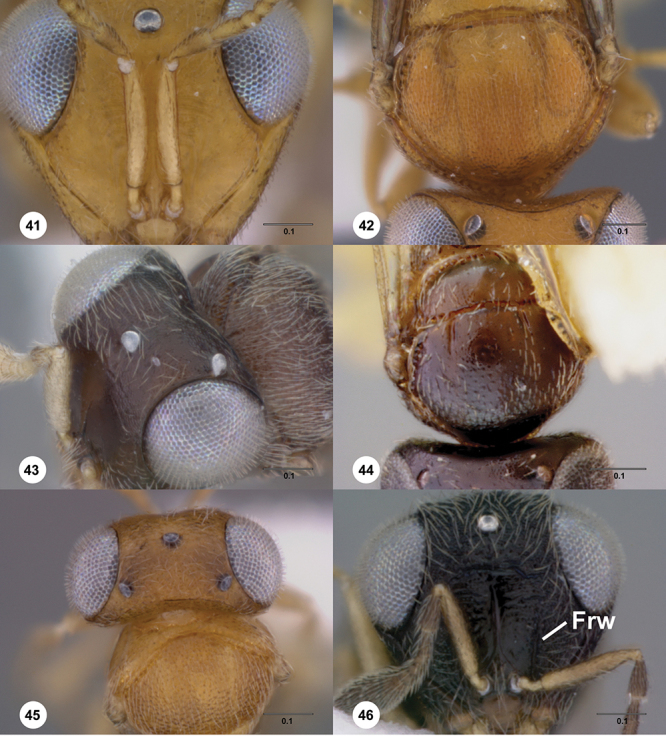
141 **41** *Odontacolus heydoni*, head, anterior view, female paratype (OSUC 268731) **42** *Odontacolus heydoni*, mesoscutum, dorsal view, female paratype (OSUC 268731) **43** *Odontacolus dayi*, vertex, dorsolateral view, female holotype (OSUC 238418) **44** *Odontacolus longiceps*, mesoscutum, dorsal view, female holotype (BMNH TYPE HYM 9400) **45** *Odontacolus lamarcki*, head, dorsal view, female holotype (OSUC 339597) **46** *Odontacolus sharkeyi*, head, anterior view, female holotype (OSUC 321869). Scale bars in mm.

**Figures 47–52.**
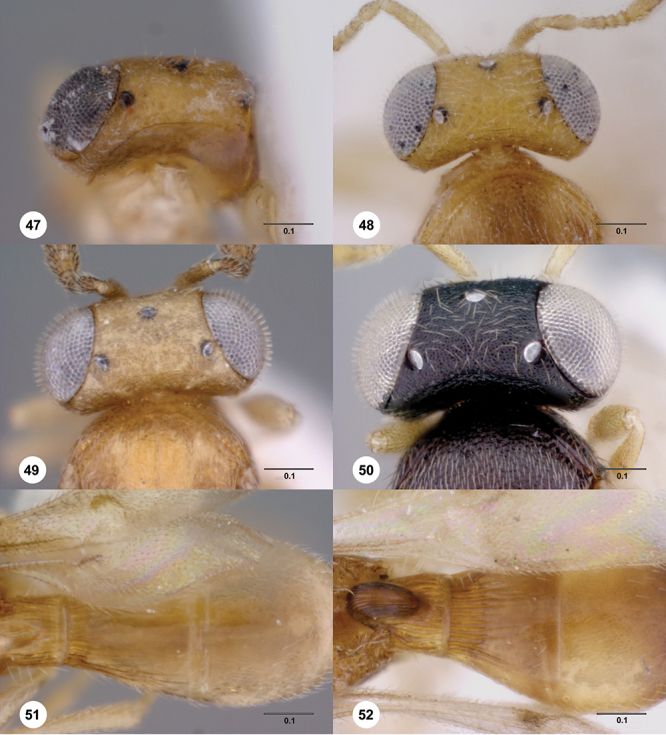
142 **47** *Odontacolus spinosus*, head, dorsal view, female holotype (SAMA DB32-001530) **48** *Odontacolus baeri*, head, dorsal view, female holotype (OSUC 238430) **49** *Odontacolus anningae*, head, dorsal view, female holotype (OSUC 238445) **50** *Odontacolus africanus*, head, dorsal view, female paratype (CASENT 2042645) **51** *Odontacolus anningae*, T1–T3 dorsal view, female holotype (OSUC 238445) **52** *Odontacolus baeri*, T1–T3 dorsal view, female holotype (OSUC 238430). Scale bars in mm.

**Figures 53–58.**
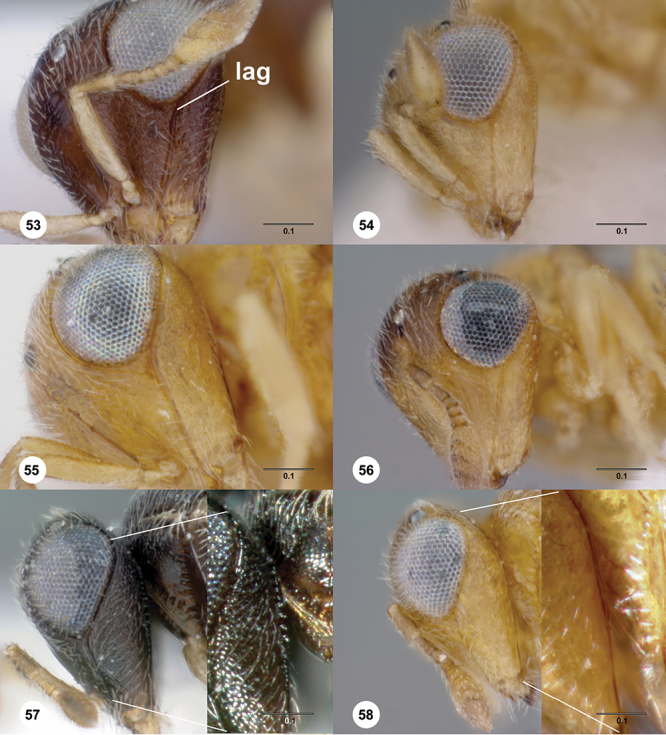
143 **53** *Odontacolus aldrovandii*, face, anterolateral view, female holotype (OSUC 239210) **54** *Odontacolus markadicus*, face, anterolateral view, female (OSUC 238767) **55** *Odontacolus mot*, head, lateral view, female holotype (OSUC 239205) **56** *Odontacolus markadicus*, head, lateral view, female (OSUC 239190) **57** *Odontacolus bosei*, head, lateral view, female holotype (OSUC 238799) **58** *Odontacolus markadicus*, head, lateral view, female (OSUC 239192). Scale bars in mm.

**Figures 59–64.**
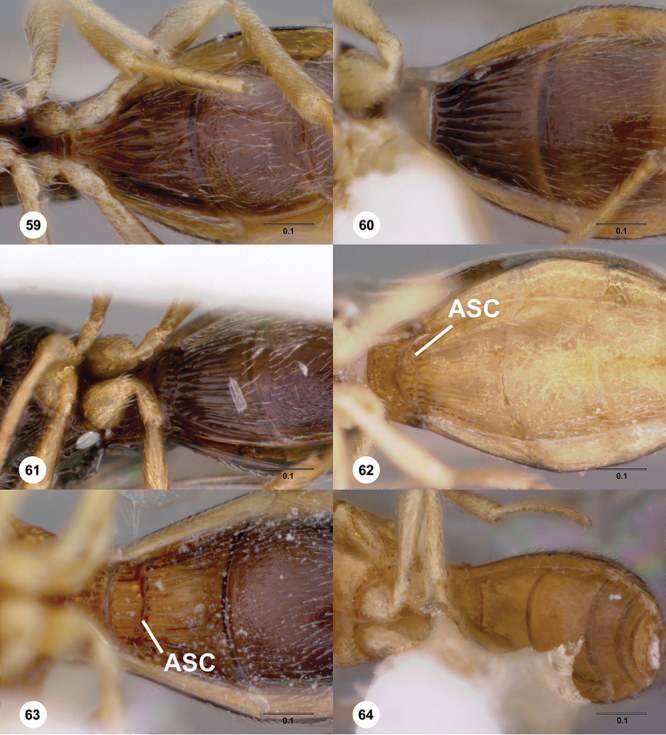
144 **59** *Odontacolus kiau*, S2, ventral view, femaleholotype (OSUC 239159) **60** *Odontacolus veroae*, S2, ventral view, female paratype (FBA030106) **61** *Odontacolus australiensis*, S2, ventral view, female paratype (OSUC 239118) **62** *Odontacolus zborowskii*, S2, ventral view, female paratype (OSUC 237945) **63** *Odontacolus darwini*, S2, ventral view, female paratype (UCRENT 135138) **64** *Odontacolus lamarcki*, S2, ventral view, femaleholotype (OSUC 339597). Scale bars in mm.

**Figures 65–70.**
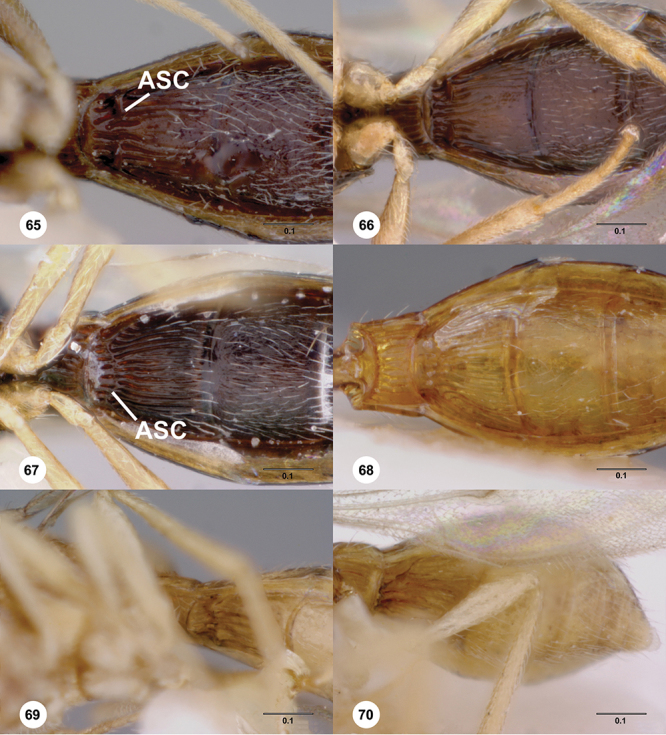
145 **65** *Odontacolus jacksonae*, S2, ventral view, female paratype (OSUC 238416) **66** *Odontacolus africanus*, S2, ventral view, female paratype (OSUC 238722) **67** *Odontacolus berryae*, S2, ventral view, female paratype (OSUC 238560) **68** *Odontacolus gallowayi*, S2, ventral view, female holotype (OSUC 237999) **69** *Odontacolus anningae*, S2, ventral view, female holotype (OSUC 238445) **70** *Odontacolus baeri*, S2, ventral view, female holotype (OSUC 238430).Scale bars in mm.

**Figures 71–76.**
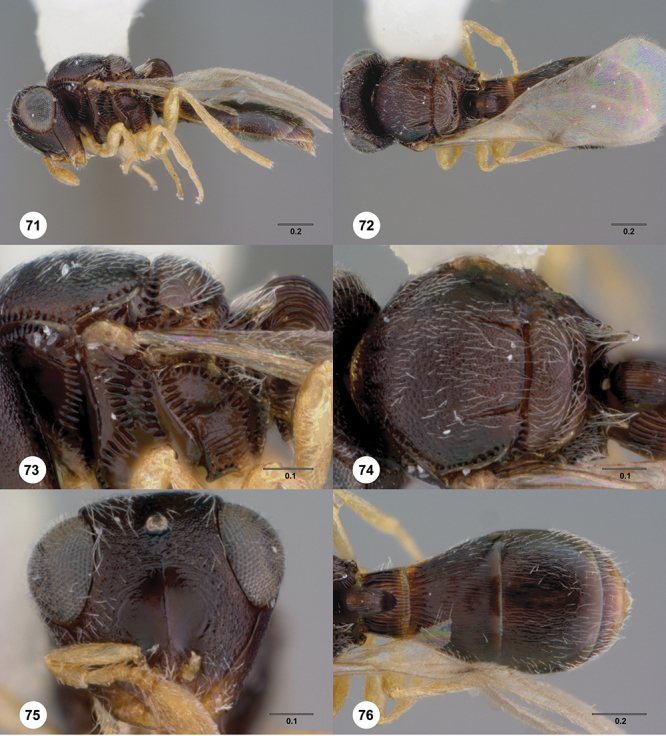
146 *Odontacolus africanus*, female holotype (OSUC 238419). **71** Lateral habitus **72** Dorsal habitus **73** Mesosoma, lateral view **74** Mesosoma, dorsal view **75** Head, anterior view **76** Metasoma, dorsal view. Scale bars in mm.

**Figures 77–82.**
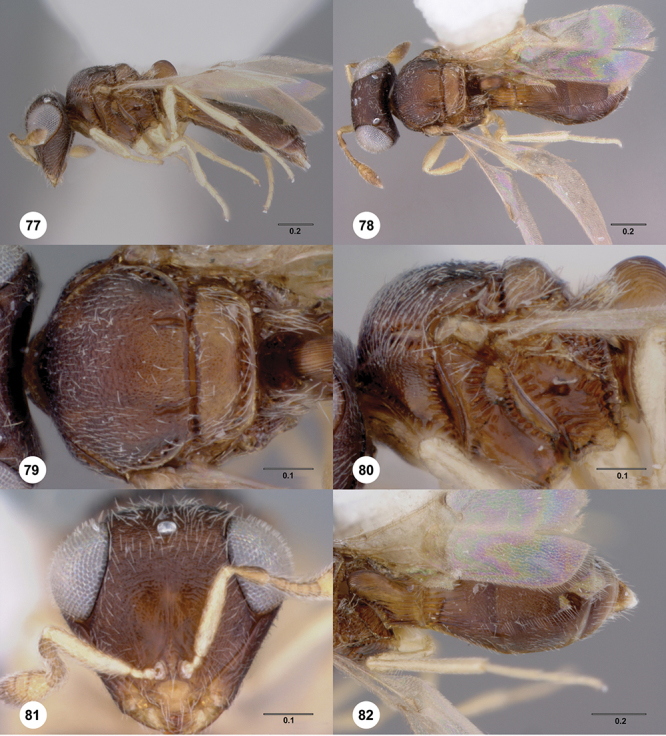
147 *Odontacolus aldrovandii*, female holotype (OSUC 239210) **77** Lateral habitus **78** Dorsal habitus **79** Mesosoma, lateral view **80** Mesosoma, dorsal view **81** Head, anterior view **82** Metasoma, dorsal view. Scale bars in mm.

**Figures 83–88.**
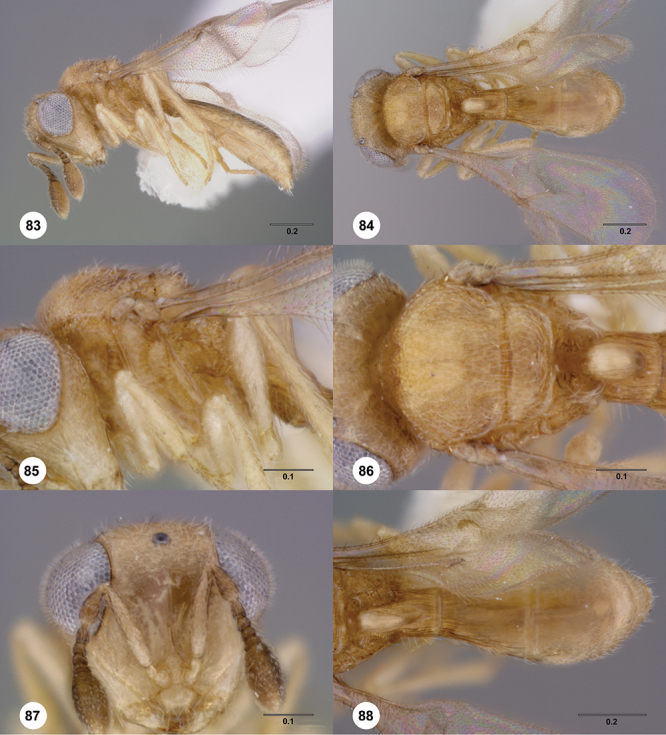
148 *Odontacolus anningae*, female holotype (OSUC 238445) **82** Lateral habitus **83** Dorsal habitus **84** Mesosoma, lateral view **85** Mesosoma, dorsal view **86** Head, anterior view **87** Metasoma, dorsal view. Scale bars in mm.

**Figures 89–94.**
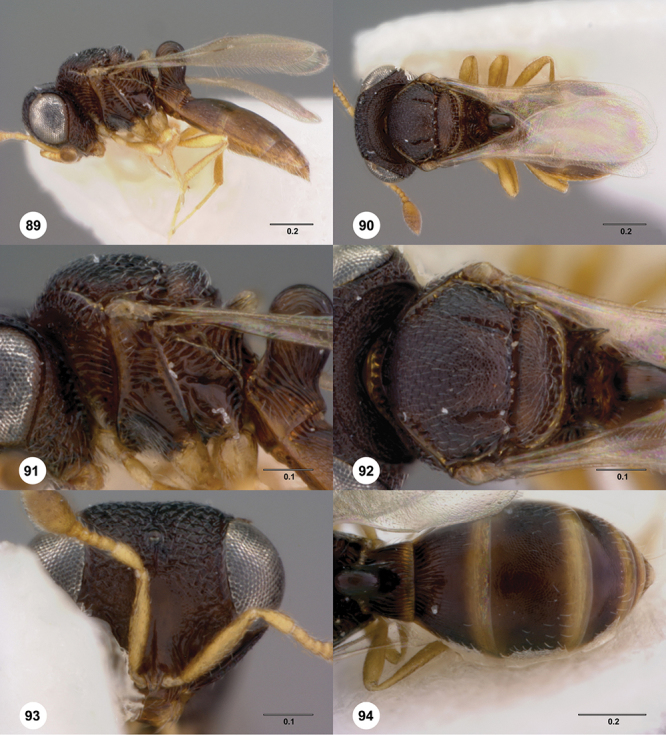
149 *Odontacolus australiensis*, female holotype (OSUC 239116) **88** Lateral habitus **90** Dorsal habitus **91** Mesosoma, lateral view **92** Mesosoma, dorsal view **93** Head, anterior view **94** Metasoma, dorsal view. Scale bars in mm.

**Figures 95–100.**
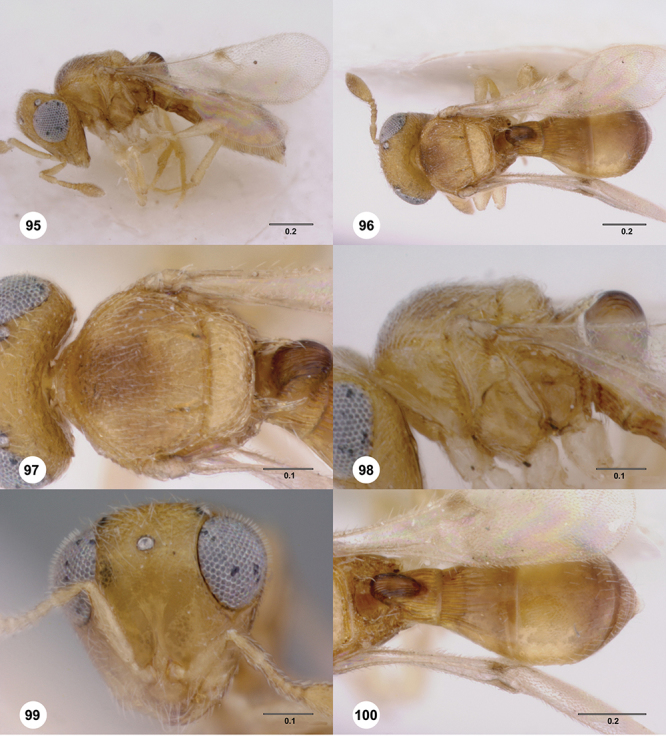
150 *Odontacolus baeri*, female holotype (OSUC 238430) **95** Lateral habitus **96** Dorsal habitus **97** Mesosoma, lateral view **98** Mesosoma, dorsal view **99** Head, anterior view **100** Metasoma, dorsal view. Scale bars in mm.

**Figures 101–106.**
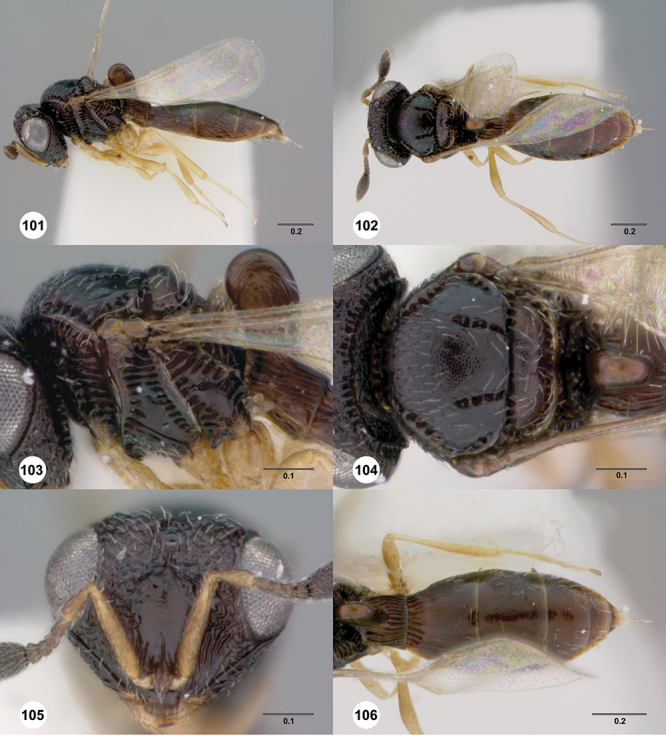
151 *Odontacolus berryae*, female paratype(OSUC 238580) **101** Lateral habitus **102** Dorsal habitus **103** Mesosoma, lateral view **104** Mesosoma, dorsal view **105** Head, anterior view **106** Metasoma, dorsal view. Scale bars in mm.

**Figures 107–112.**
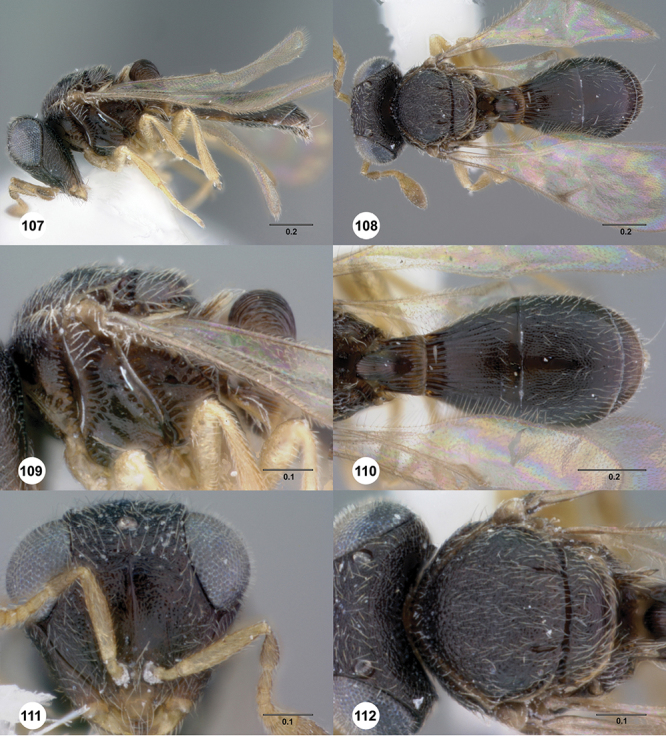
152 *Odontacolus bosei*, female paratype (OSUC 238799) **107** Lateral habitus **108** Dorsal habitus **109** Mesosoma, lateral view **110** Metasoma, dorsal view **111** Head, anterior view **112** Mesosoma, dorsal view. Scale bars in mm.

**Figures 113–118.**
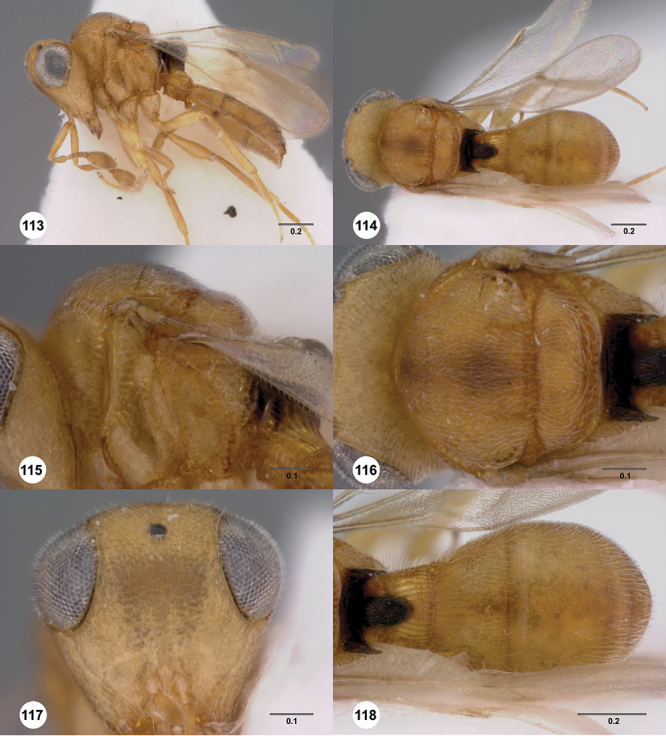
153 *Odontacolus cardaleae*, female holotype (OSUC 237936) **113** Lateral habitus **114** Dorsal habitus **115** Mesosoma, lateral view **116** Mesosoma, dorsal view **117** Head, anterior view **118** Metasoma, dorsal view. Scale bars in mm.

**Figures 119–124.**
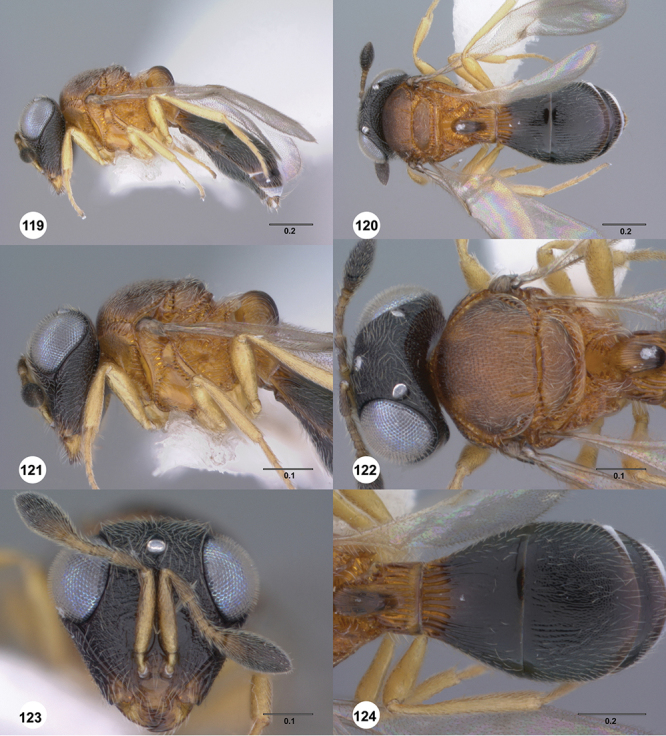
154 *Odontacolus darwini*, female holotype (OSUC 233092) **119** Lateral habitus **120** Dorsal habitus **121** Mesosoma, lateral view **122** Mesosoma, dorsal view **123** Head, anterior view **124** Metasoma, dorsal view. Scale bars in mm.

**Figures 125–130.**
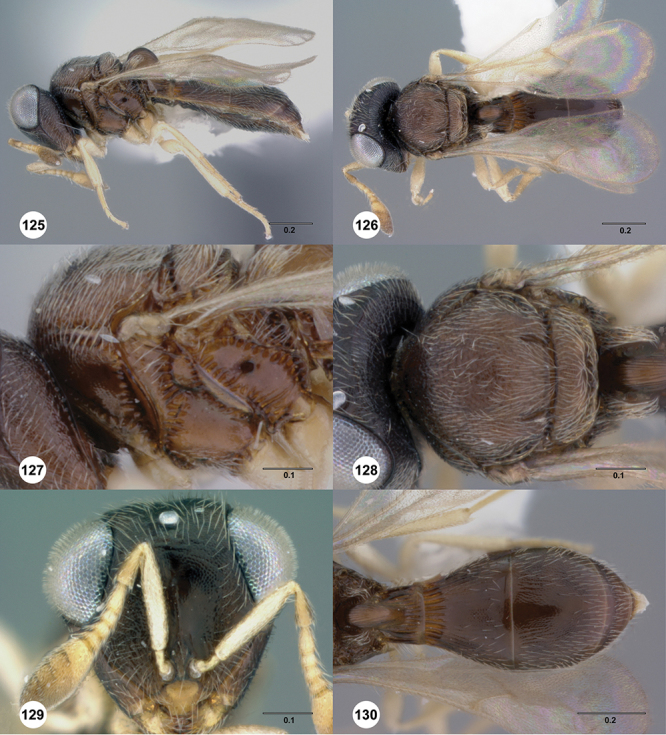
155 *Odontacolus dayi*, female holotype (OSUC 238418) **125** Lateral habitus **126** Dorsal habitus **127** Mesosoma, lateral view **128** Mesosoma, dorsal view **129** Head, anterior view **130** Metasoma, dorsal view. Scale bars in mm.

**Figures 131–136.**
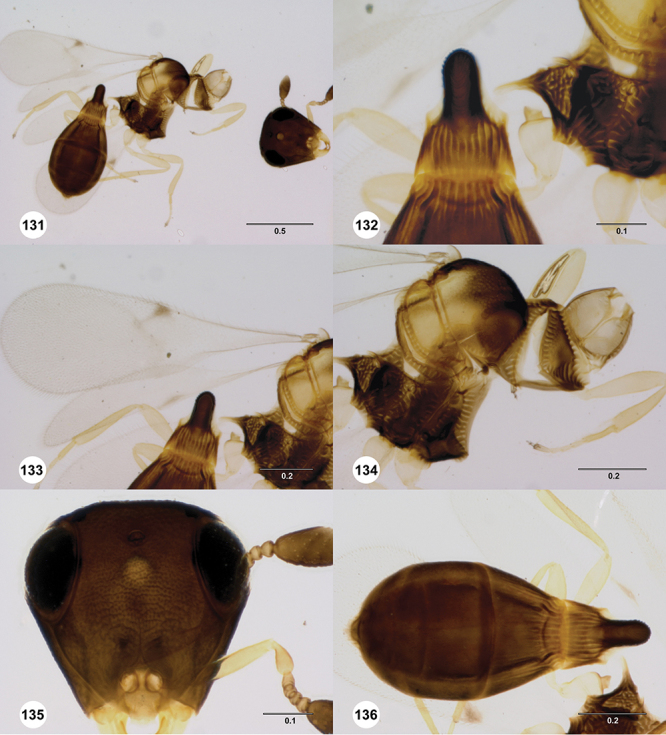
156 *Odontacolus doddi*, female holotype (QMBA HY1631) **131** slide, dorsal view **132** T1 horn, posterior view **133** wings, lateral view **134** Mesosoma, dorsal view **135** Head, anterior view **136** Metasoma, dorsal view. Scale bars in mm.

**Figures 137–142.**
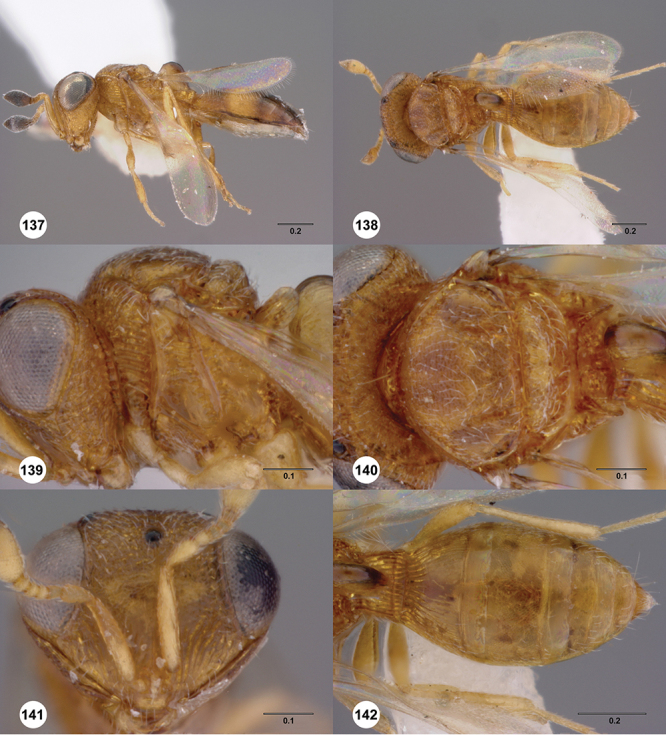
157 *Odontacolus gallowayi*, female holotype (OSUC 238000) **137** Lateral habitus **138** Dorsal habitus **139** Mesosoma, lateral view **140** Mesosoma, dorsal view **141** Head, anterior view **142** Metasoma, dorsal view. Scale bars in mm.

**Figures 143–148.**
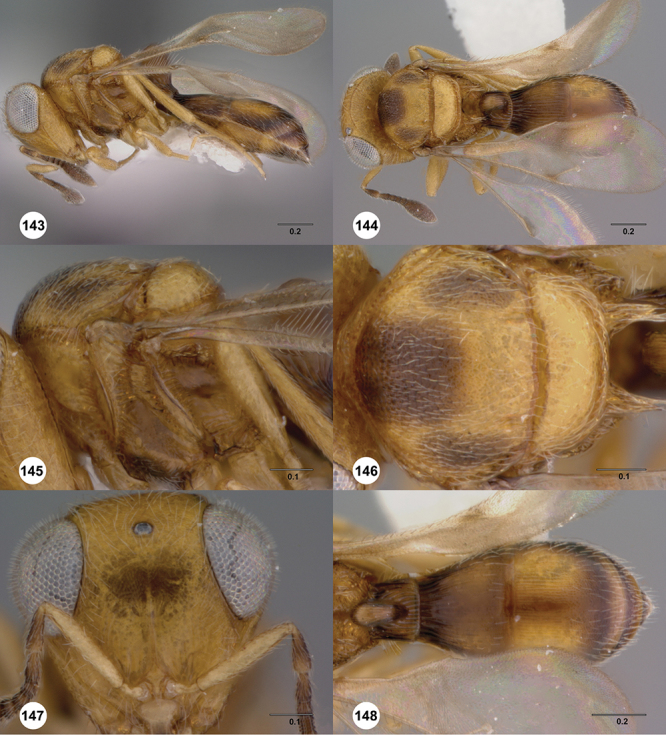
158 *Odontacolus gentingensis*, female holotype (OSUC 237935) **143** Lateral habitus **144** Dorsal habitus **145** Mesosoma, lateral view **146** Mesosoma, dorsal view **147** Head, anterior view **148** Metasoma, dorsal view. Scale bars in mm.

**Figures 149–154.**
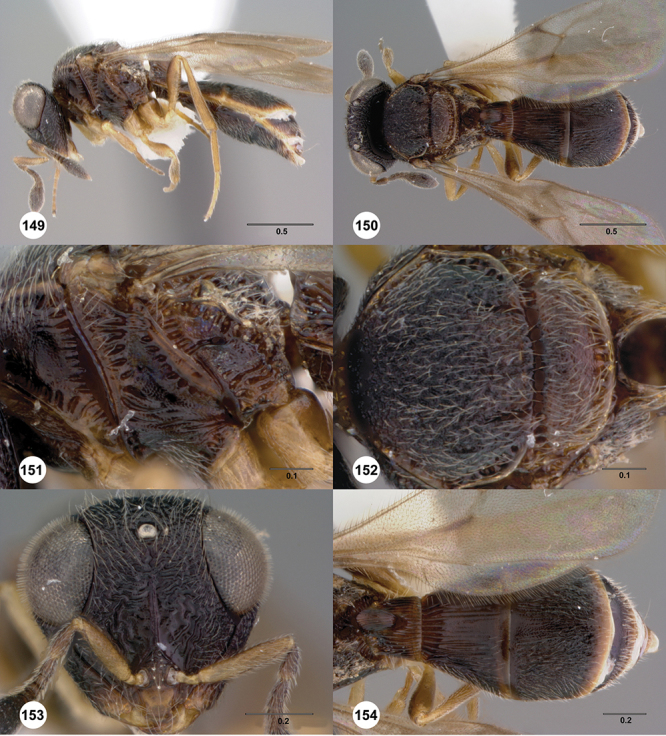
159 *Odontacolus guineensis*, female holotype (OSUC 237933) **149** Lateral habitus **150** Dorsal habitus **151** Mesosoma, lateral view **152** Mesosoma, dorsal view **153** Head, anterior view **154** Metasoma, dorsal view. Scale bars in mm.

**Figures 155–160.**
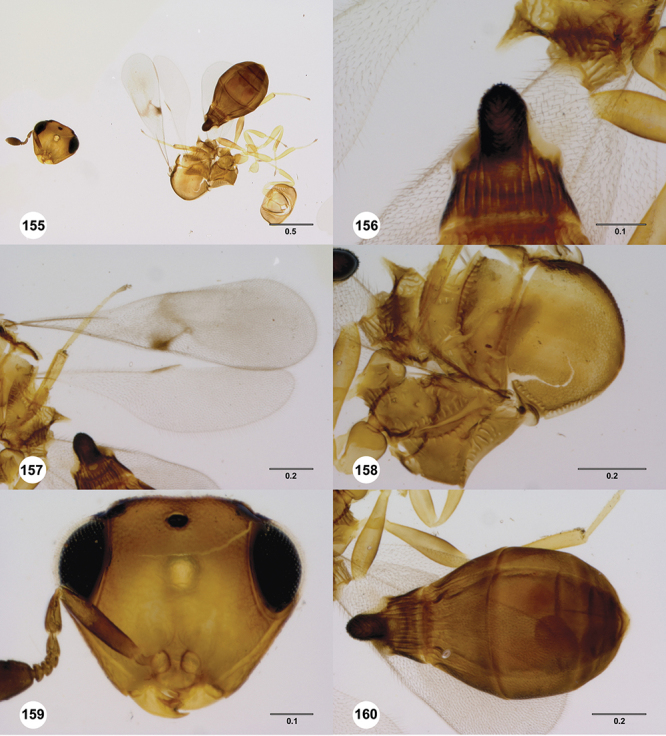
160 *Odontacolus hackeri*, female holotype (QMBA HY1630) **155** slide, dorsal view **156** T1 horn, posterior view **157** wings, lateral view **158** Mesosoma, dorsal view **159** Head, anterior view **160** Metasoma, dorsal view. Scale bars in mm.

**Figures 161–166.**
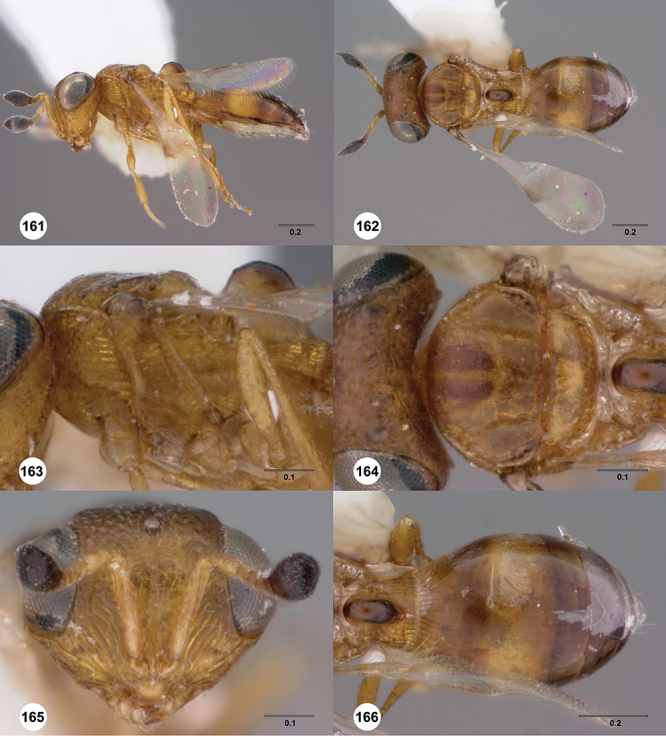
161 *Odontacolus harveyi*, female paratype (OSUC 237929) **161** Lateral habitus **162** Dorsal habitus **163** Mesosoma, lateral view **164** Mesosoma, dorsal view **165** Head, anterior view **166** Metasoma, dorsal view. Scale bars in mm.

**Figures 167–172.**
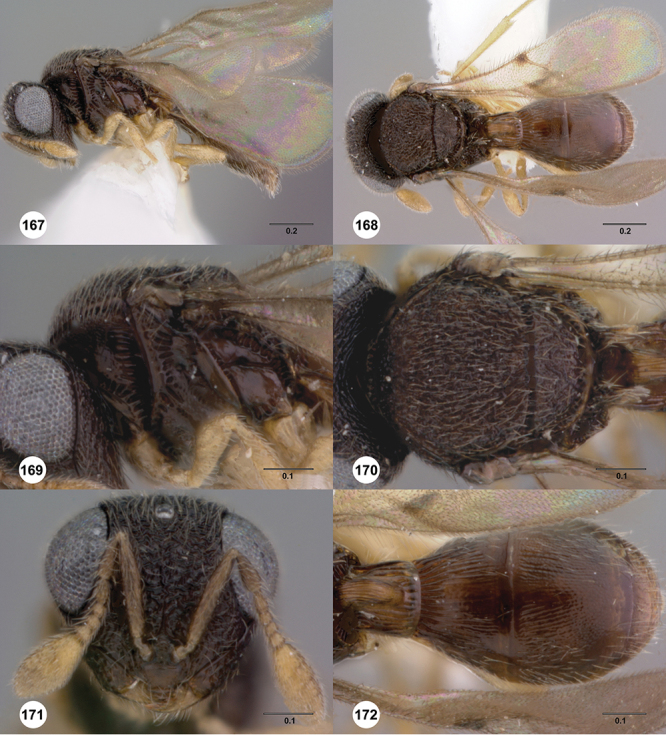
162 *Odontacolus heratyi*, female paratype (OSUC 237934) **167** Lateral habitus **168** Dorsal habitus **169** Mesosoma, lateral view **170** Mesosoma, dorsal view **171** Head, anterior view **172** Metasoma, dorsal view. Scale bars in mm.

**Figures 173–178.**
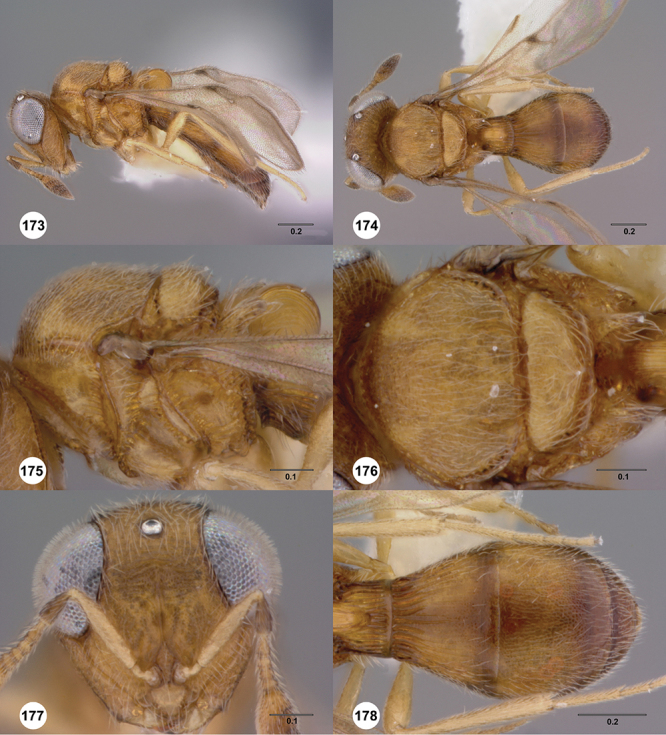
163 *Odontacolus heydoni*, female holotype (OSUC 239203) **173** Lateral habitus **174** Dorsal habitus **175** Mesosoma, lateral view **176** Mesosoma, dorsal view **177** Head, anterior view **178** Metasoma, dorsal view. Scale bars in mm.

**Figures 179–184.**
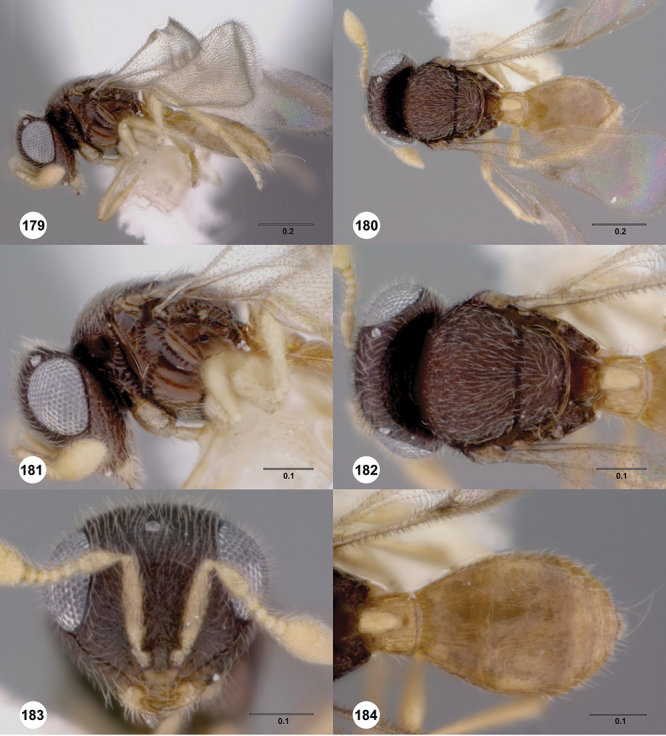
164 *Odontacolus irwini*, female paratype (FBA074688) **179** Lateral habitus **180** Dorsal habitus **181** Mesosoma, lateral view **182** Mesosoma, dorsal view **183** Head, anterior view **184** Metasoma, dorsal view. Scale bars in mm.

**Figures 185–190.**
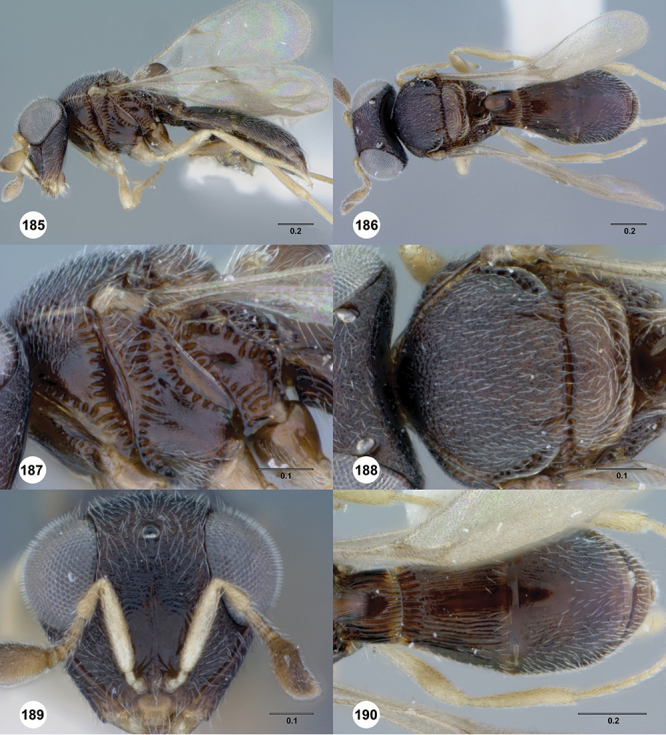
165 *Odontacolus jacksonae*, female holotype (OSUC 238415) **185** Lateral habitus **186** Dorsal habitus **187** Mesosoma, lateral view **188** Mesosoma, dorsal view **189** Head, anterior view **190** Metasoma, dorsal view. Scale bars in mm.

**Figures 191–196.**
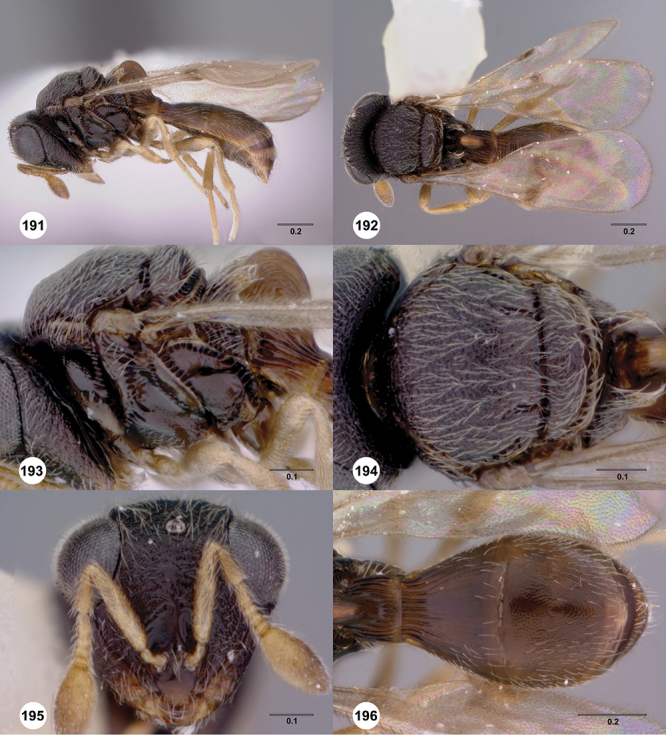
166 *Odontacolus kiau*, female holotype (OSUC 239159) **191** Lateral habitus **192** Dorsal habitus **193** Mesosoma, lateral view **194** Mesosoma, dorsal view **195** Head, anterior view **196** Metasoma, dorsal view. Scale bars in mm.

**Figures 197–202.**
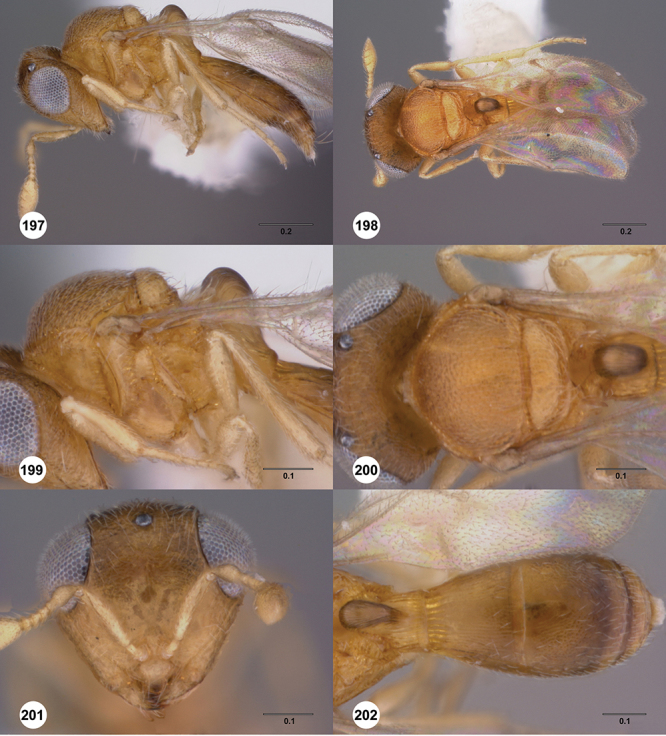
167 *Odontacolus lamarcki*, female holotype (OSUC 339597) **197** Lateral habitus **198** Dorsal habitus **199** Mesosoma, lateral view **200** Mesosoma, dorsal view **201** Head, anterior view **202** Metasoma, dorsal view. Scale bars in mm.

**Figures 203–208.**
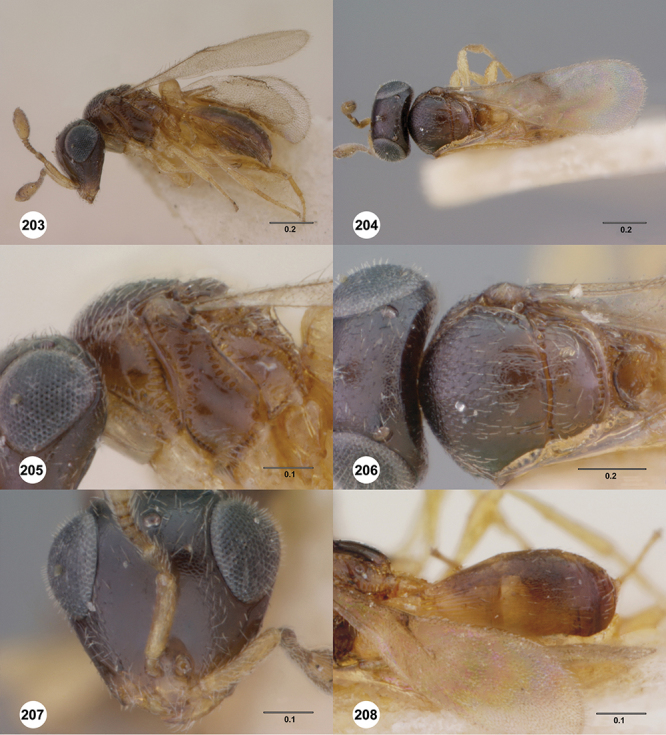
168 *Odontacolus longiceps*, female holotype (BMNH TYPEHYM 9400) **203** Lateral habitus **204** Dorsal habitus **205** Mesosoma, lateral view **206** Mesosoma, dorsal view **207** Head, anterior view **208** Metasoma, dorsal view. Scale bars in mm.

**Figures 209–214.**
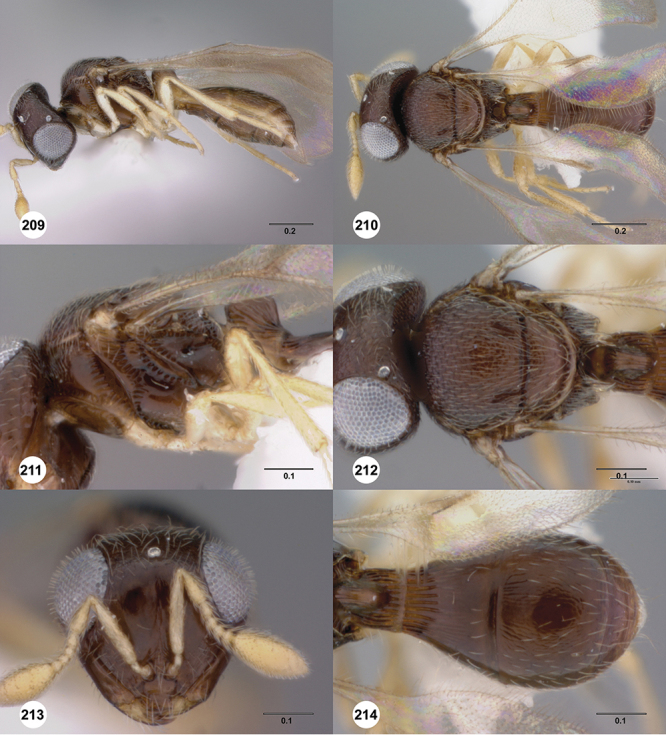
169 *Odontacolus madagascarensis*, female holotype (OSUC 238728) **209** Lateral habitus **210** Dorsal habitus **211** Mesosoma, lateral view **212** Mesosoma, dorsal view **213** Head, anterior view **214** Metasoma, dorsal view. Scale bars in mm.

**Figures 215–220.**
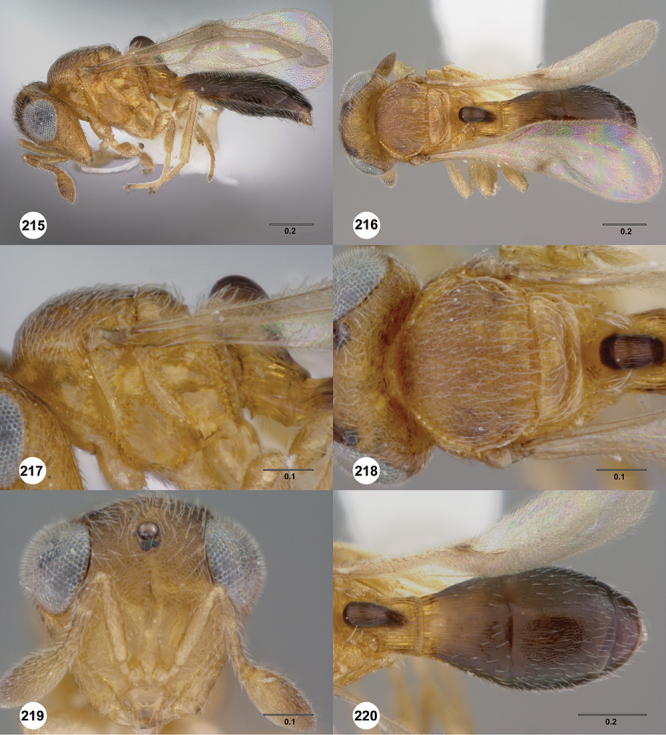
170 *Odontacolus markadicus*, female (OSUC 239191) **215** Lateral habitus **216** Dorsal habitus **217** Mesosoma, lateral view **218** Mesosoma, dorsal view **219** Head, anterior view **220** Metasoma, dorsal view. Scale bars in mm.

**Figures 221–226.**
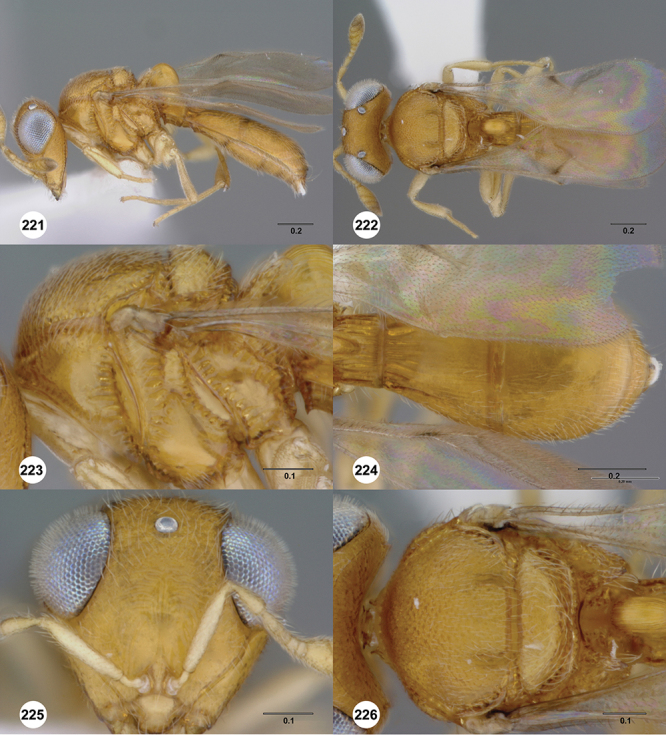
171 *Odontacolus mayri*, female holotype (OSUC 321876) **221** Lateral habitus **222** Dorsal habitus **223** Mesosoma, lateral view **224** Mesosoma, dorsal view **225** Head, anterior view **226** Metasoma, dorsal view. Scale bars in mm.

**Figures 227–232.**
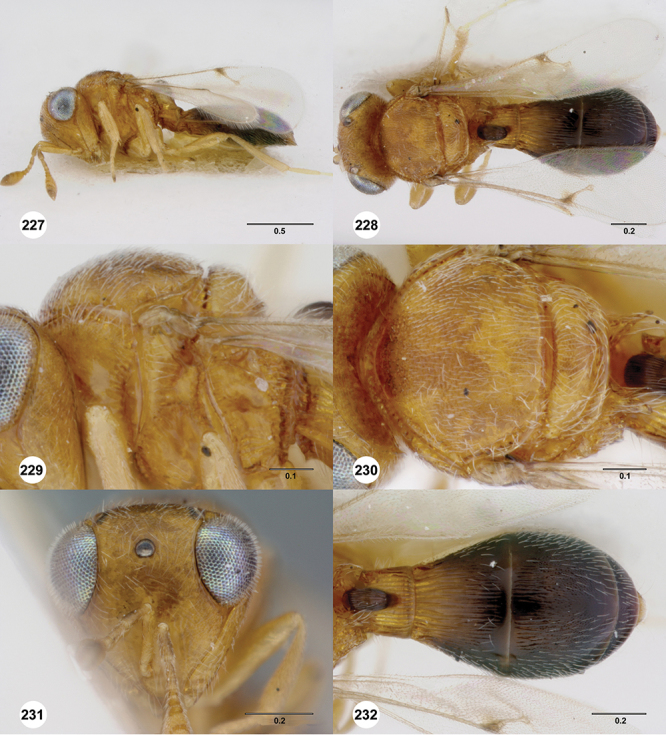
172 *Odontacolus mot*, female holotype (OSUC 239205) **227** Lateral habitus **228** Dorsal habitus **229** Mesosoma, lateral view **230** Mesosoma, dorsal view **231** Head, anterior view **232** Metasoma, dorsal view. Scale bars in mm.

**Figures 233–238.**
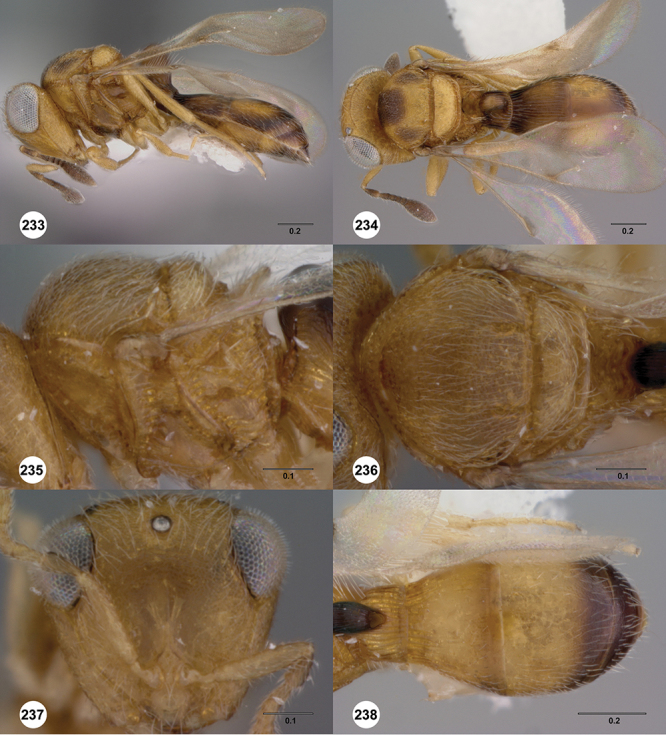
173 *Odontacolus noyesi*, female holotype (OSUC 237932) **233** Lateral habitus **234** Dorsal habitus **235** Mesosoma, lateral view **236** Mesosoma, dorsal view **237** Head, anterior view **238** Metasoma, dorsal view. Scale bars in mm.

**Figures 239–244.**
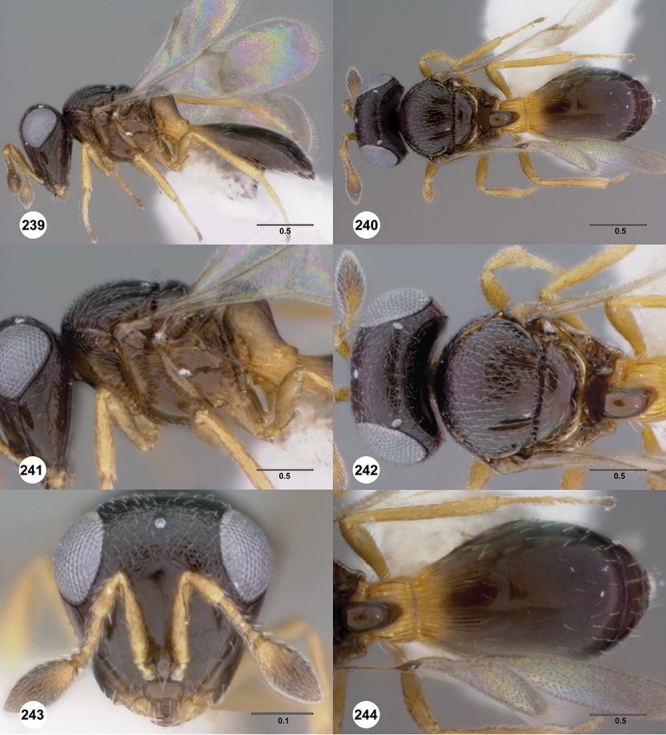
174 *Odontacolus pintoi*, female holotype (OSUC 239098) **239** Lateral habitus **240** Dorsal habitus **241** Mesosoma, lateral view **242** Mesosoma, dorsal view **243** Head, anterior view **244** Metasoma, dorsal view. Scale bars in mm.

**Figures 245–250.**
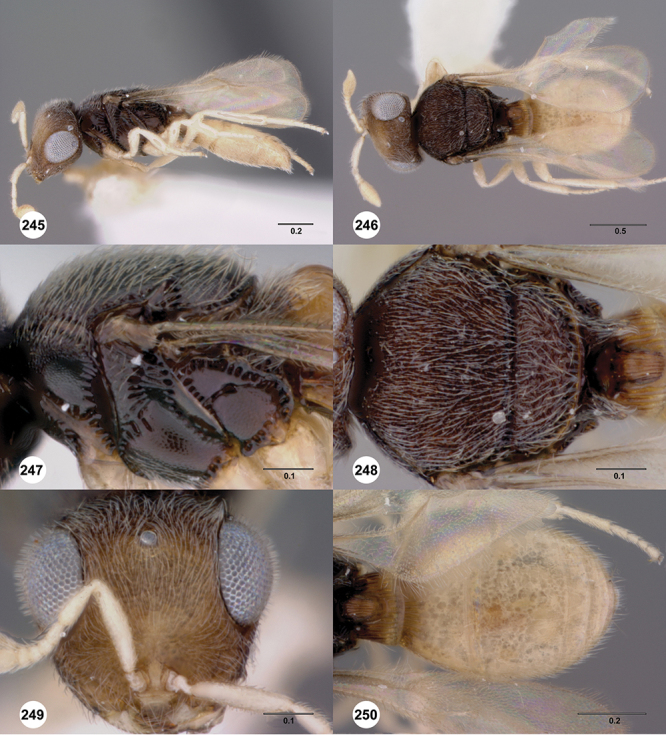
175 *Odontacolus schlingeri*, female holotype (FBA104329) **245** Lateral habitus **246** Dorsal habitus **247** Mesosoma, lateral view **248** Mesosoma, dorsal view **249** Head, anterior view **250** Metasoma, dorsal view. Scale bars in mm.

**Figures 251–256.**
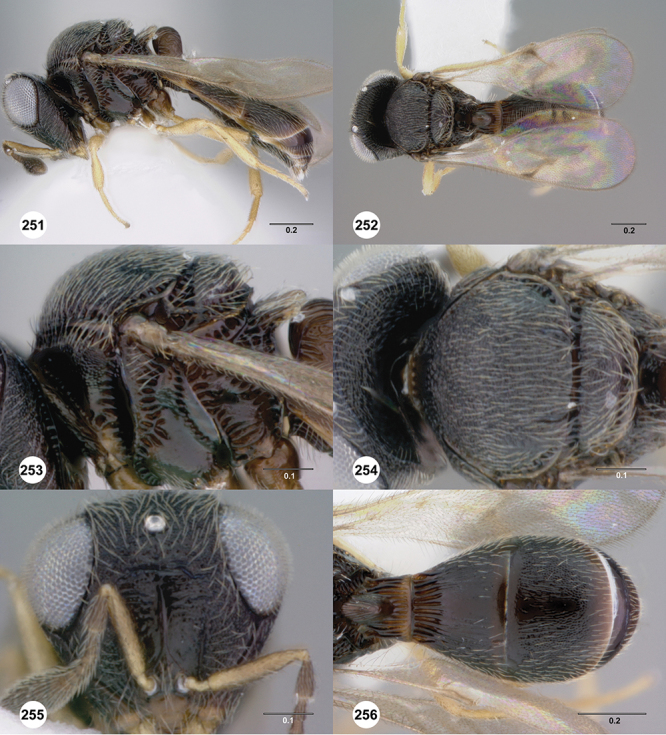
176 *Odontacolus sharkeyi*, female holotype (OSUC 321869) **251** Lateral habitus **252** Dorsal habitus **253** Mesosoma, lateral view **254** Mesosoma, dorsal view **255** Head, anterior view **256** Metasoma, dorsal view. Scale bars in mm.

**Figures 257–262.**
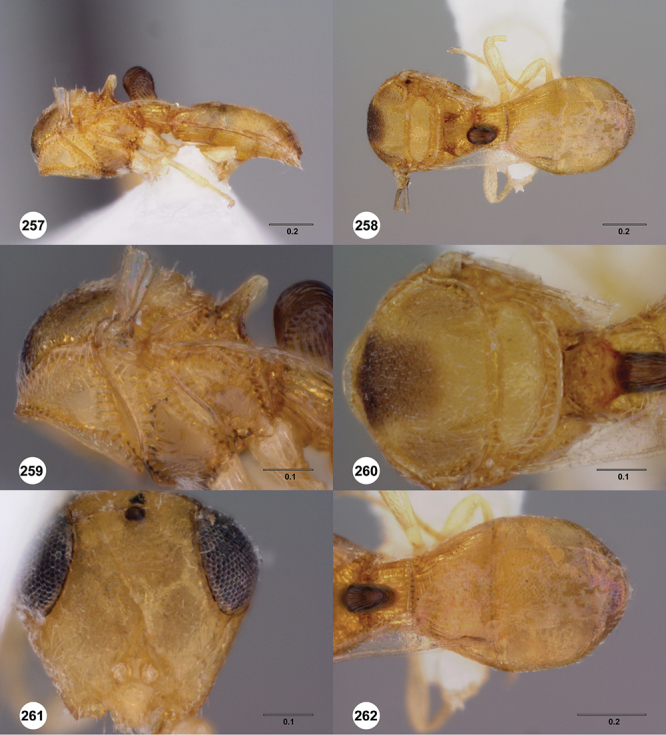
177 *Odontacolus spinosus*, female holotype (SAMA DB32-001530) **257** Lateral habitus **258** Dorsal habitus **259** Mesosoma, lateral view **260** Mesosoma, dorsal view **261** Head, anterior view **262** Metasoma, dorsal view. Scale bars in mm.

**Figures 263–268.**
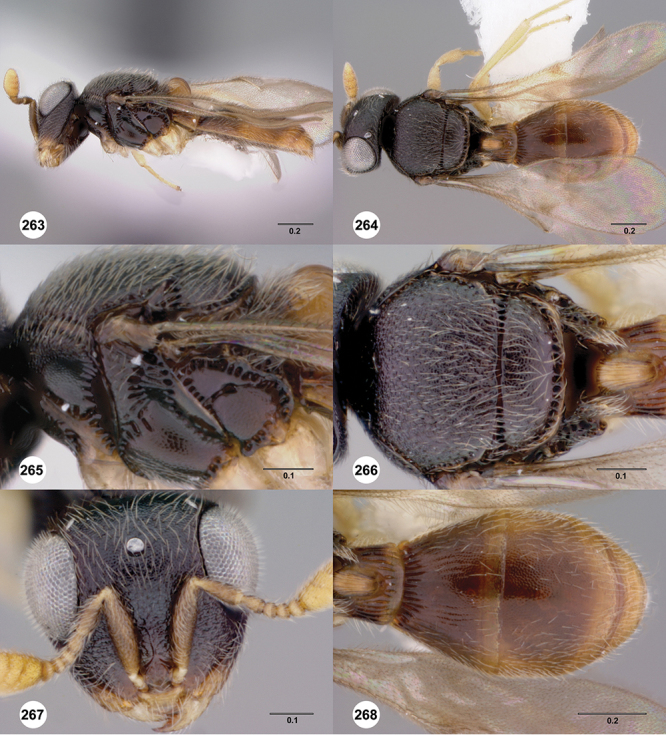
178 *Odontacolus veroae*, female paratype (FBA014430) **263** Lateral habitus **264** Dorsal habitus **265** Mesosoma, lateral view **266** Mesosoma, dorsal view **267** Head, anterior view **268** Metasoma, dorsal view. Scale bars in mm.

**Figures 269–274.**
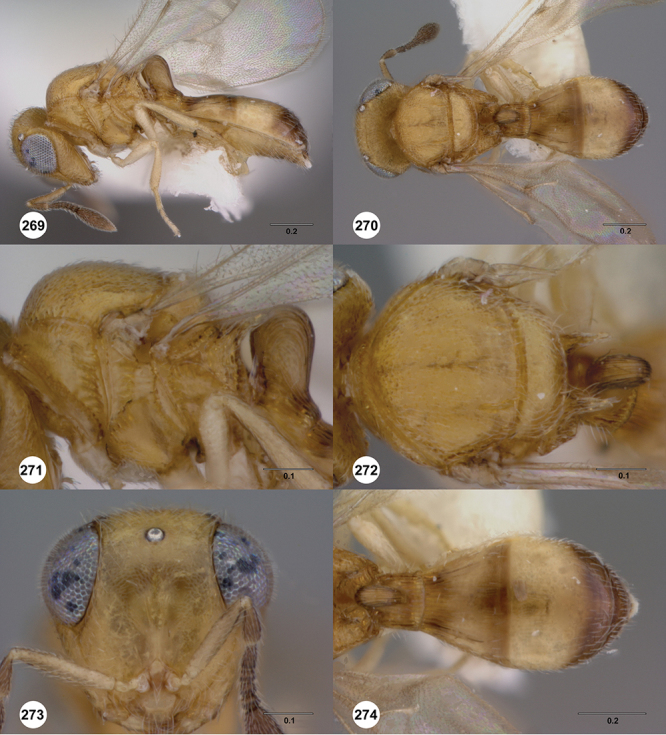
179 *Odontacolus wallacei*, female holotype (OSUC 238001) **269** Lateral habitus **270** Dorsal habitus **271** Mesosoma, lateral view **272** Mesosoma, dorsal view **273** Head, anterior view **274** Metasoma, dorsal view. Scale bars in mm.

**Figures 275–280.**
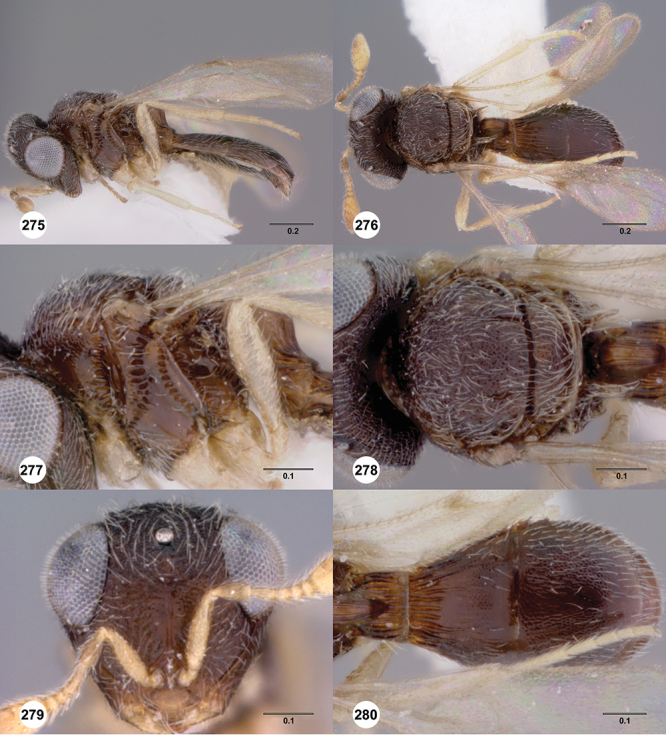
180 *Odontacolus whitfieldi*, female holotype (OSUC 239160) **275** Lateral habitus **276** Dorsal habitus **277** Mesosoma, lateral view **278** Mesosoma, dorsal view **279** Head, anterior view **280** Metasoma, dorsal view. Scale bars in mm.

**Figures 281–286.**
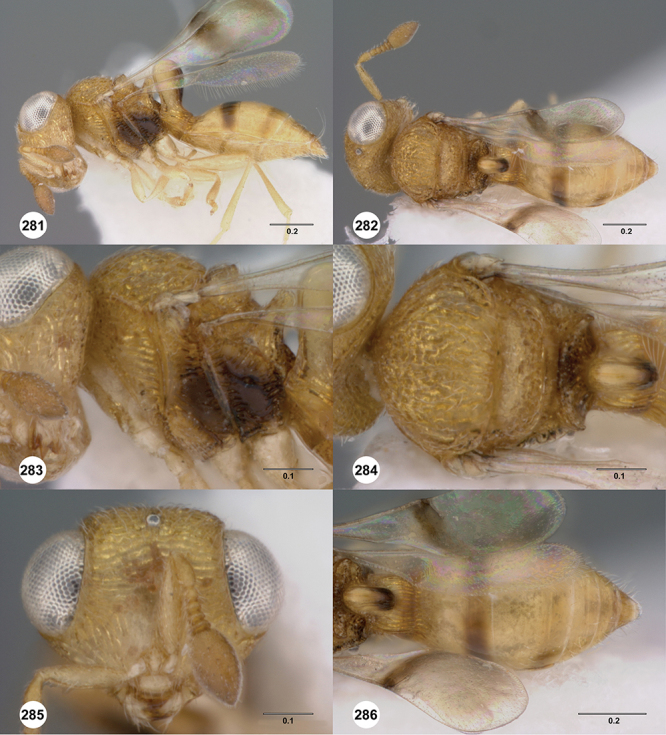
181 *Odontacolus zborowskii*, female holotype (OSUC 237939) **281** Lateral habitus **282** Dorsal habitus **283** Mesosoma, lateral view **284** Mesosoma, dorsal view **285** Head, anterior view **286** Metasoma, dorsal view. Scale bars in mm.

**Figures 287–292.**
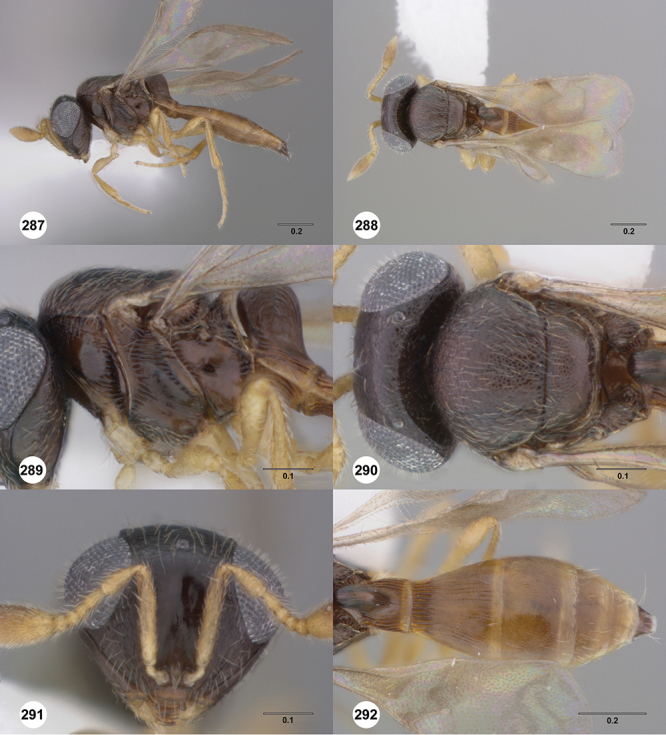
182 *Odontacolus zimi*, female holotype (CASENT 2136841) **287** Lateral habitus **288** Dorsal habitus **289** Mesosoma, lateral view **290** Mesosoma, dorsal view **291** Head, anterior view **292** Metasoma, dorsal view. Scale bars in mm.

**Figures 293–298.**
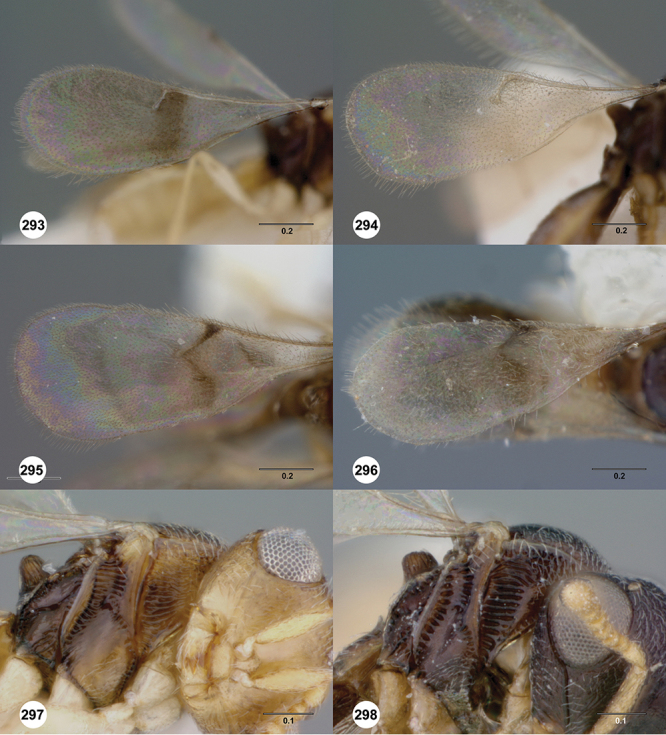
183 **293** *Odontacolus zborowskii*, fore wing, lateral view, male paratype (OSUC 237998) **294** *Odontacolus australiensis*, fore wing, lateral view, maleparatype (OSUC 239142) **295** *Odontacolus africanus*, fore wing, lateral view, male paratype (OSUC 238568) **296** *Odontacolus pintoi*, fore wing, lateral view, male paratype (OSUC 239055) **297** *Odontacolus zborowskii*, netrion, lateral view, male paratype (OSUC 237998) **298** *Odontacolus australiensis*, netrion, lateral view, maleparatype (OSUC 239142). Scale bars in mm.

**Figures 299–304.**
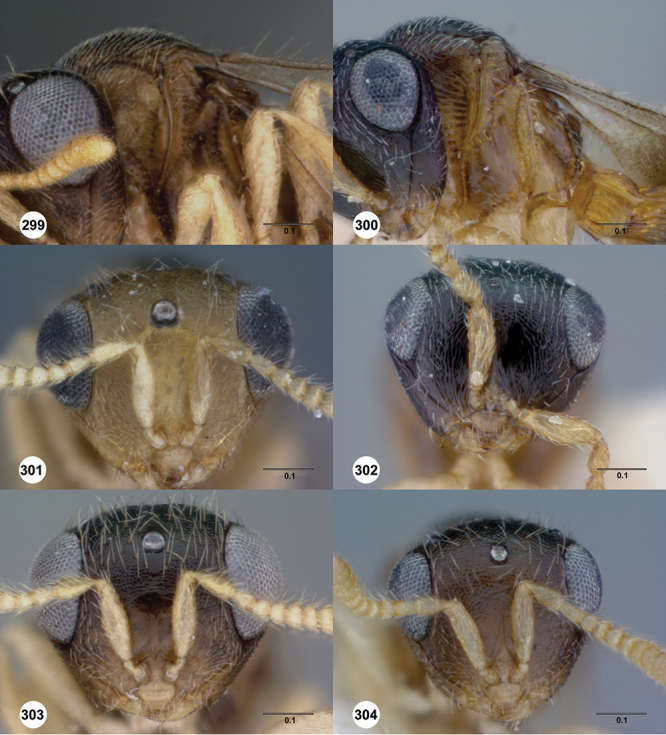
184 **299** *Odontacolus africanus*, netrion, lateral view, male paratype (OSUC 238568) **300** *Odontacolus pintoi*, netrion, lateral view, maleparatype (OSUC 239055) **301** *Odontacolus wallacei*, face, anterior view, male paratype (OSUC 238004) **302** *Odontacolus pintoi*, face, anterior view, maleparatype (OSUC 239055) **303** *Odontacolus africanus*, face, anterior view, male paratype (OSUC 238568) **304** *Odontacolus whitfieldi*, face, anterior view, male paratype (UCRCENT171091). Scale bars in mm.

**Figures 305–308.**
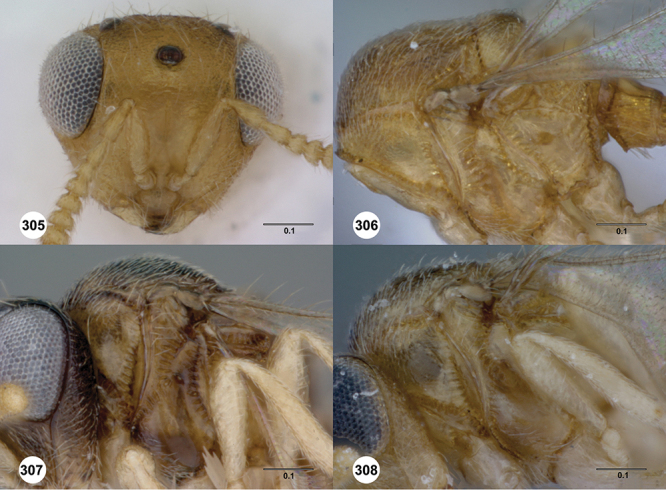
185 **305** *Odontacolus markadicus*, face, anterior view, male, (OSUC 238805) **306** *Odontacolus markadicus*, mesosoma, lateral view, male, (OSUC 238805) **307** *Odontacolus africanus*, mesosoma, lateral view, male paratype (OSUC 238568) **308** *Odontacolus wallacei*, mesosoma, lateral view, male paratype (OSUC 238013). Scale bars in mm.

**Figures 309–314.**
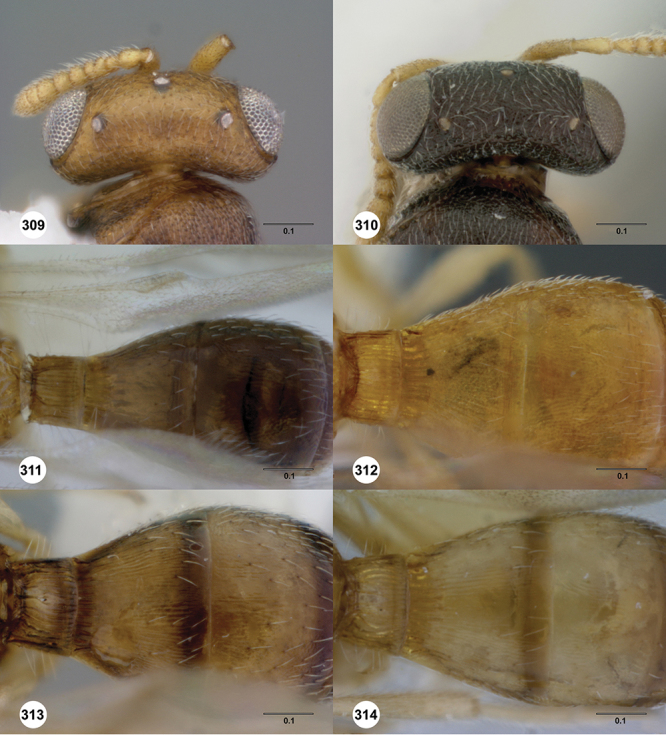
186 **309** *Odontacolus zborowskii*, head, dorsal view, male paratype(OSUC 237998) **310** *Odontacolus australiensis*, head, dorsal view, maleparatype (OSUC 239142) **311** *Odontacolus whitfieldi*, T2 dorsal view, male paratype (UCRCENT171091) **312** *Odontacolus markadicus*, T2 dorsal view, male (OSUC 238805) **313** *Odontacolus africanus*, T2 dorsal view, male paratype (OSUC 238568) **314** *Odontacolus wallacei*, T2 dorsal view, male paratype (OSUC 238013). Scale bars in mm.

**Figures 315–318.**
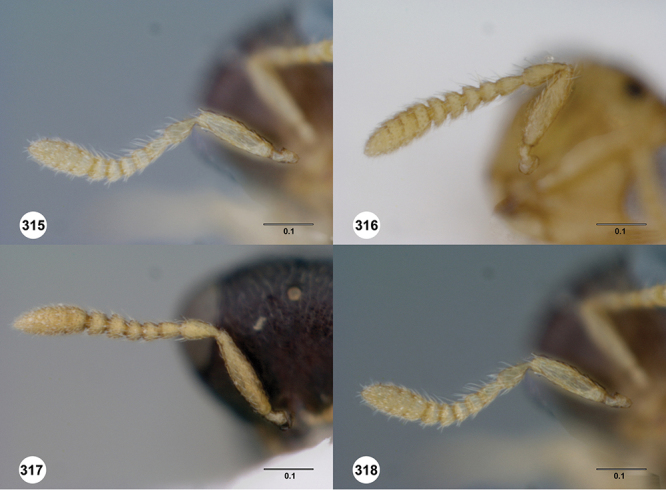
187 **315** *Odontacolus whitfieldi*, antenna, lateral view, male paratype (UCRCENT171091) **316** *Odontacolus markadicus*, antenna, lateral view, male (OSUC 238805) **317** *Odontacolus australiensis*, antenna, lateral view, male paratype (OSUC 239142) **318** *Odontacolus pintoi*, antenna, lateral view, male (OSUC 239055). Scale bars in mm.

**Figures 319–324.**
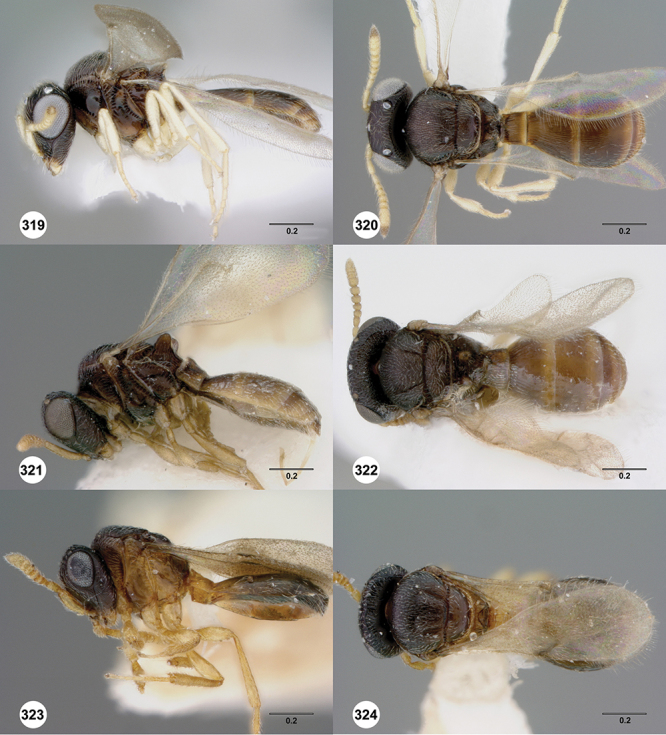
188 **319** *Odontacolus africanus*, lateral habitus, male paratype (CASENT 2042643) **320** *Odontacolus africanus*, dorsal habitus, male paratype (CASENT 2042643) **321** *Odontacolus australiensis*, lateral habitus, male paratype (OSUC 239142) **322** *Odontacolus australiensis*, dorsal habitus, male paratype (OSUC 239142) **323** *Odontacolus pintoi*, lateral habitus, male paratype (OSUC 239055) **324** *Odontacolus pintoi*, dorsal habitus, male paratype (OSUC 239055). Scale bars in mm.

**Figures 325–330.**
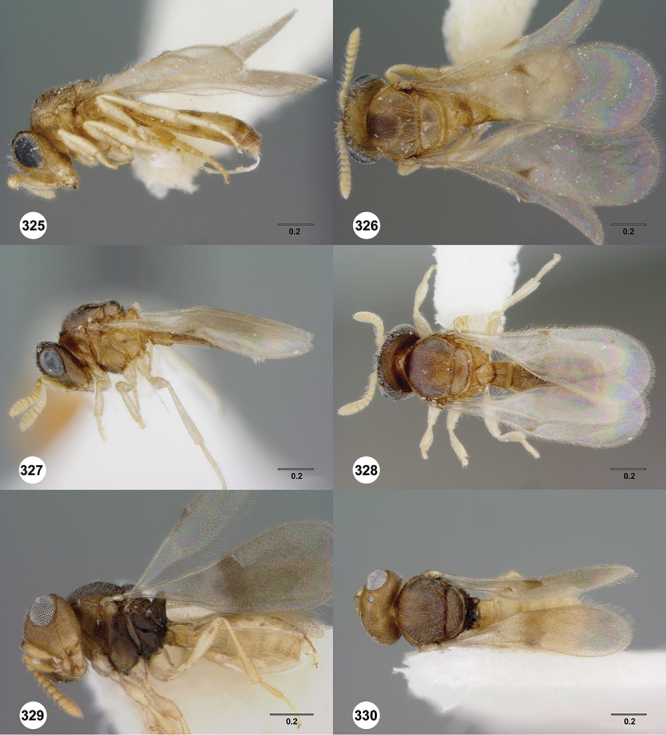
189 **325** *Odontacolus wallacei*, lateral habitus, male paratype (OSUC 238013) **326** *Odontacolus wallacei*, dorsal habitus, male paratype (OSUC 238013) **327** *Odontacolus whitfieldi*, lateral habitus, male paratype (UCRCENT 171086) **328** *Odontacolus whitfieldi*, dorsal habitus, male paratype (UCRCENT 171086) **329** *Odontacolus zborowskii*, lateral habitus, male paratype (OSUC 237998) **330** *Odontacolus zborowskii*, dorsal habitus, male paratype (OSUC 237998). Scale bars in mm.

**Figures 331–332.**
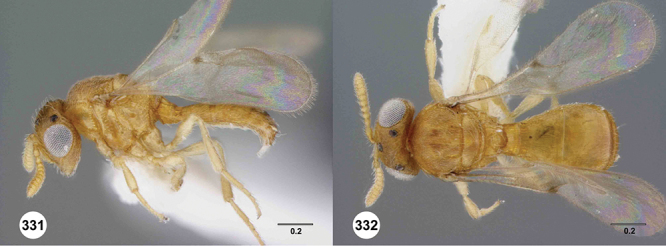
190 *Odontacolus markadicus* **331** Lateral habitus, male (OSUC 238805) **332** Dorsal habitus, male (OSUC 238805). Scale bars in mm.

## Supplementary Material

XML Treatment for Odontacolus

XML Treatment for Odontacolus africanus

XML Treatment for Odontacolus aldrovandii

XML Treatment for Odontacolus anningae

XML Treatment for Odontacolus australiensis

XML Treatment for Odontacolus baeri

XML Treatment for Odontacolus berryae

XML Treatment for Odontacolus bosei

XML Treatment for Odontacolus cardaleae

XML Treatment for Odontacolus darwini

XML Treatment for Odontacolus dayi

XML Treatment for Odontacolus gallowayi

XML Treatment for Odontacolus gentingensis

XML Treatment for Odontacolus guineensis

XML Treatment for Odontacolus hackeri

XML Treatment for Odontacolus harveyi

XML Treatment for Odontacolus heratyi

XML Treatment for Odontacolus heydoni

XML Treatment for Odontacolus irwini

XML Treatment for Odontacolus jacksonae

XML Treatment for Odontacolus kiau

XML Treatment for Odontacolus lamarcki

XML Treatment for Odontacolus longiceps

XML Treatment for Odontacolus madagascarensis

XML Treatment for Odontacolus markadicus

XML Treatment for Odontacolus mayri

XML Treatment for Odontacolus mot

XML Treatment for Odontacolus noyesi

XML Treatment for Odontacolus pintoi

XML Treatment for Odontacolus schlingeri

XML Treatment for Odontacolus sharkeyi

XML Treatment for Odontacolus spinosus

XML Treatment for Odontacolus veroae

XML Treatment for Odontacolus wallacei

XML Treatment for Odontacolus whitfieldi

XML Treatment for Odontacolus zborowskii

XML Treatment for Odontacolus zimi

## Endnotes

1 http://biocol.org/urn:lsid:biocol.org:col:32976

2 http://biocol.org/urn:lsid:biocol.org:col:32981

3 http://biocol.org/urn:lsid:biocol.org:col:1009

4 http://biocol.org/urn:lsid:biocol.org:col:33071

5 http://biocol.org/urn:lsid:biocol.org:col:33138

6 http://biocol.org/urn:lsid:biocol.org:col:1013

7 http://biocol.org/urn:lsid:biocol.org:col:33357

8 http://biocol.org/urn:lsid:biocol.org:col:33453

9 http://biocol.org/urn:lsid:biocol.org:col:33532

10 http://biocol.org/urn:lsid:biocol.org:col:33729

11 http://biocol.org/urn:lsid:biocol.org:col:35292

12 http://biocol.org/urn:lsid:biocol.org:col:34022

13 http://biocol.org/urn:lsid:biocol.org:col:34070

14 http://biocol.org/urn:lsid:biocol.org:col:1014

15 http://biocol.org/urn:lsid:biocol.org:col:34157

16 http://biocol.org/urn:lsid:biocol.org:col:34163

17 http://biocol.org/urn:lsid:biocol.org:col:1016

18 http://biocol.org/urn:lsid:biocol.org:col:34212

19 http://biocol.org/urn:lsid:biocol.org:col:1017

20 http://biocol.org/urn:lsid:biocol.org:col:34245

21 http://biocol.org/urn:lsid:biocol.org:col:34403

22 http://biocol.org/urn:lsid:biocol.org:col:34424

23 http://biocol.org/urn:lsid:biocol.org:col:1019

24 http://biocol.org/urn:lsid:biocol.org:col:34579

25 http://biocol.org/urn:lsid:biocol.org:col:34593

26 http://vsyslab.osu.edu

27 http://www.gbif.org/informatics/standards-and-tools/publishing-data/data-standards/darwin-core-archives/

28 http://www.morphbank.net

29 http://specimage.osu.edu

30 http://hol.osu.edu/map-full.html?id=525

31 http://www.morphbank.net/?id=781328

32 http://hol.osu.edu/map-full.html?id=254265

33 http://www.morphbank.net/?id=781327

34 http://hol.osu.edu/map-full.html?id=254276

35 http://www.morphbank.net/?id=781326

36 http://hol.osu.edu/map-full.html?id=281688

37 http://www.morphbank.net/?id=781325

38 http://hol.osu.edu/map-full.html?id=254263

39 http://www.morphbank.net/?id=781324

40 http://hol.osu.edu/map-full.html?id=281689

41 http://www.morphbank.net/?id=781323

42 http://hol.osu.edu/map-full.html?id=254262

43 http://www.morphbank.net/?id=781322

44 http://hol.osu.edu/map-full.html?id=254277

45 http://www.morphbank.net/?id=781321

46 http://hol.osu.edu/map-full.html?id=254270

47 http://www.morphbank.net/?id=781320

48 http://hol.osu.edu/map-full.html?id=254272

49 http://www.morphbank.net/?id=781319

50 http://hol.osu.edu/map-full.html?id=254328

51 http://www.morphbank.net/?id=781317

52 http://hol.osu.edu/map-full.html?id=254260

53 http://www.morphbank.net/?id=781316

54 http://hol.osu.edu/map-full.html?id=254259

55 http://www.morphbank.net/?id=781315

56 http://hol.osu.edu/map-full.html?id=254257

57 http://www.morphbank.net/?id=781314

58 http://hol.osu.edu/map-full.html?id=4947

59 http://www.morphbank.net/?id=781313

60 http://hol.osu.edu/map-full.html?id=254264

61 http://www.morphbank.net/?id=781312

62 http://hol.osu.edu/map-full.html?id=254268

63 http://www.morphbank.net/?id=781311

64 http://hol.osu.edu/map-full.html?id=254271

65 http://www.morphbank.net/?id=781310

66 http://hol.osu.edu/map-full.html?id=254275

67 http://www.morphbank.net/?id=781309

68 http://hol.osu.edu/map-full.html?id=254256

69 http://www.morphbank.net/?id=781308

70 http://hol.osu.edu/map-full.html?id=280211

71 http://www.morphbank.net/?id=781307

72 http://hol.osu.edu/map-full.html?id=280244

73 http://www.morphbank.net/?id=781306

74 http://hol.osu.edu/map-full.html?id=4948

75 http://www.morphbank.net/?id=781305

76 http://hol.osu.edu/map-full.html?id=254269

77 http://www.morphbank.net/?id=781297

78 http://hol.osu.edu/map-full.html?id=254255

79 http://www.morphbank.net/?id=781304

80 http://hol.osu.edu/map-full.html?id=284013

81 http://www.morphbank.net/?id=781303

82 http://hol.osu.edu/map-full.html?id=283809

83 http://www.morphbank.net/?id=781302

84 http://hol.osu.edu/map-full.html?id=254258

85 http://www.morphbank.net/?id=781301

86 http://hol.osu.edu/map-full.html?id=254267

87 http://www.morphbank.net/?id=781300

88 http://hol.osu.edu/map-full.html?id=254274

89 http://www.morphbank.net/?id=781299

90 http://hol.osu.edu/map-full.html?id=275238

91 http://www.morphbank.net/?id=781298

92 http://hol.osu.edu/map-full.html?id=4951

93 http://www.morphbank.net/?id=781296

94 http://hol.osu.edu/map-full.html?id=254273

95 http://www.morphbank.net/?id=781295

96 http://hol.osu.edu/map-full.html?id=254261

97 http://www.morphbank.net/?id=781294

98 http://hol.osu.edu/map-full.html?id=278397

99 http://www.morphbank.net/?id=781293

100 http://hol.osu.edu/map-full.html?id=783503

101 http://www.morphbank.net/?id=781293

102 http://hol.osu.edu/map-full.html?id=302168

103 http://lsid.tdwg.org/urn:lsid:biosci.ohio-state.edu:osuc_pubs:105

104 http://lsid.tdwg.org/urn:lsid:biosci.ohio-state.edu:osuc_pubs:22938

105 http://lsid.tdwg.org/urn:lsid:biosci.ohio-state.edu:osuc_pubs:22939

106 http://lsid.tdwg.org/urn:lsid:biosci.ohio-state.edu:osuc_pubs:114

107 http://lsid.tdwg.org/urn:lsid:biosci.ohio-state.edu:osuc_pubs:21013

108 http://lsid.tdwg.org/urn:lsid:biosci.ohio-state.edu:osuc_pubs:21126

109 http://lsid.tdwg.org/urn:lsid:biosci.ohio-state.edu:osuc_pubs:1240

110 http://lsid.tdwg.org/urn:lsid:biosci.ohio-state.edu:osuc_pubs:562

111 http://lsid.tdwg.org/urn:lsid:biosci.ohio-state.edu:osuc_pubs:314

112 http://lsid.tdwg.org/urn:lsid:biosci.ohio-state.edu:osuc_pubs:410

113 http://lsid.tdwg.org/urn:lsid:biosci.ohio-state.edu:osuc_pubs:339

114 http://lsid.tdwg.org/urn:lsid:biosci.ohio-state.edu:osuc_pubs:797

115 http://lsid.tdwg.org/urn:lsid:biosci.ohio-state.edu:osuc_pubs:22787

116 http://lsid.tdwg.org/urn:lsid:biosci.ohio-state.edu:osuc_pubs:229

117 http://lsid.tdwg.org/urn:lsid:biosci.ohio-state.edu:osuc_pubs:23466

118 http://lsid.tdwg.org/urn:lsid:biosci.ohio-state.edu:osuc_pubs:386

119 http://lsid.tdwg.org/urn:lsid:biosci.ohio-state.edu:osuc_pubs:309

120 http://lsid.tdwg.org/urn:lsid:biosci.ohio-state.edu:osuc_pubs:387

121 http://lsid.tdwg.org/urn:lsid:biosci.ohio-state.edu:osuc_pubs:328

122 http://lsid.tdwg.org/urn:lsid:biosci.ohio-state.edu:osuc_pubs:310

123 http://lsid.tdwg.org/urn:lsid:biosci.ohio-state.edu:osuc_pubs:9564

124 http://lsid.tdwg.org/urn:lsid:biosci.ohio-state.edu:osuc_pubs:9725

125 http://lsid.tdwg.org/urn:lsid:biosci.ohio-state.edu:osuc_pubs:21243

126 http://lsid.tdwg.org/urn:lsid:biosci.ohio-state.edu:osuc_pubs:311

127 http://lsid.tdwg.org/urn:lsid:biosci.ohio-state.edu:osuc_pubs:1936

128 http://lsid.tdwg.org/urn:lsid:biosci.ohio-state.edu:osuc_pubs:474

129 http://lsid.tdwg.org/urn:lsid:biosci.ohio-state.edu:osuc_pubs:21300

130 http://lsid.tdwg.org/urn:lsid:biosci.ohio-state.edu:osuc_pubs:240

131 http://lsid.tdwg.org/urn:lsid:biosci.ohio-state.edu:osuc_pubs:345

132 http://lsid.tdwg.org/urn:lsid:biosci.ohio-state.edu:osuc_pubs:21213

133 http://lsid.tdwg.org/urn:lsid:biosci.ohio-state.edu:osuc_pubs:23164

134 http://lsid.tdwg.org/urn:lsid:biosci.ohio-state.edu:osuc_pubs:23652

135 http://www.morphbank.net/?id=781329

136 http://www.morphbank.net/?id=781383

137 http://www.morphbank.net/?id=781382

138 http://www.morphbank.net/?id=781381

139 http://www.morphbank.net/?id=781380

140 http://www.morphbank.net/?id=781379

141 http://www.morphbank.net/?id=781378

142 http://www.morphbank.net/?id=781377

143 http://www.morphbank.net/?id=781376

144 http://www.morphbank.net/?id=781375

145 http://www.morphbank.net/?id=781374

146 http://www.morphbank.net/?id=781338

147 http://www.morphbank.net/?id=781331

148 http://www.morphbank.net/?id=781332

149 http://www.morphbank.net/?id=781333

150 http://www.morphbank.net/?id=781334

151 http://www.morphbank.net/?id=781335

152 http://www.morphbank.net/?id=781336

153 http://www.morphbank.net/?id=781337

154 http://www.morphbank.net/?id=781339

155 http://www.morphbank.net/?id=781340

156 http://www.morphbank.net/?id=781318

157 http://www.morphbank.net/?id=781341

158 http://www.morphbank.net/?id=781342

159 http://www.morphbank.net/?id=781343

160 http://www.morphbank.net/?id=781344

161 http://www.morphbank.net/?id=781345

162 http://www.morphbank.net/?id=781346

163 http://www.morphbank.net/?id=781347

164 http://www.morphbank.net/?id=781348

165 http://www.morphbank.net/?id=781349

166 http://www.morphbank.net/?id=781350

167 http://www.morphbank.net/?id=781351

168 http://www.morphbank.net/?id=781352

169 http://www.morphbank.net/?id=781353

170 http://www.morphbank.net/?id=781361

171 http://www.morphbank.net/?id=781354

172 http://www.morphbank.net/?id=781355

173 http://www.morphbank.net/?id=781356

174 http://www.morphbank.net/?id=781357

175 http://www.morphbank.net/?id=781358

176 http://www.morphbank.net/?id=781359

177 http://www.morphbank.net/?id=781360

178 http://www.morphbank.net/?id=781362

179 http://www.morphbank.net/?id=781363

180 http://www.morphbank.net/?id=781364

181 http://www.morphbank.net/?id=781365

182 http://www.morphbank.net/?id=783503

183 http://www.morphbank.net/?id=781366

184 http://www.morphbank.net/?id=781367

185 http://www.morphbank.net/?id=781368

186 http://www.morphbank.net/?id=781369

187 http://www.morphbank.net/?id=781370

188 http://www.morphbank.net/?id=781371

189 http://www.morphbank.net/?id=781372

190 http://www.morphbank.net/?id=781373

## Appendix I

**Table:** Hymenoptera Ontology terminology used on this publication.<br/>

antenna	http://purl.obolibrary.org/obo/HAO_0000101
antennal scrobe	http://purl.obolibrary.org/obo/HAO_0001432
anterior ocellus	http://purl.obolibrary.org/obo/HAO_0000526
area, areas	http://purl.obolibrary.org/obo/HAO_0000146
body	http://purl.obolibrary.org/obo/HAO_0000182
carina, carinae	http://purl.obolibrary.org/obo/HAO_0000188
central keel	http://purl.obolibrary.org/obo/HAO_0000109
compound eyes	http://purl.obolibrary.org/obo/HAO_0000217
costae	http://purl.obolibrary.org/obo/HAO_0000225
costulae	http://purl.obolibrary.org/obo/HAO_0000486
coxae	http://purl.obolibrary.org/obo/HAO_0000228
depression	http://purl.obolibrary.org/obo/HAO_0000241
edge	http://purl.obolibrary.org/obo/HAO_0000285
eyes	http://purl.obolibrary.org/obo/HAO_0000217
face	http://purl.obolibrary.org/obo/HAO_0000316
fore wing	http://purl.obolibrary.org/obo/HAO_0000351
frons	http://purl.obolibrary.org/obo/HAO_0001523
furrow, furrows	http://purl.obolibrary.org/obo/HAO_0000978
gena	http://purl.obolibrary.org/obo/HAO_0000371
head	http://purl.obolibrary.org/obo/HAO_0000397
lateral ocellus, lateral ocelli	http://purl.obolibrary.org/obo/HAO_0000481
lateral propodeal areas	http://purl.obolibrary.org/obo/HAO_0000485
legs	http://purl.obolibrary.org/obo/HAO_0000494
malar space	http://purl.obolibrary.org/obo/HAO_0000503
mandible	http://purl.obolibrary.org/obo/HAO_0000506
margins	http://purl.obolibrary.org/obo/HAO_0000510
mesepimeron	http://purl.obolibrary.org/obo/HAO_0000751
mesepisternum	http://purl.obolibrary.org/obo/HAO_0000541
mesonotum	http://purl.obolibrary.org/obo/HAO_0000556
mesoscutellum	http://purl.obolibrary.org/obo/HAO_0000574
mesoscutum	http://purl.obolibrary.org/obo/HAO_0000575
mesoscutum length	http://purl.obolibrary.org/obo/HAO_0001912
mesosoma	http://purl.obolibrary.org/obo/HAO_0000576
metapleuron	http://purl.obolibrary.org/obo/HAO_0001271
metasoma	http://purl.obolibrary.org/obo/HAO_0000626
netrion	http://purl.obolibrary.org/obo/HAO_0000644
notaulus	http://purl.obolibrary.org/obo/HAO_0000647
occipital carina	http://purl.obolibrary.org/obo/HAO_0000653
occiput	http://purl.obolibrary.org/obo/HAO_0000658
ocellus, ocelli	http://purl.obolibrary.org/obo/HAO_0000661
orbital carina	http://purl.obolibrary.org/obo/HAO_0000674
patch	http://purl.obolibrary.org/obo/HAO_0000704
plate	http://purl.obolibrary.org/obo/HAO_0000909
posterior ocellus	http://purl.obolibrary.org/obo/HAO_0000481
pronotum	http://purl.obolibrary.org/obo/HAO_0000853
propleuron	http://purl.obolibrary.org/obo/HAO_0000862
propodeum	http://purl.obolibrary.org/obo/HAO_0001249
ridges	http://purl.obolibrary.org/obo/HAO_0000188
scrobe	http://purl.obolibrary.org/obo/HAO_0000912
sculpture	http://purl.obolibrary.org/obo/HAO_0000913
seta	http://purl.obolibrary.org/obo/HAO_0000935
spines	http://purl.obolibrary.org/obo/HAO_0000949
tegula	http://purl.obolibrary.org/obo/HAO_0000993
terga	http://purl.obolibrary.org/obo/HAO_0001006
torular triangle	http://purl.obolibrary.org/obo/HAO_0001021
vein	http://purl.obolibrary.org/obo/HAO_0001095
vertex	http://purl.obolibrary.org/obo/HAO_0001077
wing vein	http://purl.obolibrary.org/obo/HAO_0001095
wing, wings	http://purl.obolibrary.org/obo/HAO_0001089

## Appendix II

Characters and their states used in the phylogenetic analysis of *Odontacolus* species. Figures that illustrate the character states are given at the end of each character between square brackets when possible, e.g. [23], for article image number 23.

***Female characters***

Head

1. Lower head shape in anterior view

0. moderately short and strongly narrowed towards mouth, head appearing short and broad [101]

1. elongate and broad at mouth, head appearing elongate and somewhat thin [77]

2. Lagrimal

0. absent [35]

1. present, large [36]

2. present, small

3. Compound eye size in relation to head height (in anterior view)

0. approximately 1/2 or more [75]

1. approximately 1/3 or less [219]

4. Antennal scrobe sculpture

0. smooth or nearly so [87]

1. with granulate, coriaceous or rugulose sculpture [171, 273]

2. with confuses sinuate transverse costate sculpture throughout [225]

3. upper half with semicircular fine ridges, remainder smooth

4. almost completely cover with semicircular fine ridges

5. covered with vertical carinae

5. Surface of torular triangle

0. slightly bulging [117]

1. flat [287]

6. Central keel on frons

0. absent [193]

1. present (partially or throughout antennal scrobe height) [75]

7. Mandibular teeth shape

0. all subequal in length

1. middle tooth clearly longer than upper and lower teeth

2. upper and middle teeth subequal in length, lower tooth conspicuously smaller than upper two

8. Malar space sculpturing

0. smooth or mainly smooth, without fan-like sculpture

1. only with conspicuous fan-like sculpture

2. completely cover by sinuate, transverse, fine ridges

3. almost completely cover with semicircular, fine ridges from antennal scrobe

4. completely cover by granulate sculpture, without fan-like sculpture

5. with sparse, fine, dorsoventral carinae, weak granulae in background

6. weakly rugulose throughout, without fan-like striae

7. with sparse, short, fan-like striae mixed with weak coriaceous sculpture

8. with weak rugulose sculpture mixed with granulate sculpture

9. Surface of medial area of vertex

0. slightly depressed [43]

1. slightly round [39]

2. conspicuously depressed

10. Compound eye setosity

0. not evident, if observable at 60× magnification then very short and sparse [285]

1. clearly long and dense [10]

2. short and dense

11. Lateral ocellar size

0. smaller than the combined length of the 3 adjacent ommatidia of the compound eye [290]

1. larger than the combined length of the 3 adjacent ommatidia of the compound eye [15]

12. Distance between lateral ocellus and occipital carina

0. less or equal to 0.49× of ocellar diameter [11]

1. between 0.5× and 1.2× of ocellar diameter [31]

2. larger than 1.5× of ocellar diameter [29]

13. Distance between lateral ocellus and compound eye

0. 0.33× ocellar diameter or less [15]

1. approximately 0.5× ocellar diameter

2. approximately 0.66× ocellar diameter

3. 1× or more ocellar diameter or more

14. Surface of vertex

0. evenly rounded [29]

1. conspicuously bent medially [43]

15. Distance between occipital carina to orbital carina

0. well separated by distance at least 2× width of occipital carina [32]

1. separated by distance slightly greater than width of occipital carina [216]

2. contiguous or nearly so, separated by distance subequal to width of occipital carina [290]

16. Shape of midupper area of occipital carina

0. well-defined and sharply bent down, “M” shaped [245]

1. well-defined and slightly bent medially [182]

2. well-defined and slightly curved upwards [2]

3. carina virtually absent, not well-defined

Mesosoma

17. Netrion

1. present, well-defined [20]

2. represented only by sculpture at lateral pronotal areas [19]

18. Notauli

0. completely absent [182]

1. present as a smooth furrow [188]

2. completely obscured due to dense longitudinally costate sculpture

3. present as a furrow with transverse elements along their length [284]

19. Mesoscutum sculpture

0. smooth or mainly smooth [242]

1. covered with broad longitudinal sculpture

2. covered with just one type of sculpture other than longitudinal or granulate sculpture [100, 128]

3. anterior area with sculpture different to remainder of mesoscutum [146]

4. covered with granulate sculpture [104]

20. Mesoscutellum sculpture

0. completely smooth

1. mainly smooth with row(s) of punctures laterally

2. mainly smooth with sculpture other than row(s) of punctures laterally

3. covered with sinuate longitudinally costate sculpture [260]

4. covered with granulate or rugulose sculpture [74, 278]

5. anterior area covered with small, dense rugula, otherwise with coarse, spaced rugulae [284]

6. covered with coriaceous sculpture [212]

21. Mesoscutellum shape

0. flat, conspicuously wider than long, midposterior area not depressed [212]

1. globose to very globose

2. flat, conspicuously wider than long, midposterior area depressed

22. Mesoscutellum posterior edge (in dorsal view)

0. lobed medially, ‘U’ shaped

1. bent medially, ‘W’ shaped

2. more or less straight or slightly curved [212]

3. conspicuously curved

23. Lateral propodeal areas

1. only with transverse ridges (dense or not)

2. with confused coriaceous sculpture but no transverse ridges present

3. only with longitudinal ridges

4. upper half with longitudinal ridges, remainder covered with granulate sculpture

5. with confused areolate-foveate sculpture

6. with rugulose sculpture

24. Propodeal anterior spines shape (in lateral view)

0. short, small, tips round

1. elongate, of approximately uniform width throughout, and tips round

2. lobate at apex which is much wider than base

3. spines absent

4. short, broad, tips acute

5. elongate, of approximately uniform width throughout, and tips acute

25. Inter-propodeal anterior spine area (in dorsal view)

0. smooth or nearly so

1. transversely costate

2. with longitudinally costate sculpture

3. with few lateral semi-transverse ridges

4. rugulose throughout

Wings

26. Fore wing veins

0. at most with basal area of subcostal vein tubular, otherwise veins absent

1. all veins present and tubular

27. Shape of wings

0. slightly convex, with slight constriction at 2/5 of its length [274]

1. very convex in lateral view, with conspicuous constriction at 2/5 of its length

2. flat, not convex, without obvious constriction at 2/5 of its length

28. Stigmal vein (r-rs)

0. absent

1. very short, less than 2.5× length of marginalis [239, 293]

2. elongate, more than 2.6× length of marginalis [227, 295]

29. Postmarginal vein (R1)

0. absent [294]

1. present, minute

2. present, short

Metasoma

30. Shape of T1 horn (posterior view)

0. elongate and narrow

1. short and broad [156]

2. short and narrow

3. elongate and broad

4. not applicable because of the absence of horn

31. T1 horn posterior lateral carinae

0. absent

1. present but not conspicuously cristate

2. present, conspicuously cristate

3. not applicable because of the absence of horn

32. Sculpturing on apical area of T1 horn (dorsal view)

0. absent (smooth)

1. with dense longitudinal carinae throughout

2. mostly smooth with few longitudinal carinae

3. not applicable because of the absence of horn

33. Sculpturing on posterior surface of T1 horn (posterior view)

0. absent (smooth)

1. mostly covered with well-defined longitudinal carinae

2. mostly smooth with few longitudinal carinae

3. with transverse ridges throughout

4. not applicable because of the absence of horn

34. Shape of T1 horn in cross section

0. subcircular or dorsoventrally compressed

1. laterally compressed

2. not applicable because of the absence of horn

35. T2 lateral carinae

0. absent

1. present but not well developed

2. conspicuously present

36. T2 length vs. length of T3

0. T2 longer than T3

1. T2 equal to T3 or virtually so [88]

2. T2 shorter than T3 [82]

37. Ovipositor shape

0. straight or curved into T1 horn [1B]

1. ovipositor bent over inside T1 horn (i.e. U-shaped) [1C]

2. T1 horn absent so ovipositor straight and shorter than metasoma [1A]

38. T1 shape

1. T1 narrow and elongate, approximately as wide as long or longer than wide [88]

2. T1 short and broad, conspicuously wider than long [76]

39. S2 anterior carinae

0. absent [68]

1. present, but inconspicuous, uninterrupted [67]

2. clearly evident, uninterrupted [69]

3. clearly evident, interrupted medially [65]

40. Metasoma shape

0. short and compact

1. elongate, not particularly constricted at T1

2. elongate, strongly constricted at T1 [72]

**Male characters**

41. Number of segments in antenna

0. with 11–12 segments

1. with 8–9 segments [315]

## Appendix III

Matrix character states used for the phylogenetic analysis of *Odontacolus* species.

Character 00000000011111111112222222222333333333344

number 12345678901234567890123456789012345678901

***Outgroups***

Idris_floris 11001101101010222024014321220020000202111

Idris_sp1 00010121110000221024002301211101300202011

Idris_sp2 00010101110100222024023321222101100102011

Idris_sp3 00011101120100122044023321220433420121201

***Ingroups***

Cyphacolus taxa

C_axfordi 00011001101210122033021100000200210112220

C_copelandi 00021002101110022213121210100221212111220

C_diazae 00031001100130022002021100100210011111020

C_harteni 00011004100110021000021000000210011112020

C_jenningsi 00001001100200002100021130100200110112120

C_leblanci 00021000100110022211121210100131112112220

C_lucianae 00021000100200022211121210100220012112220

C_normani 00041003100130022212121210100221212111220

C_sallyae 00001001100211122100021020000200210112120

C_tessae 00001001101100122132021100000210011112?20

C_tullyae 00001001101130112100021020000200110112120

C_watshami 00021002100120022211121210100222212111220

C_bouceki 00001101100200112132021010000200210112020

Old World Odontacolus s.str. taxa

Od_africanus 1000011[14]111100[01]21134026101020101111112320

Od_aldrovan 12010118111100021124021101020201212211220

Od_anningae 10000117110100121124026101021101212111320

Od_australiensis 00001101100200[01]22244020021010000110212120

Od_baeri 10010117110100021124026101020101211211020

Od_berryae 00000101100200022244023021010000210111120

Od_bosei 10000118111100021124021101020201212111220

Od_cardaleae 10020114111100021144026141020101312212220

Od_darwini 10021115111100021134021401020101212112220

Od_dayi 10001117011102021124026101020201212111220

Od_gallowayi 00000101100200012344021001010000110212120

Od_gentingen 11001111111000021134023111021001111212220

Od_guineaensis 10021116111100021124006121021001111112320

Od_hackeri 10011116111?0?1211230210?1020101312112320

Od_harveyi_sp3 00000101100200012130021021010000210210120

Od_heratyi 10011118111000111023026121021001212212020

Od_heydoni 10011114111100021144021101021001212212220

Od_irwini 10010118111100011044023401020101210211020

Od_jacksonae 10000117111100221144026501020001111111320

Od_kiau 10021116113100021124026001020001212211020

Od_lamarki 10000014111100021124023101020101212212220

Od_longiceps 100010141111000211260234[03]1020101011212220

Od_madagasc 10001110110100121126026401020202211211220

Od_markadicus 100[01]0117111100121144021101020201212111220

Od_mayri 10020116111100021144026101020101212112220

Od_mot 12000114111100021124026101020201212112220

Od_noyesi_sp2 101111171111000213440264[02]1020101111212220

Od_pintoi 10000110100200021120021401010000210211120

Od_schlingeri 10011114111100111024023001020101010212020

Od_sharkeyi 10021111111100021124026101020101011212220

Od_spinosus 10011116110100021123026141020101112212220

Od_veroae 10011114111100221034026401020101010211020

Od_wallacei 10011017110200021144021101020001111212320

Od_whitfieldi 10020116111100[01]21124026101020101212212220

Od_zborowskii 00020101100200012325021121010001110112120

Od_zimi 00000111110100221134021401022201111110220

New World Odontacolus s.str. taxa

Od_flaviss 10000111111100022132023131020101012211220

Od_szaboi 10050111111100022124026131020201012111220

Od_sp4 10000111220101002344211001221302012212220

Od_sp5 10100111110200022123021001221101012211220

Od_sp6 10000110120100022132021101010001011211220

Od_sp7 10000111121100221130021541011001312111220

## Appendix IV

Habitats associated with *Odontacolus* collecting events (taken from specimen label data)

2° forest

among undergrowth

behind vegetable garden

burnt pine

bush

campground; pond

canopy

casuarina forest

clearing mostly on scraps wood; nr. edge clearing

closed forest

coastal dunes; vegetation; sandy area

creek

creekbed

*Dipterocarpus rigidus* primary forest

dry evergreen

dry evergreen forest

dry forest

dry gallery woodland

dry scleophyll forest

dry sclerophyll

dry sclerophyll gully

dry wash; open area

dump; open forest

eucalypt & *Deplospernum* litter; gravel pits

eucalypt litter; grazing land

evergreen forest

flooded low forest

forest

forest edge

forest gap

forest w/ few large trees & good general cover

gallery forest

grape vines; corrugated cardboard

grass; roadside; floodplain

grassland

high forest

hill; evergreen forest

hilltop; road

indigenous forest margin

mangrove swamp

medium forest

midelevation oak forest

midelevation secondary forest

mixed deciduous

mixed deciduous forest

moist evergreen

open forest

open sclerophyll gully

orange leaf

pasture

pine lands

pine treefarm

*Pinus merkusii* forest

rainforest

rainforest gully

rainforest margin

rainforest; litter

rastojo forest

relict indigenous forest

riverine woodland

sand; low altitude dense humid forest

sclerophyll, *Eucalyptus* forest

scrub; pine

seasonally swampy woodland next to grassland

second growth bush

secondary rainforest

shore; gully

small undisturbed riparian forest; valley

spiny forest thicket

stream

trail

tropical dry forest

tropical dry forest on tsingy

tropical dry forest; tsingy

tropical forest; botanical garden

tropical rainforest

tuart forest

TV aerial

under bark

under gum bark

wet forest

wet grazing floor

woodland

woodland next to grassland

## Appendix V

Darwin core taxa list. (doi: 10.3897/zookeys.314.3475.app5) File format: DarwinCore Archive. **Explanation note:** Taxon checklist.

## Appendix VI

Darwin core specimen data. (doi: 10.3897/zookeys.314.3475.app6) File format: DarwinCore Archive.

**Explanation note:** Occurance data.
